# Supplementary material for: Characterizing the evolutionary dynamics of cancer proliferation in single-cell clones with SPRINTER
Source: Nat Genet. 2024 Nov 29;57(1):103–14. doi: 10.1038/s41588-024-01989-z (PMC11735394; doi:10.1038/s41588-024-01989-z)
Supplement: Supplementary file 1 — Supplementary Figs. 1–48 and Notes 1–34. [file 41588_2024_1989_MOESM1_ESM.pdf]

nature genetics

Supplementary information https://doi.org/10.1038/s41588-024-01989-z

Characterizing the evolutionary dynamics of cancer proliferation in single-cell clones with SPRINTER

In the format provided by the authors and unedited

Supplementary Information

Characterising the evolutionary dynamics of cancer proliferation in single-cell clones with SPRINTER

Contents

Supplementary Figures **4**

Supplementary Fig. 1: Replication timing is conserved for most of the genome in short consecutive segments across different subsets of normal and cancer cell lines. 4

Supplementary Fig. 2: CNAs inferred in single-cell studies are substantially larger than neighbouring genomic regions with the same replication timing. 5

Supplementary Fig. 3: Most of the genome has consistent replication timing across different normal and cancer cell lines. 6

Supplementary Fig. 4: All pairs of reference replication profiles display high correlation. 7

Supplementary Fig. 5: SPRINTER's replication-aware approach for GC correction preserves replication fluctuations in contrast to previous approaches. 8

Supplementary Fig. 6: SPRINTER infers similar GC corrections across all cells in different cell cycle phases. 9

Supplementary Fig. 7: SPRINTER's replication-aware framework enables the differentiation of RDR changes due to replication fluctuations and CNAs. 10

Supplementary Fig. 8: Cells in different cell cycle phases yield increasing total read counts. 11

Supplementary Fig. 9: Generation of an accurate ground truth dataset of cells sorted into different cell cycle phases using EdU and Hoechst labelling. 12

Supplementary Fig. 10: scDNA-seq features of the generated ground truth datasets. 13

Supplementary Fig. 11: Read counts in the ground truth datasets generated with 4,410 diploid and 4,434 tetraploid cells are compatible with expected replication fluctuations. 14

Supplementary Fig. 12: SPRINTER's erroneous S phase calls in G1 phase cells in the diploid ground truth are compatible with minor infiltrating errors. 15

Supplementary Fig. 13: The novel ground truth dataset captures cells at different stages of S phase in contrast to previous datasets. 16

Supplementary Fig. 14: SPRINTER exhibits higher accuracy and sensitivity in the identification of S phase cells than existing methods in a previously published 10X CNV Solution dataset. 17

Supplementary Fig. 15: SPRINTER exhibits high accuracy and sensitivity in identifying S phase cells from mixed subpopulations of diploid and tetraploid cells in contrast to existing methods. 18

Supplementary Fig. 16: SPRINTER is robust to a higher fraction of replication-timing errors or alterations than the maximum expected in different normal and cancer cells. 19

Supplementary Fig. 17: SPRINTER preserves high accuracy and sensitivity in identifying replicating cells when using different input replication profiles. 20

Supplementary Fig. 18: SPRINTER accurately infers G2 phase cells in both the diploid and tetraploid ground truth datasets. 21

Supplementary Fig. 19: SPRINTER results for 4,410 diploid and 4,434 tetraploid ground truth cells. 22

Supplementary Fig. 20: SPRINTER accurately assigns S phase cells to clones in contrast to previous approaches. 23

Supplementary Fig. 21: Spike-in experiment of CNAs demonstrates that SPRINTER accurately recovers most >3Mb copy-number events in both S and non-S phase cells. 24

Supplementary Fig. 22: Clinical history of patient CRUKP9145 who participated in the TRACERx study and PEACE programme........ 25

Supplementary Fig. 23: scDNA-seq features of the novel NSCLC dataset. 26

Supplementary Fig. 24: Sequencing details of all single cells sequenced from 5 primary tumour samples from patient CRUKP9145........ 27

Supplementary Fig. 25: Sequencing details of all single cells sequenced from five metastatic sites from patient CRUKP9145. 28

Supplementary Fig. 26: SPRINTER's results for cells sequenced from five primary tumour samples from patient CRUKP9145........ 29

Supplementary Fig. 27: SPRINTER's results for cells sequenced from five metastases from patient CRUKP9145. 30

Supplementary Fig. 28: SPRINTER accurately recovers most clones containing more than 30 cells. 31

Supplementary Fig. 29: Clones have significantly different S fractions compared to the average S fraction in the tumour from patient CRUKP9145. 32

Supplementary Fig. 30: Patterns of proliferation measured by SPRINTER are consistent when considering S fraction, G2 fraction, or the fraction of total replicating cells. 33

Supplementary Fig. 31: The S and G2 fractions in the NSCLC dataset have a high significant linear correlation........ 34

Supplementary Fig. 32: Ki-67 staining of tumour slides of metastases from patient CRUKP9145. 35

Supplementary Fig. 33: DLP+ nozzle-based nuclear imaging validates SPRINTER's phase predictions in primary tumour and metastatic samples from patient CRUKP9145. 36

Supplementary Fig. 34: Nuclear diameter is related to cell cycle phase in the ground truth datasets. 37

Supplementary Fig. 35: Primary tumour clones inferred by SPRINTER consistently harbour SNVs most similar to the SNVs obtained from bulk sequencing of the corresponding sample. 38

Supplementary Fig. 36: SPRINTER infers CNAs in the NSCLC dataset that are highly consistent with previous bulk studies of matched primary tumour regions. 39

Supplementary Fig. 37: SNV genotypes used to reconstruct tumour phylogeny from the primary tumour and metastatic samples from patient CRUKP9145. 40

Supplementary Fig. 38: Identification of driver mutations in the clones inferred by SPRINTER from the primary tumour and metastatic samples from patient CRUKP9145. 41

Supplementary Fig. 39: The novel NSCLC dataset provides sufficient power to identify most mutations across samples and clones. 42

Supplementary Fig. 40: Reconstructed evolution of driver mutations in the NSCLC dataset........ 43

Supplementary Fig. 41: Reconstructed evolution of CNAs for the NSCLC dataset........ 44

Supplementary Fig. 42: SPRINTER's estimates of S fractions are not affected by clone-specific altered replication timing (ART). 45

Supplementary Fig. 43: Gene set variation analysis using bulk RNA-sequencing in the TRACERx cohort supports the inferred ART for patient CRUKP9145. 46

Supplementary Fig. 44: The negative correlation between seeding genetic distance and clone proliferation rate indicates that clones with more similarity to seeding clones have increased proliferation. 47

Supplementary Fig. 45: High clone proliferation rate is linked to increased clone-specific ctDNA shedding. 48

Supplementary Fig. 46: SPRINTER infers CNAs that are highly consistent with previous analysis by Funnell et al. 49

Supplementary Fig. 47: SPRINTER's results are not affected by clone size or number of clones........ 50

Supplementary Fig. 48: Driver mutations and deletions of tumour suppressor genes (TSGs) associated with high clone proliferation. 51

Supplementary Notes 52

1 Assessing the accuracy of S and G2 phase identification 52

2 Assessing the impact of improved identification of replicating cells 52

3 Assessing the clone assignment of S phase cells........ 53

4 Consistency with previous bulk analysis of the NSCLC case 53

5 Corroborating SPRINTER's estimates with Ki-67 analysis 54

6 Corroborating SPRINTER's results with nuclear and clinical imaging 55

7 TRACERx and PEACE tumour tissue samples of a patient with NSCLC 55
8 Pathological assessment of Ki-67 56
9 Patient clinical imaging analysis 56
10 Classification of replication timing for genomic regions 56
11 Calculation of read depth ratios 58
12 Replication-aware correction of GC bias 59
13 Replication-aware copy-number segmentation 61
14 Cell-specific identification of S phase 63
15 Inference of baseline copy numbers in G0/1/2 phase cells 65
16 Inference of CNA-based clones 67
17 Correction of ploidy and clone errors 68
18 Clone-assignment of S phase cells 69
19 Inference of copy numbers in S phase cells 70
20 Identification of G2 cells per clone 71
21 Single-cell whole-genome DNA sequencing 72
22 Generation of a ground truth dataset of cell cycle-sorted cells 74
23 Processing single-cell whole-genome DNA sequencing data 75
24 Bioinformatics analysis of scDNA-seq data 75
25 Identification of SNVs and driver mutations in single-cell data 76
26 Phylogenetic analysis of SNVs and CNAs 77
27 Genotyping SNVs in single-cell clones 79
28 Reconstruction of the ancestral state of SNVs in single-cell clones 79
29 Reconstruction of metastatic migrations and seeding genetic distance 80
30 Identification of clone-specific altered replication timing 80
31 Expression analysis to support inferred ART 81
32 Analysis of ctDNA 82
33 Rates of clone-specific genomic variants in individual cells 82
34 Identifying genomic alterations enriched in high proliferation clones 83

References ***84***

TRACERx Consortium ***87***

PEACE Consortium ***89***

Supplementary Figures

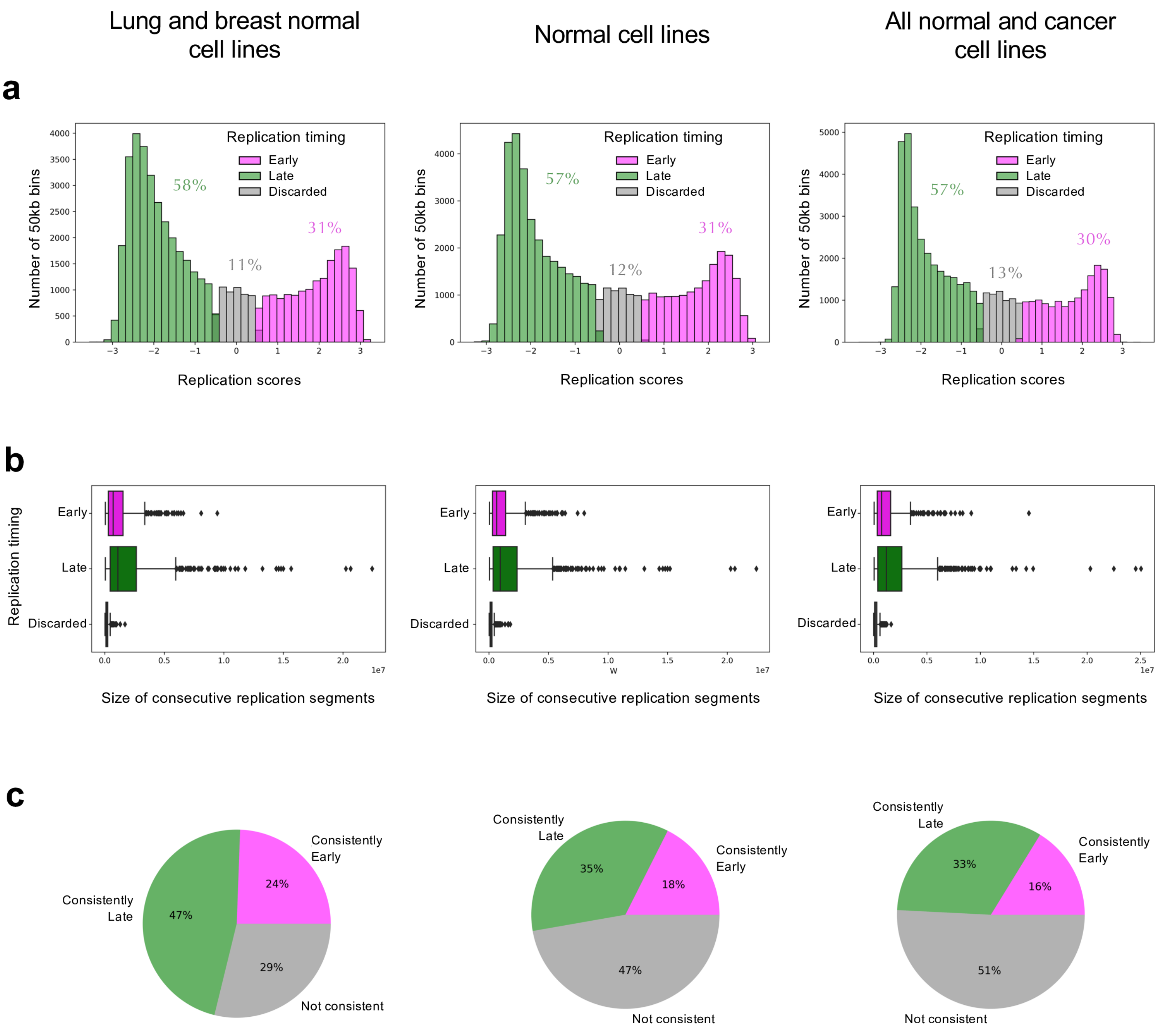

Supplementary Fig. 1: Replication timing is conserved for most of the genome in short consecutive segments across different subsets of normal and cancer cell lines. a, Average replication scores (x-axis) were measured across 50kb genomic regions (y-axis) from three different subsets of replication scores generated from Repli-Seq experiments for (left) five normal lung and breast cell lines, (middle) 10 normal cell lines across a range of tissue types, and (right) 30 normal and cancer cell lines, with early (magenta) and late (green) replicating groups defined based on an average replication score > 0.5 and < -0.5, respectively, and with the remaining regions discarded (grey). **b,** Segment sizes (x-axis, in base pairs) of neighbouring genomic regions with the same replication timing were measured for genomic regions with early, late, or unknown replication timing separately (y-axis) using the replication scores obtained from (left) five normal lung and breast cell lines, (middle) 10 normal cell lines across a range of tissue types, and (right) 30 normal and cancer cell lines. Box plots show the median and the interquartile range (IQR), and the whiskers denote the lowest and highest values within 1.5 times the IQR from the first and third quartiles, respectively. **c,** Proportion of the genome that is consistently defined to have early, late, or unknown replication timing in >95% of the cell lines across (left) five normal lung and breast cell lines, (middle) ten normal cell lines across a range of tissue types, and (right) 30 normal and cancer cell lines.

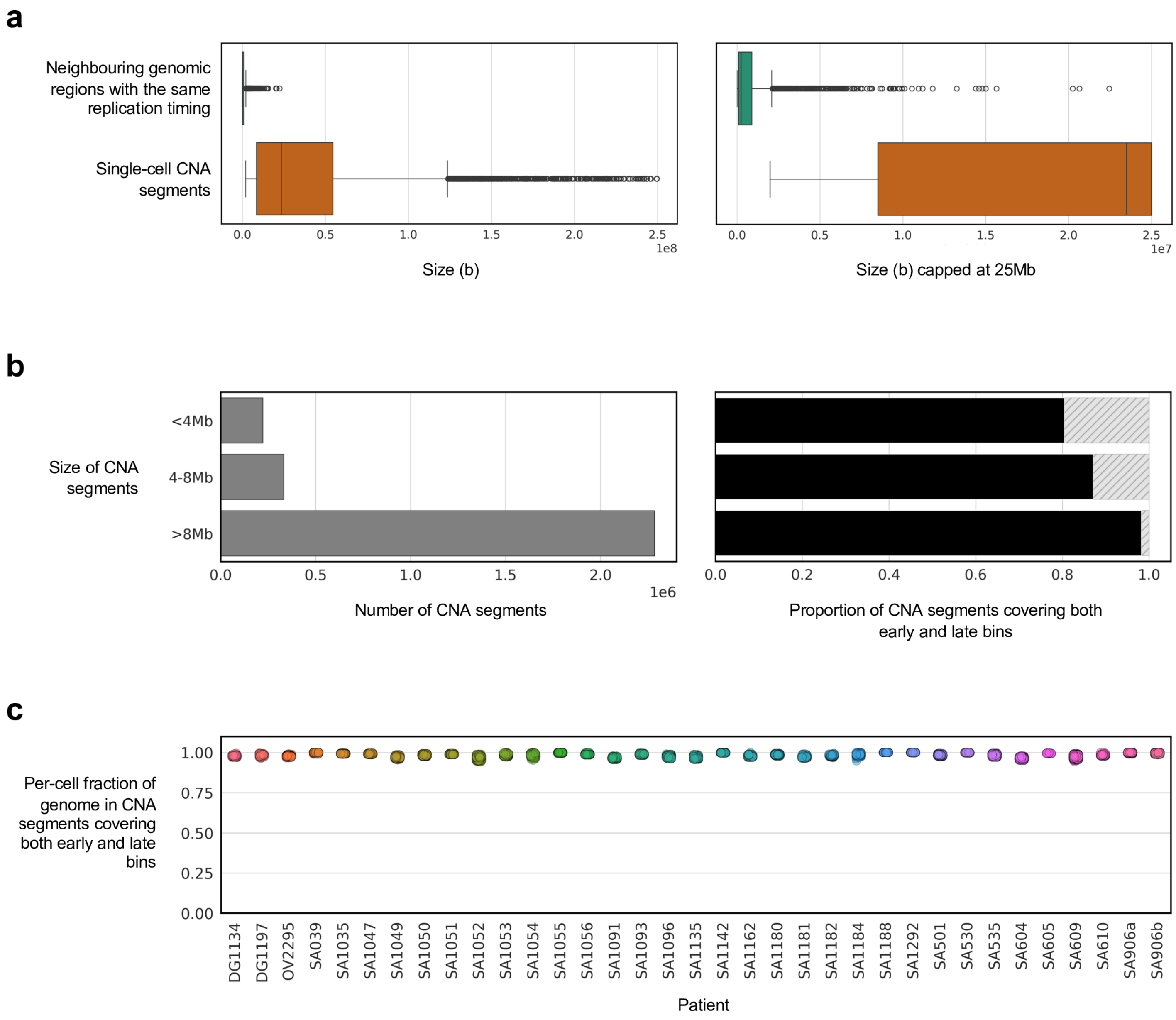

Supplementary Fig. 2: CNAs inferred in single-cell studies are substantially larger than neighbouring genomic regions with the same replication timing. **a,** The size (x-axis in bases, b) of neighbouring genomic regions with the same replication timing and of 2,968,788 previously inferred CNA segments (y-axis) is reported without (left panel) or with values capped at 25Mb (right panel) as measured from 43,106 cells in 35 patients from the previous TNBC and HGSC datasets. Box plots show the median and the interquartile range (IQR), and the whiskers denote the lowest and highest values within 1.5 times the IQR from the first and third quartiles, respectively. **b,** For the same CNA segments in (**a**), the number of segments (x-axis) within three groups of different segment sizes (y-axis) is reported (left panel) and, for each of these groups, the proportion of CNA segments covering both early and late genomic bins is also calculated (right panel). **c,** For each cell (dot) in each patient (x-axis), the fraction of the genome within CNA segments covering both early and late bins is reported (y-axis).

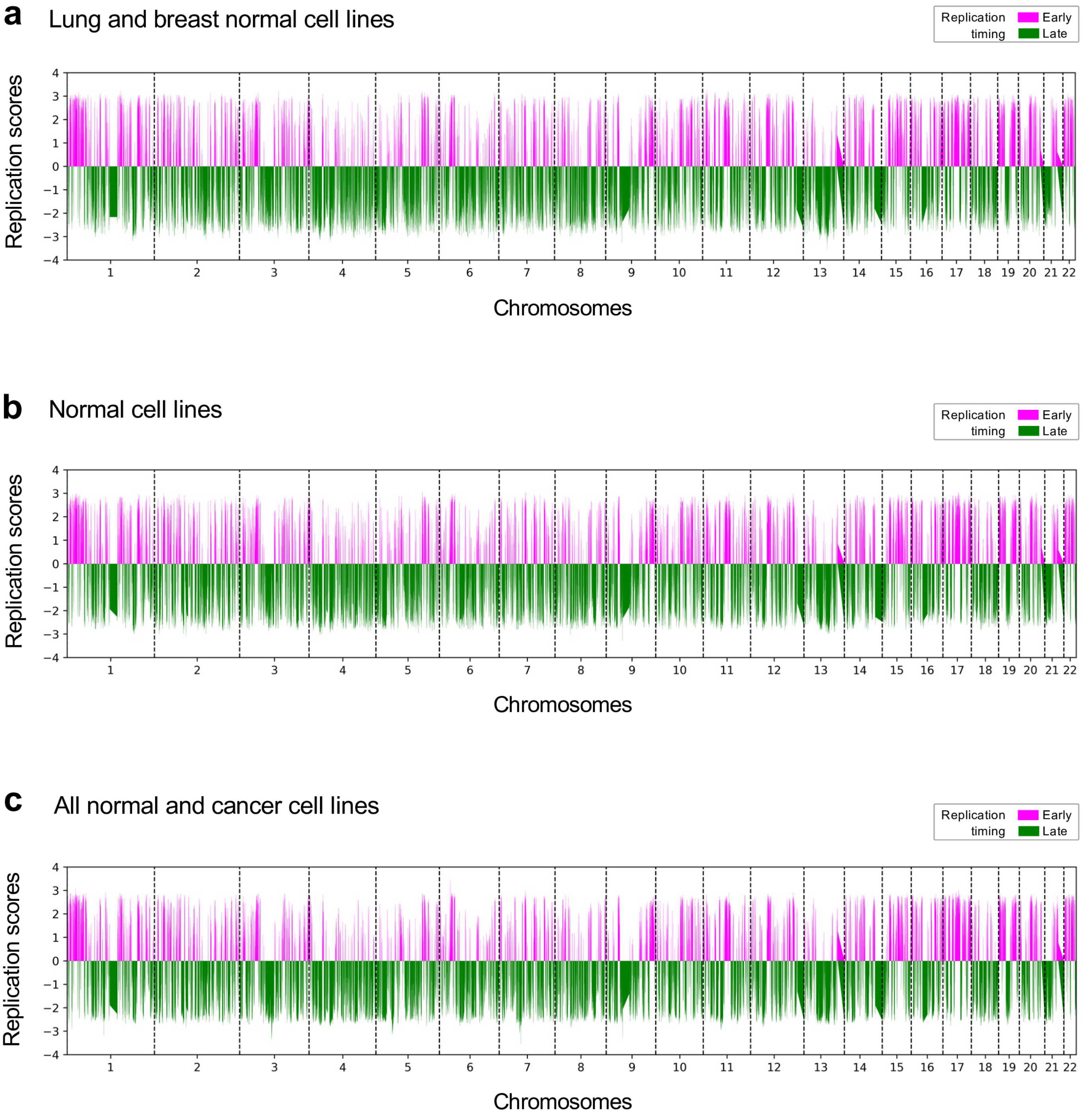

Supplementary Fig. 3: Most of the genome has consistent replication timing across different normal and cancer cell lines. Average replication scores (y-axis, measured from Repli-Seq experiments) were measured across autosomes for 50kb genomic regions (x-axis) with either early (magenta) or late (green) replication timing for (**a**) five normal lung and breast cell lines, (**b**) ten normal cell lines across a range of tissue types, and (**c**) 30 normal and cancer cell lines.

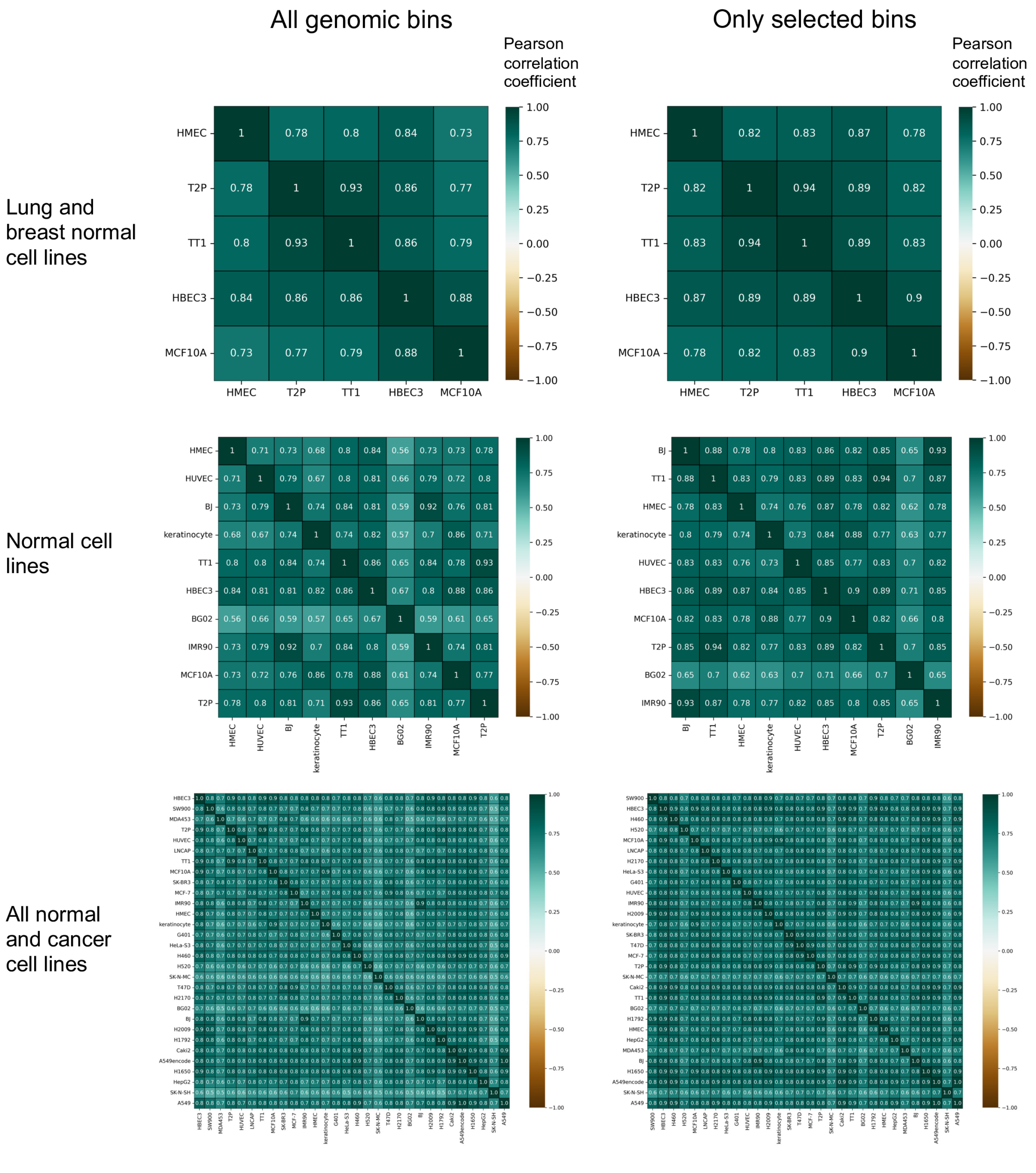

Supplementary Fig. 4: All pairs of reference replication profiles display high correlation. For the three subsets of replication profiles that can be used by SPRINTER (top, middle, and bottom), the Pearson correlation coefficient (colour) is calculated for each pair of profiles (rows and columns), considering either all genomic regions (left) or only genomic regions with conserved replication timing selected by SPRINTER using thresholds of +/-0.5 in the replication score (right).

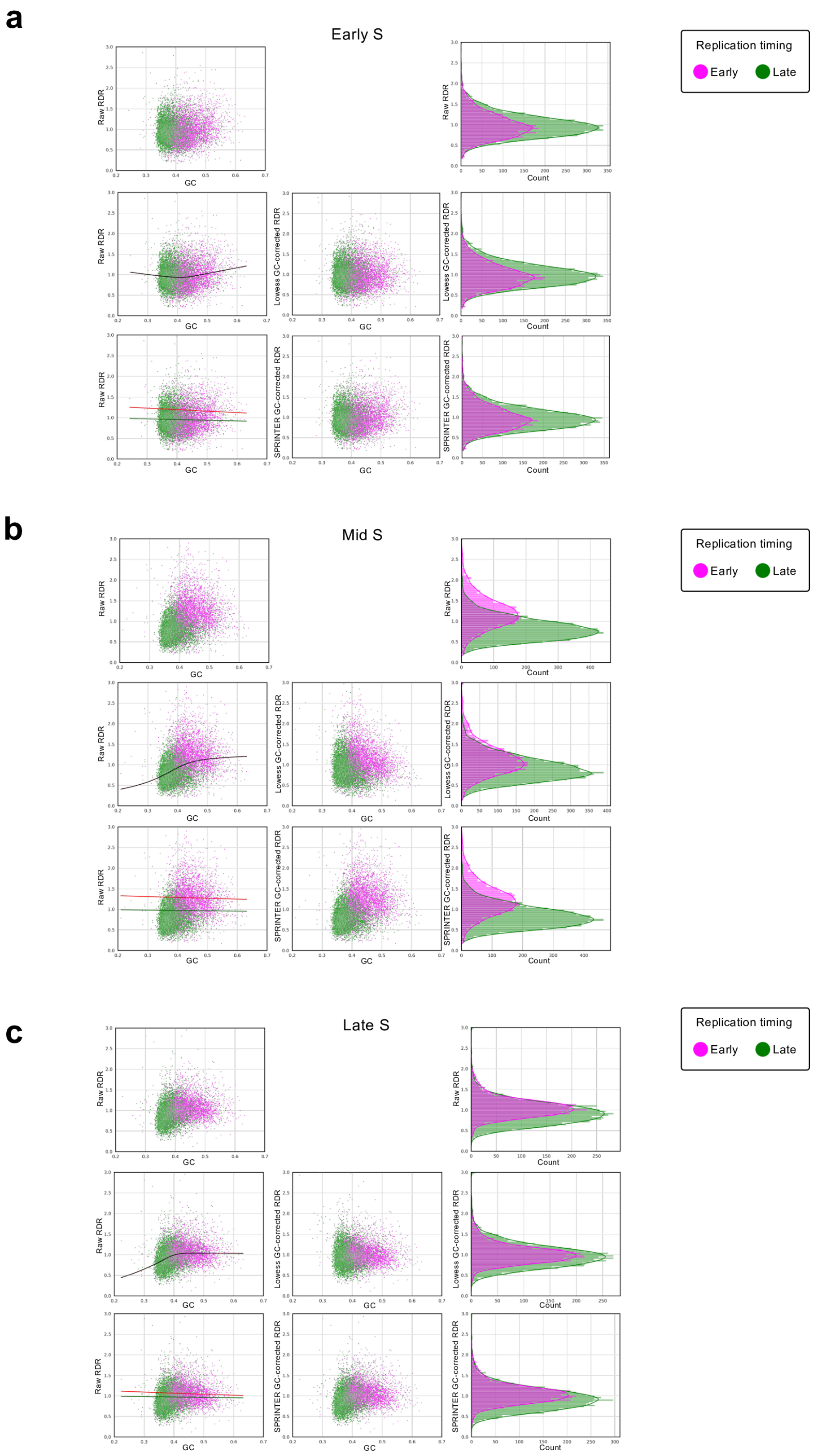

Supplementary Fig. 5: SPRINTER's replication-aware approach for GC correction preserves replication fluctuations in contrast to previous approaches. (First row of each panel) The raw RDR (y-axis) and GC content (x-axis) were measured for all 250kb bins with early (magenta) or late (green) replication timing (left plot), as well as the distribution of raw RDR (right plot), for three example cells in (**a**) early S, (**b**) mid S, and (**c**) late S phases in the diploid ground truth dataset. (Second row) For each cell, the relationship between raw RDR (y-axis) and GC content (x-axis) was computed using previous methods based on Lowess corrections (black line), and the corresponding GC-corrected RDR (y-axis) and GC content (x-axis) were computed for each bin (middle plot), as well as the corresponding distributions of the GC-corrected RDRs (right plot). (Third row) For each cell, the linear relationships between raw RDR (y-axis) and GC content (x-axis) were computed by SPRINTER's GC correction approach for either early or late replicating bins (red and green lines, respectively), and the correspondingly GC-corrected RDR (y-axis) and GC content (x-axis) were computed for each bin (middle plot), as well as the corresponding distributions of the GC-corrected RDRs (right plot). For all cells across the different cell cycle phases, SPRINTER's correction preserves a clearer separation in the GC-corrected RDRs between early and late replicating bins than previous methods.

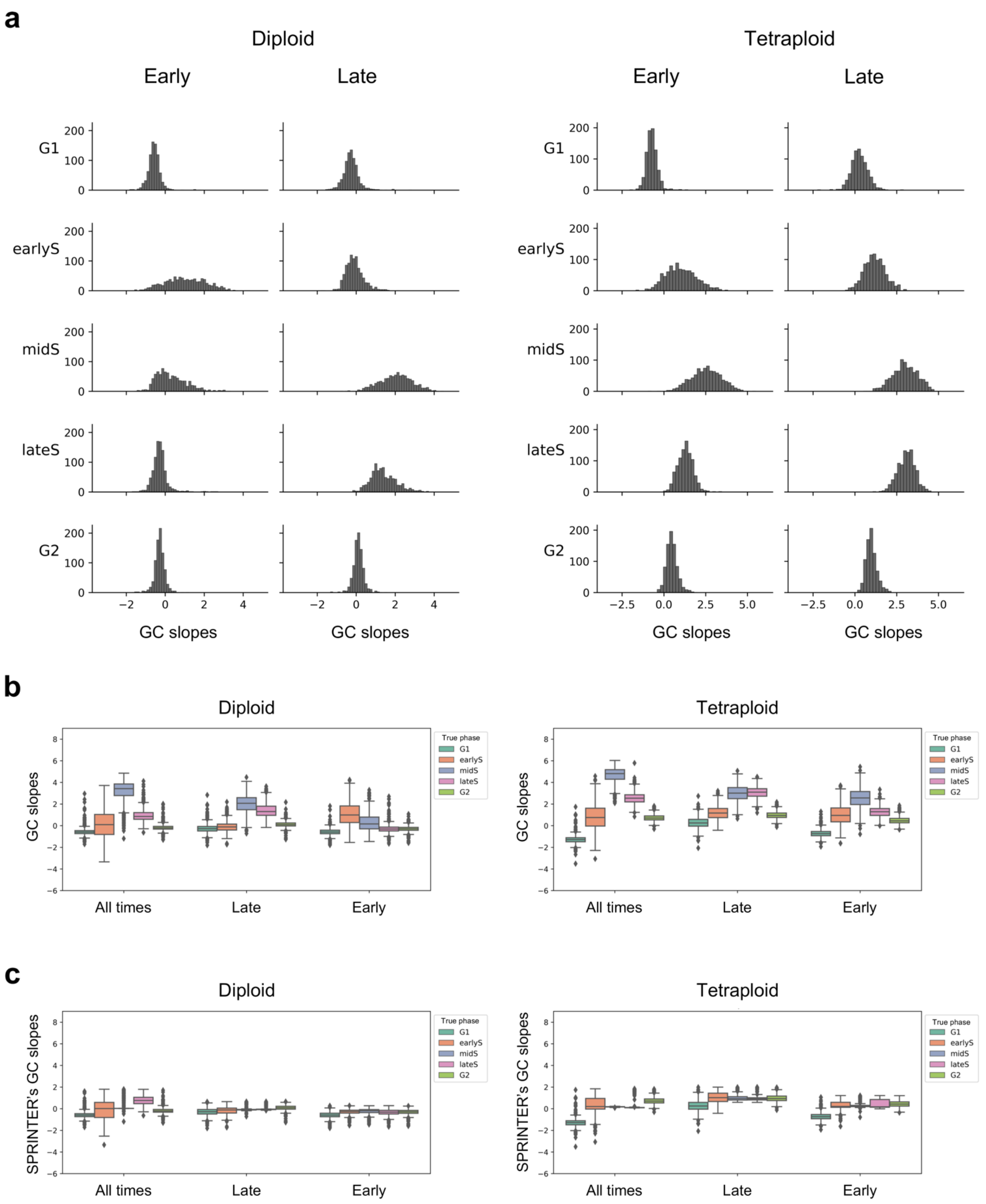

Supplementary Fig. 6: SPRINTER infers similar GC corrections across all cells in different cell cycle phases. a, Count (y-axis) of raw GC slopes (i.e., slope of the inferred linear relationship between GC content and read counts before outlier correction, x-axis) inferred by SPRINTER for GC correction in early and late replicating bins (columns) for all ground truth diploid (left) and tetraploid (right) cells across all five cell cycle phases (rows). **b,** Before correction of outliers, raw SPRINTER GC slopes are computed for early replicating, late replicating, and all bins across all 4,410 diploid (left) or 4,434 tetraploid (right) cells in different cell cycle phases (x-axis). **c,** Final SPRINTER GC slopes are computed after correction of outliers for early replicating, late replicating, and all bins across all 4,410 diploid (left) or 4,434 tetraploid (right) cells in different cell cycle phases (x-axis). The final SPRINTER GC slopes are similar across all cells, which is expected since GC bias should only be slightly impacted by different cell cycle states. In all panels, box plots show the median and the interquartile range (IQR), and the whiskers denote the lowest and highest values within 1.5 times the IQR from the first and third quartiles, respectively.

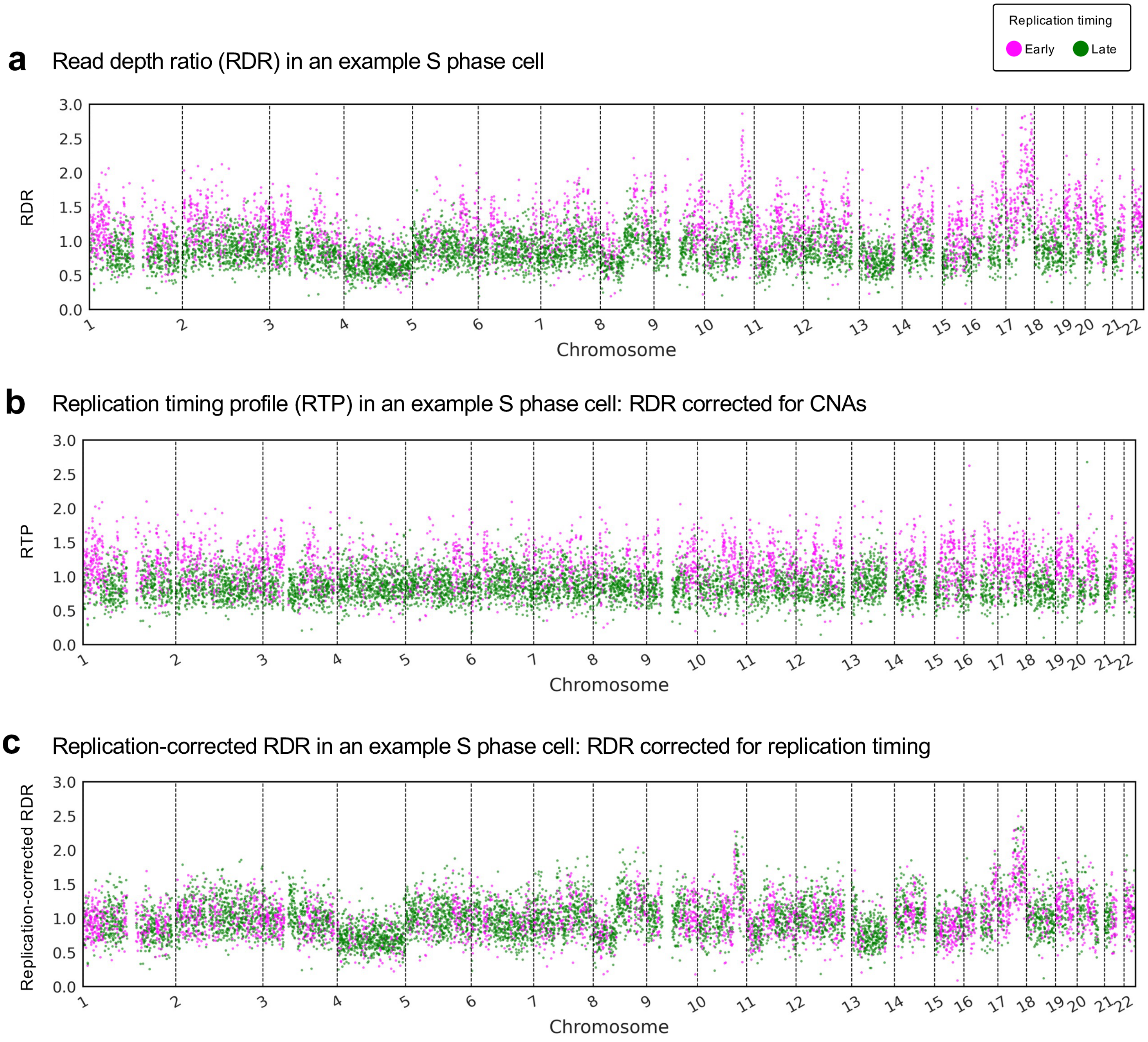

Supplementary Fig. 7: SPRINTER's replication-aware framework enables the differentiation of RDR changes due to replication fluctuations and CNAs. **a,** Changes in read counts for 250kb genomic bins across autosomes (x-axis) with either early (magenta) or late (green) replication timing were measured using RDRs (y-axis, the fraction between observed vs. expected read counts) for a tetraploid HCT116 cell in mid S phase. **b,** A replication timing profile (y-axis) was computed for the same cell by correcting RDRs for CNAs based on the copy-number segments inferred by SPRINTER, preserving clear fluctuations between bins with different replication timing (with magenta early regions having higher RDRs than green late regions on average). **c,** Replication-corrected RDRs (y-axis) were computed for the same cell by correcting RDRs for replication fluctuations, such that the remaining RDR changes are likely due to CNAs and are not influenced by replication (in each segment there is no clear difference between bins with different replication timing).

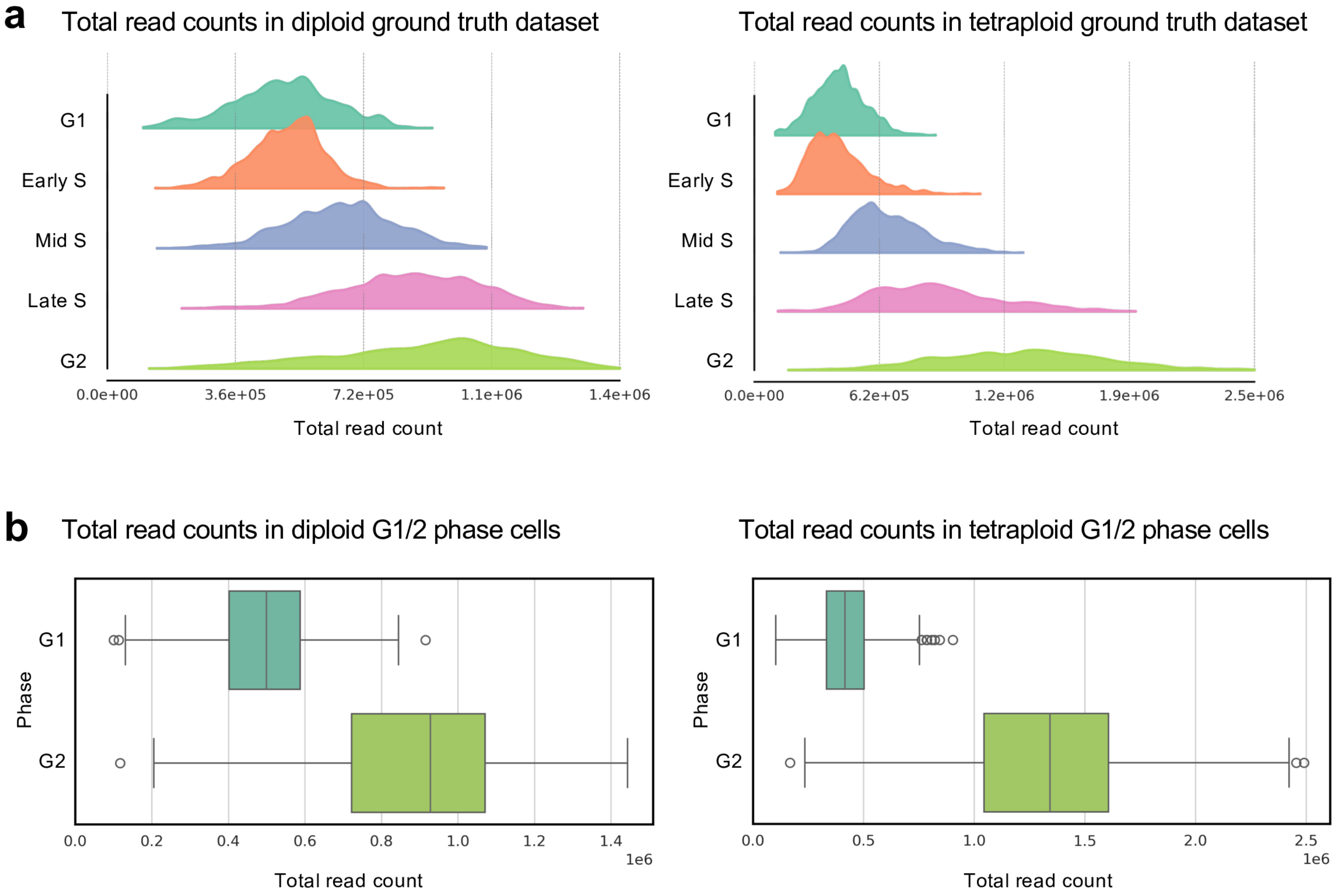

Supplementary Fig. 8: Cells in different cell cycle phases yield increasing total read counts. a, A kernel density estimate represents the distribution of total read counts (x-axis) computed for all cells in different cell cycle states (y-axis) in either the diploid (left) or tetraploid (right) ground truth datasets. **b**, Box plots show the total read counts (x-axis) of all G1 and G2 phase cells (y-axis) in either the diploid (left, with 1,650 G1/2 cells) or tetraploid (right, with 1,595 G1/G2 cells) ground truth datasets, with the median represented by the middle line, the interquartile range (IQR) by the box, and whiskers representing the lowest and highest values within 1.5 times the IQR from the first and third quartiles, respectively. As expected, G2 cells yield higher total read counts than G1 cells.

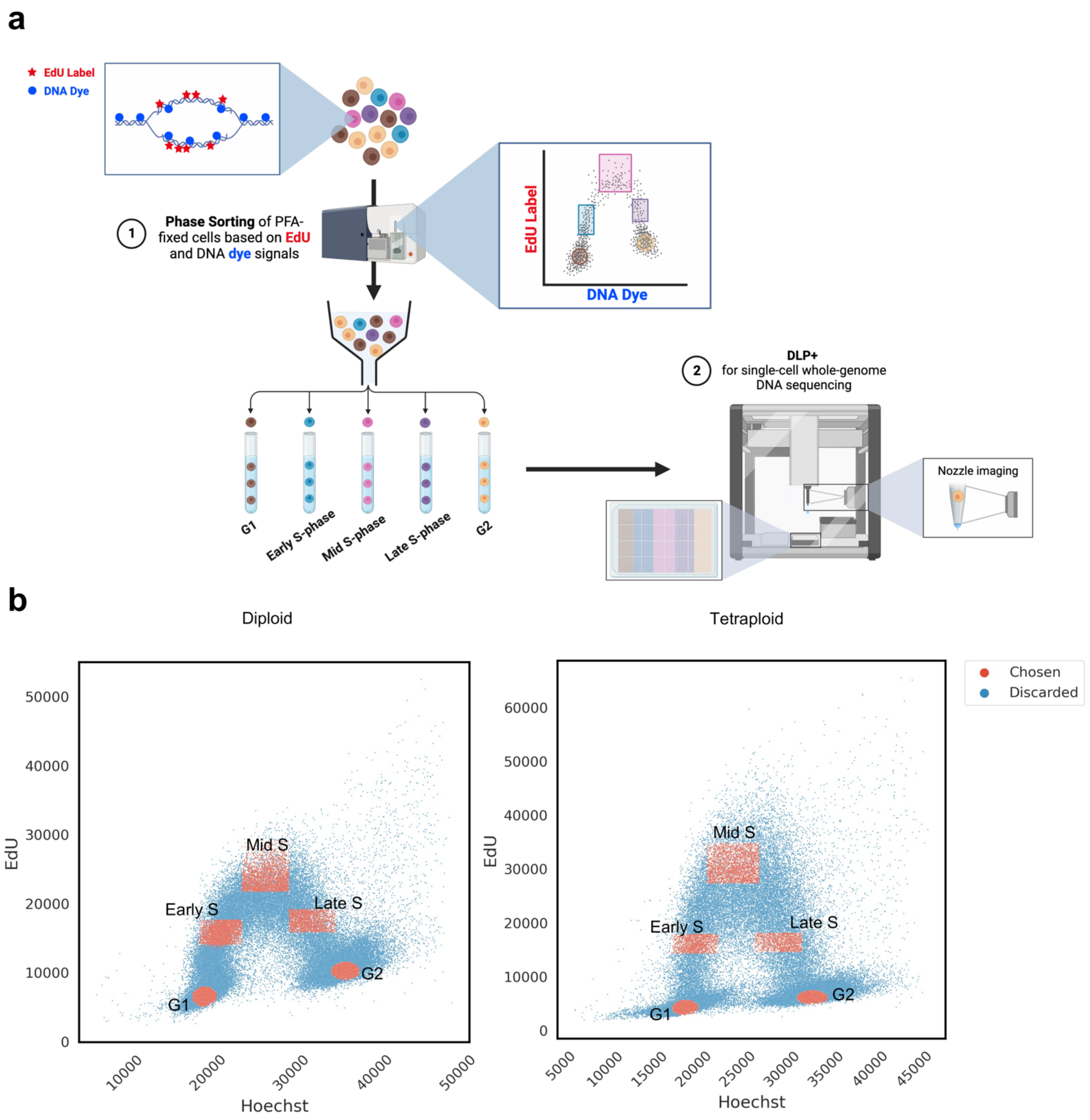

Supplementary Fig. 9: Generation of an accurate ground truth dataset of cells sorted into different cell cycle phases using EdU and Hoechst labelling. **a,** The experimental design for generating an accurate ground truth dataset. (Top left) Cells were treated with two separate dyes, EdU (which is actively incorporated into replicating DNA, red stars) and Hoechst 33342 (which binds all DNA, blue circles). (Middle left) Cells were then FACS-sorted based on signals from both EdU and Hoechst into (bottom left) five different cell cycle phases: G1, early S, mid S, late S, and G2. (Bottom right) Cells in each cell cycle phase were then single-cell whole-genome DNA sequenced using DLP+, which also captures nozzle images of each sequenced cell nucleus. **b,** The EdU (y-axis, Alexa Fluor 647, excited with a 642nm laser and emission collected in a 670/30BP filter) and Hoechst (x-axis, excited using a 405nm laser and emission collected in a 460/50BP filter) dye signals were measured to FACS sort all cells (dots) from HCT116 diploid (left) and tetraploid (right) cell lines, with cells chosen in each cell cycle phase for inclusion in the ground truth dataset (red) or discarded (blue).

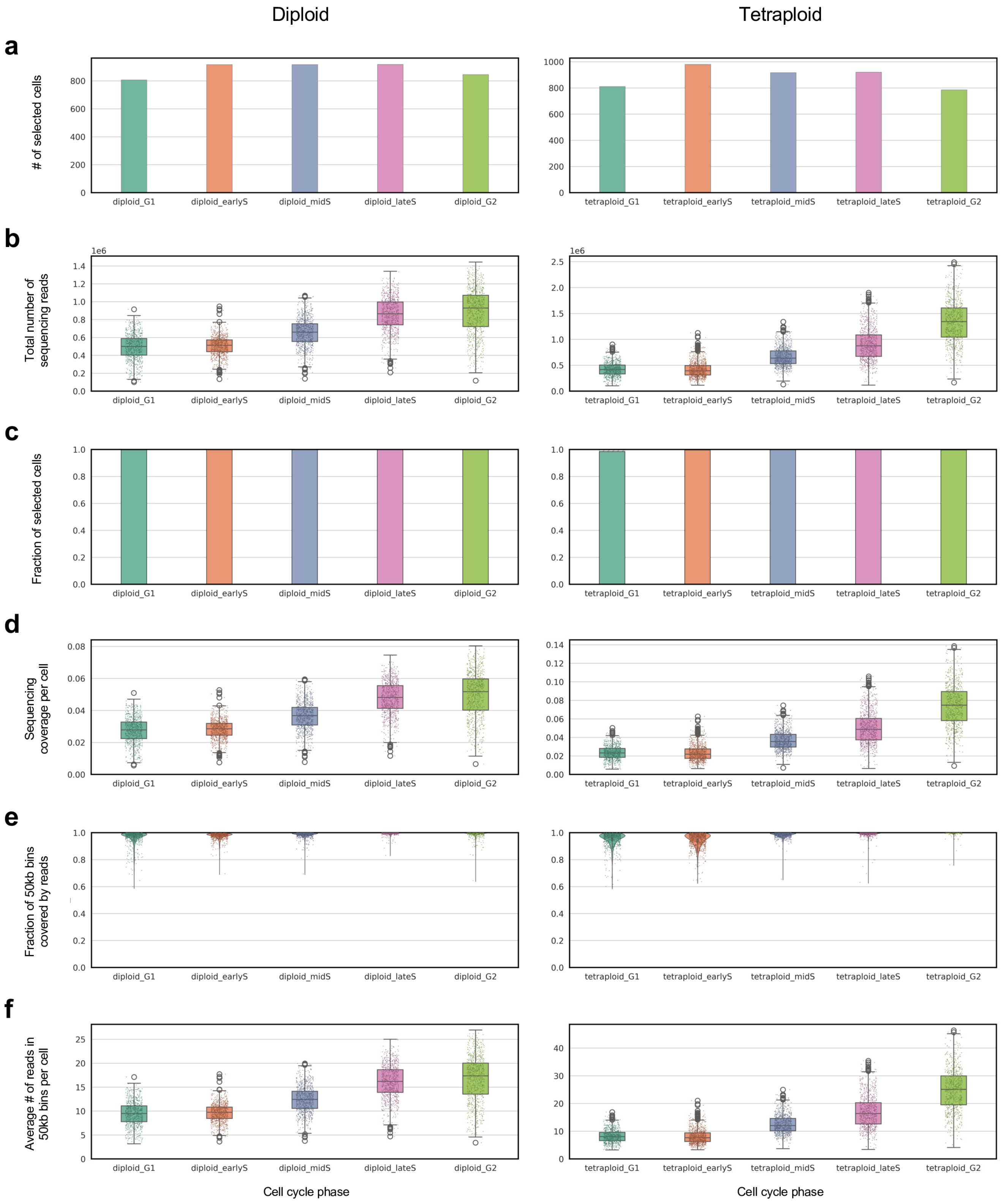

Supplementary Fig. 10: scDNA-seq features of the generated ground truth datasets. Across cells from five cell cycle phases (x-axis) which were single-cell DLP+ sequenced from the diploid (left) and tetraploid (right) ground truth datasets, multiple sequencing features have been computed: (**a**) number of cells selected for analysis, (**b**) total number of sequenced reads, (**c**) fraction of selected cells with a sufficient number of sequencing reads (>100k) indicating successful DNA library preparation among all cells (threshold on yielded sequencing reads to define total cell count is estimated as the median of the 2nd percentile computed across all samples for all isolated objects with above average intensity, measured by nuclear imaging), (**d**) sequencing coverage per cell, (**e**) fraction of 50kb genomic bins covered by at least one sequencing read, and (**f**) average number of sequencing reads in 50kb genomic bins per cell. In all panels, box plots show the median and the interquartile range (IQR), and the whiskers denote the lowest and highest values within 1.5 times the IQR from the first and third quartiles, respectively.

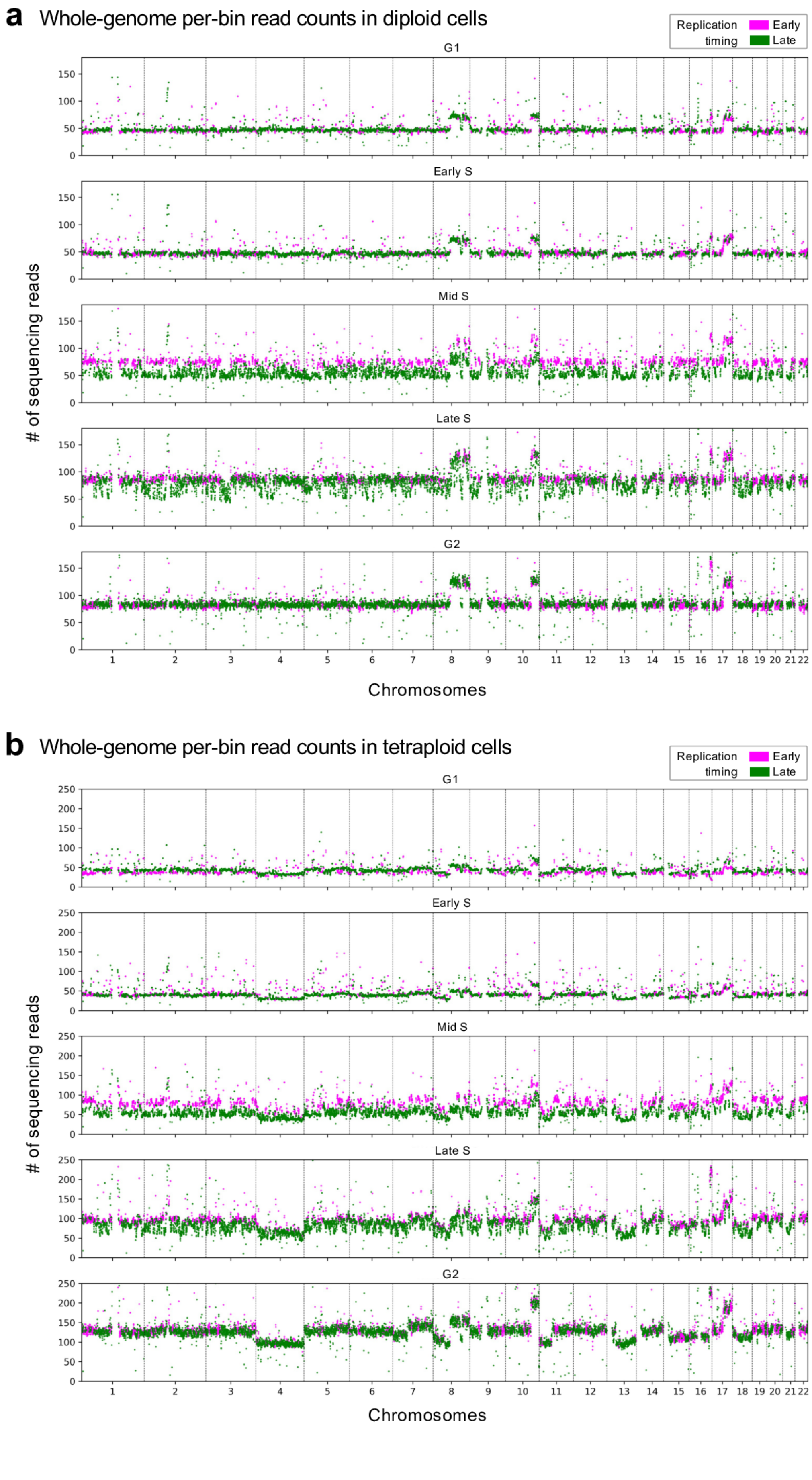

Supplementary Fig. 11: Read counts in the ground truth datasets generated with 4,410 diploid and 4,434 tetraploid cells are compatible with expected replication fluctuations. The median read count (y-axis) was calculated for 250kb genomic bins with either early (magenta) or late (green) replication timing along autosomes in the genome (x-axis) for all cells in the (**a**) diploid and (**b**) tetraploid ground truth datasets in different cell cycle phases (rows).

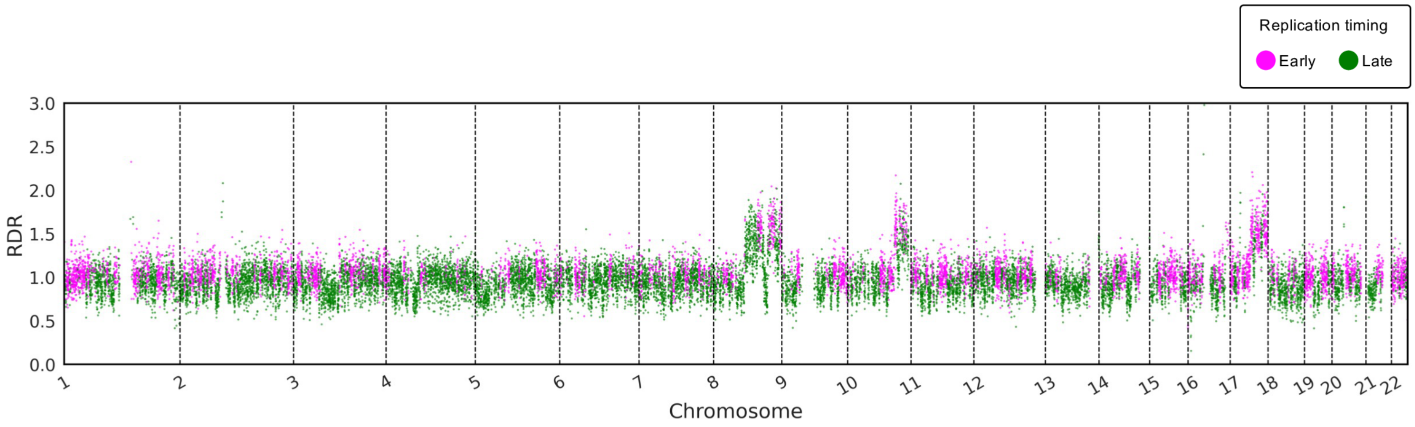

Supplementary Fig. 12: SPRINTER's erroneous S phase calls in G1 phase cells in the diploid ground truth are compatible with minor infiltrating errors. Average RDR (y-axis) was computed for each 250kb genomic bin across the genome (x-axis) in the diploid ground truth G1 phase cells that were called as S phase by SPRINTER, showing that early bins (magenta) have consistently higher RDRs than late bins (green), as expected for actively replicating cells in S phase.

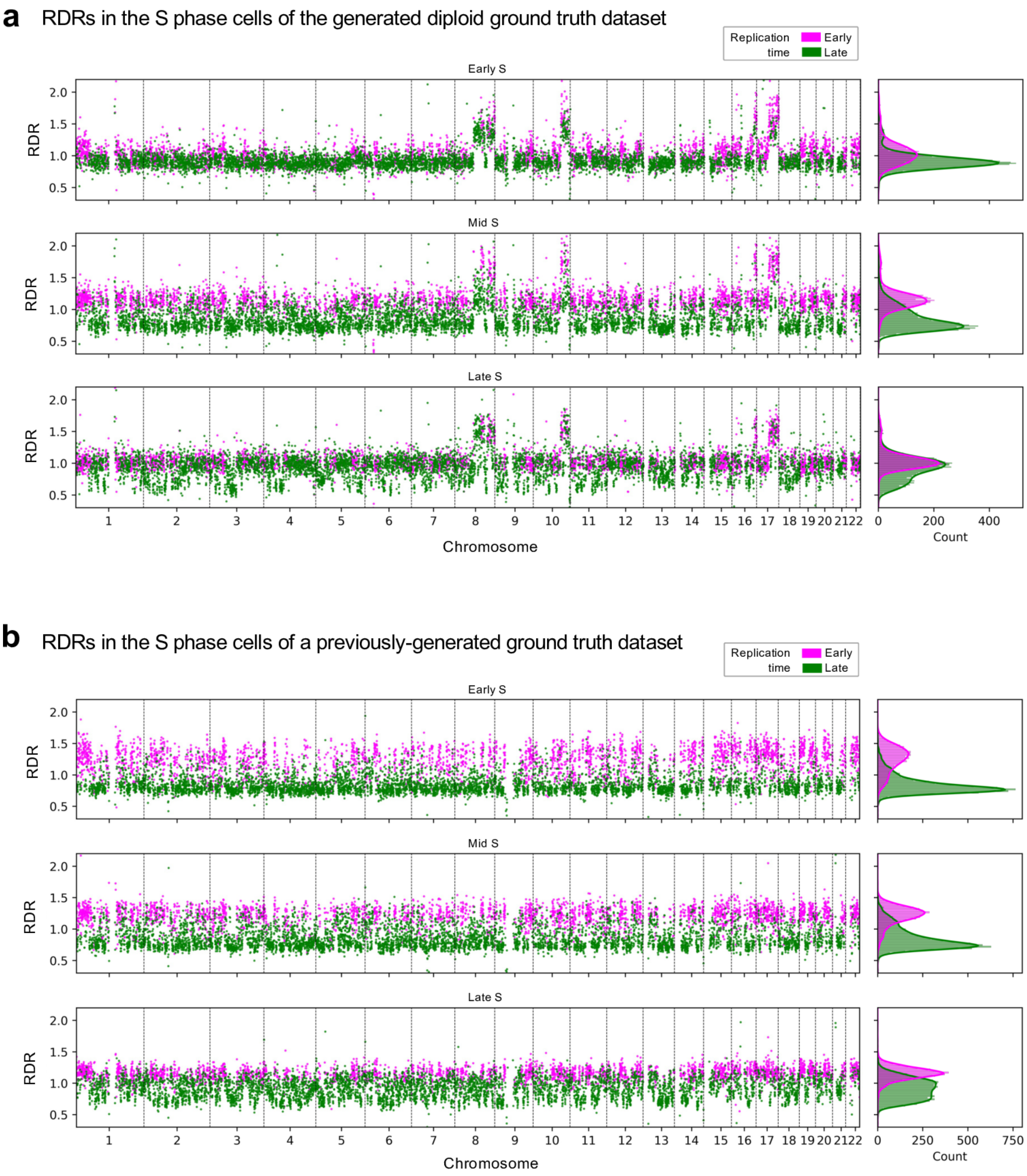

Supplementary Fig. 13: The novel ground truth dataset captures cells at different stages of S phase in contrast to previous datasets. a, The average RDR (y-axis) was measured in 250kb bins with either early (magenta) or late (green) replication timing across autosomes in the genome (x-axis) for all cells classified as early S phase (top), mid S phase (middle), and late S phase (bottom) in the novel diploid ground truth dataset. As expected, cells at different stages of S phase exhibit clearly different replication fluctuations in RDRs: in early S phase only early replicating bins shift to higher values of RDR, in mid S phase all the early bins have completed replication and have distinctly higher values of RDR than late bins, and in late S phase late bins also start replication and some of these bins have increased RDR values. **b,** The average RDR (y-axis) was measured in 250kb bins with either early (magenta) or late (green) replication timing across autosomes in the genome (x-axis) for all cells classified as early S phase (top), mid S phase (middle), and late S phase (bottom) in a previous dataset of 5,970 lymphoblastoid cells based on standard FACS sorting of replicating cells. In contrast to (**a**), cells at different stages of S phase do not exhibit clearly different replication fluctuations of RDRs, suggesting that cells in early and late S phase have not been comprehensively captured, as reported before in the previous study of this dataset.

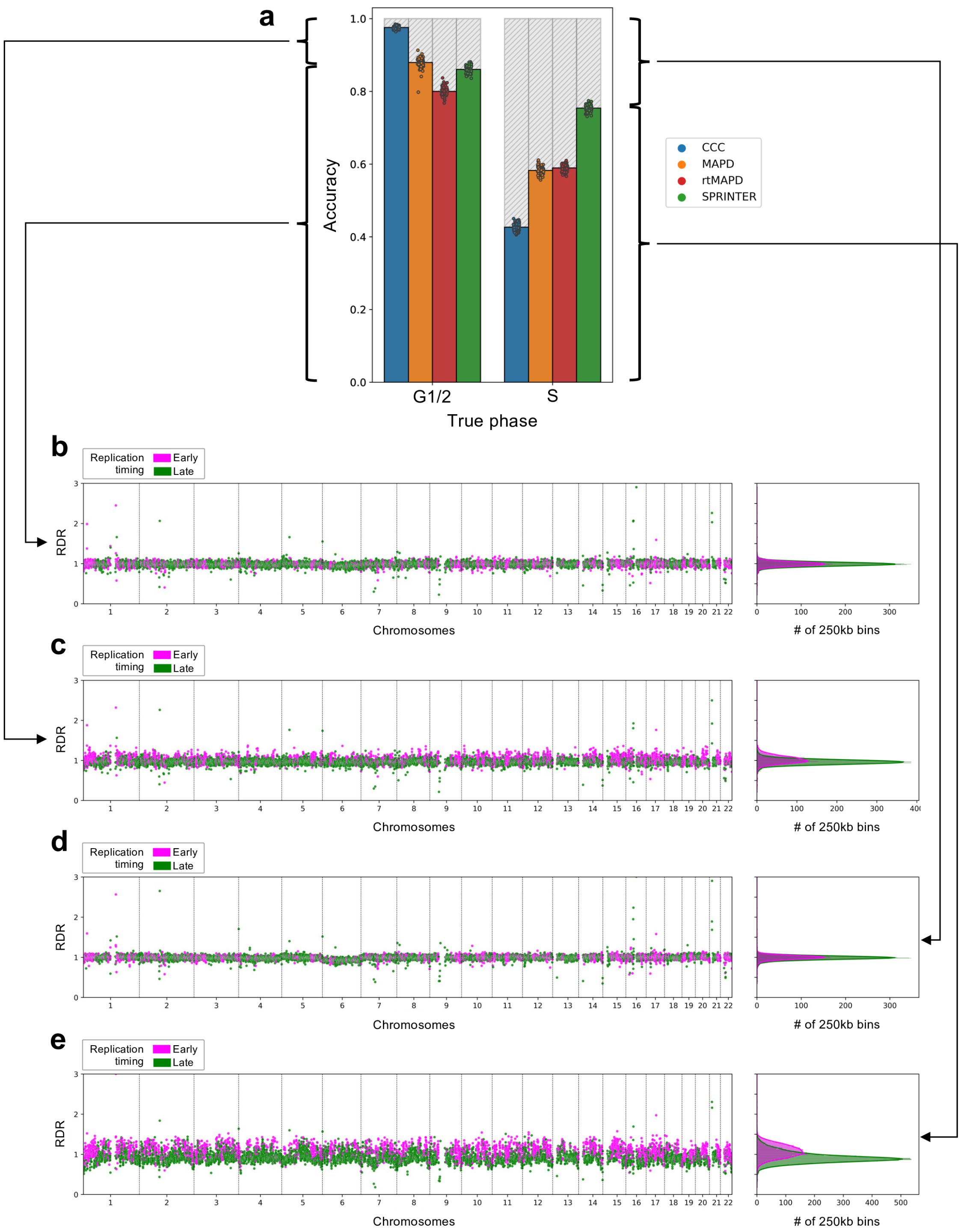

Supplementary Fig. 14: SPRINTER exhibits higher accuracy and sensitivity in the identification of S phase cells than existing methods in a previously published 10X CNV Solution dataset. a, The proportion of correctly identified G1/2 and S phase cells computed for SPRINTER and existing approaches (CCC, MAPD, and rtMAPD, which extends MAPD with replication timing) for 100 subpopulations of cells (each dot). Each subpopulation was formed by randomly sampling 500 cells from a previous ground truth dataset of 5,970 cells from the lymphoblastoid cell line GM12878 sorted with standard FACS and sequenced using the 10x CNV Solution. **b-c,** The average RDR (y-axis) was measured in 250kb bins with either early (magenta) or late (green) replication timing across autosomes in the genome (x-axis) for all G1/2 cells in the previously published ground truth dataset that were inferred as either (**b**) G1/2 phase or (**c**) S phase by SPRINTER. In contrast to cells in (**b**) that were inferred as G1/2 cells by SPRINTER, the remaining cells that were inferred as S phase by SPRINTER and also MAPD and rtMAPD in (**c**) exhibit clear replication fluctuations across the genome, indicating that these cells are the result of known FACS infiltrating errors in the classification of cell cycle phases in the previous dataset and likely correspond to true S phase cells, in line with SPRINTER's results and previous reports in the same study. **d-e,** The average RDR (y-axis) was measured in 250kb bins with either early (magenta) or late (green) replication timing across autosomes in the genome (x-axis) for all S phase cells in the previous ground truth dataset that were inferred as either (**d**) G1/2 phase or (**e**) S phase by SPRINTER. In contrast to cells in (**e**) that were inferred as S phase cells by SPRINTER, the remaining cells in (**d**) that were inferred as G1/2 phase by SPRINTER do not exhibit clear replication fluctuations, similar to other G1/2 cells in the dataset in (**b**), indicating that these cells are the result of known FACS infiltrating errors in the classification of cell cycle phases in the previous dataset and likely correspond to true G1/2 phase cells, in accordance with SPRINTER's results.

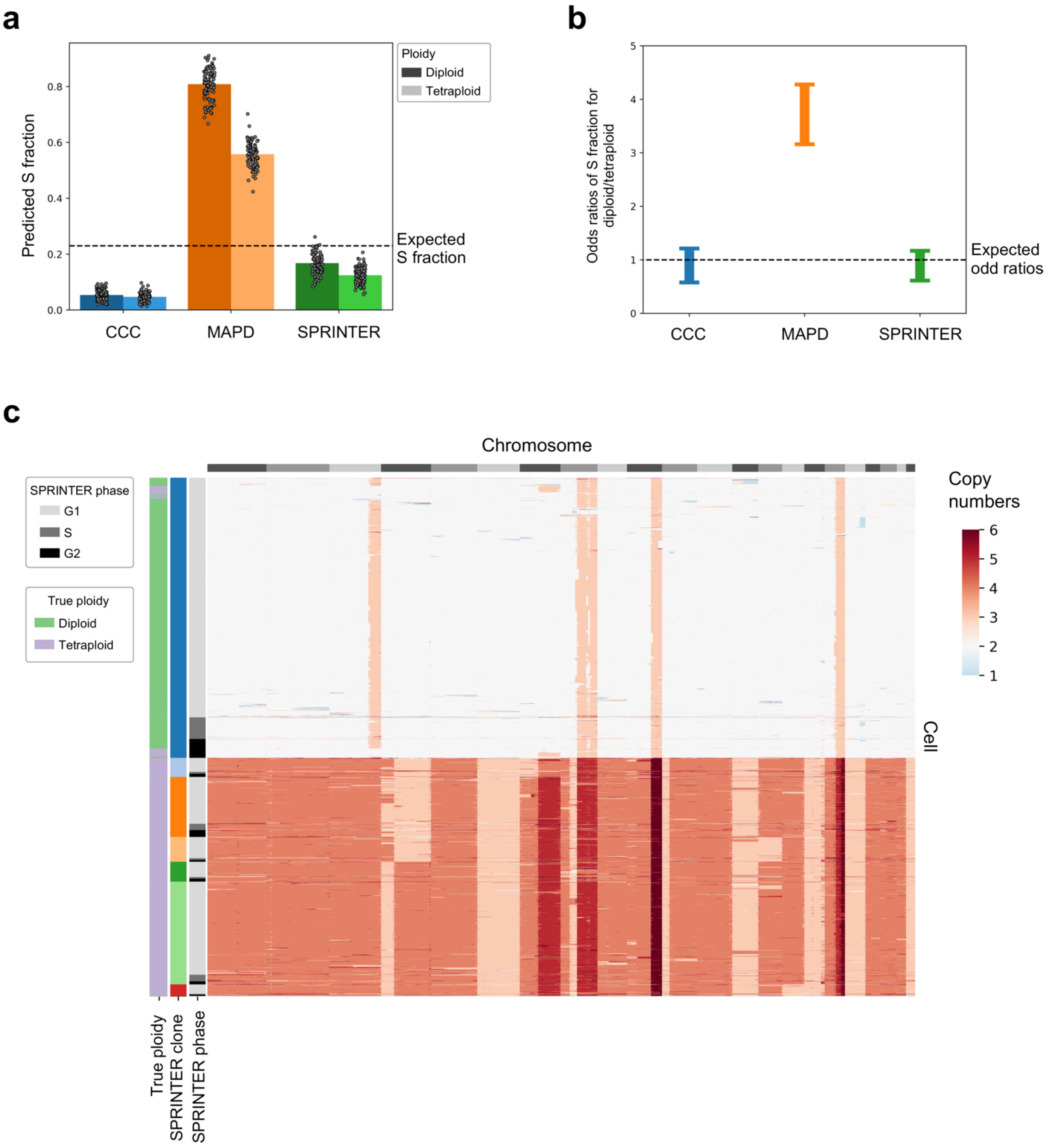

Supplementary Fig. 15: SPRINTER exhibits high accuracy and sensitivity in identifying S phase cells from mixed subpopulations of diploid and tetraploid cells in contrast to existing methods. a, Predicted S fractions (y-axis) in an additional ground truth dataset comprising 4,163 diploid (dark) and tetraploid (light) HCT116 cells sequenced together as a mixed subpopulation were computed using SPRINTER (green) and existing methods CCC (blue) and MAPD (orange). The expected S fraction (dashed line) was calculated as the fraction of cells with values of EdU vs Hoechst dye signals clearly different to those of G1/2 cells. **b,** 95% confidence interval of the odds ratios of the S fraction in a total of 4,163 diploid vs tetraploid cells sequenced together (vertical lines, y-axis) were calculated based on the S fraction inferred from the mixed subpopulation by SPRINTER (green) and existing methods, CCC (blue) and MAPD (orange). The expected odds ratio is 1, since the expected fraction of S phase cells in diploid and tetraploid cells is not significantly different based on expectations obtained from the values of EdU vs Hoechst signals. **c,** Baseline copy numbers (heatmap colours) were inferred by SPRINTER on 4,163 mixed diploid (light green) and tetraploid (lilac) cells (rows) across ~3Mb genomic bins (columns). SPRINTER inferred clones (middle colour bar) and assigned S phase and G2 cells to each clone (third colour column) in this dataset.

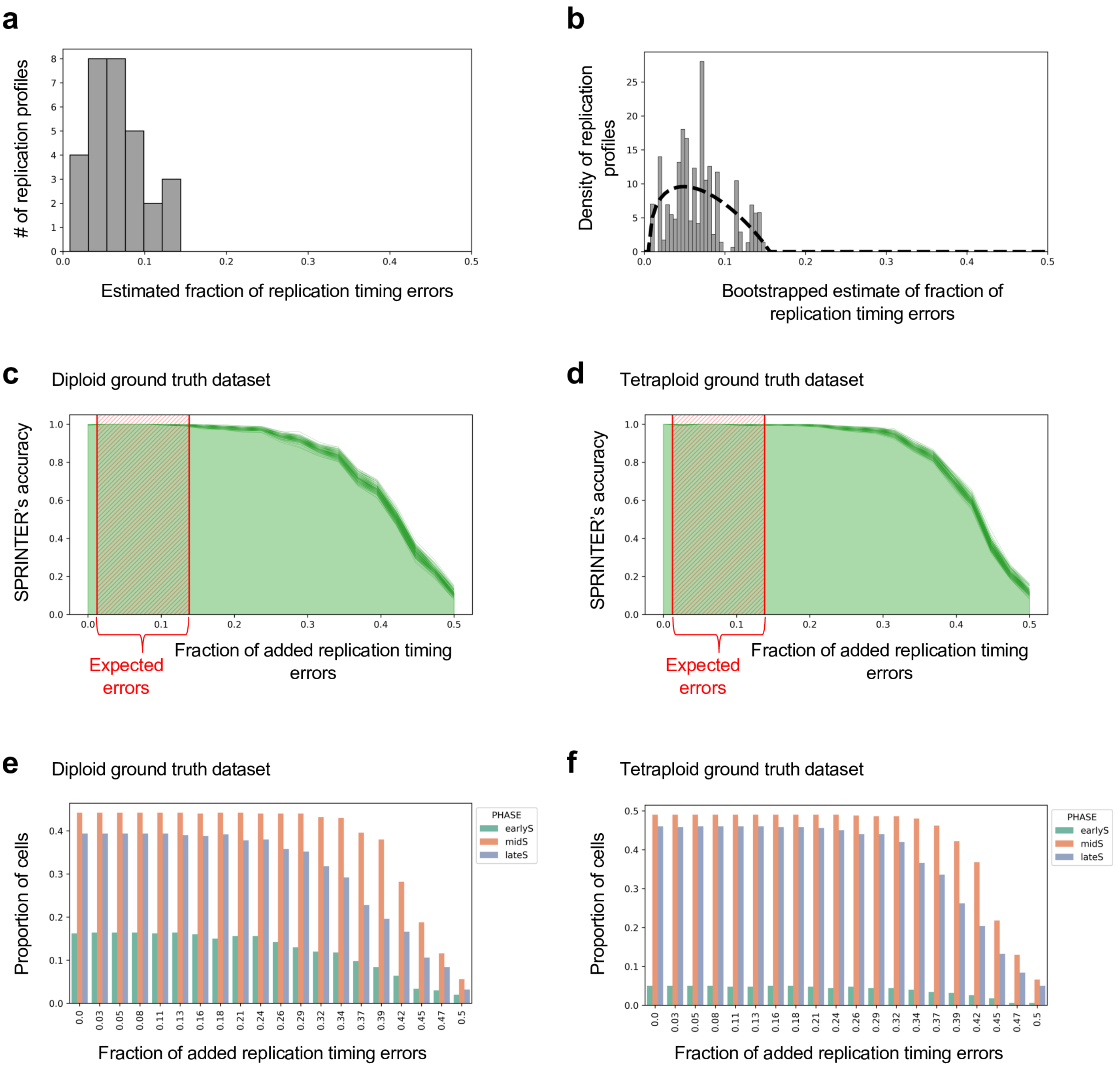

Supplementary Fig. 16: SPRINTER is robust to a higher fraction of replication-timing errors or alterations than the maximum expected in different normal and cancer cells. a, The expected fraction of replication timing errors (x-axis) was estimated for each available replication profile (y-axis) by quantifying the fraction of the genome with a classification of early/late replication timing that differed from SPRINTER's reference replication timing classifications used by default. **b,** The expected fraction of replication timing errors (x-axis) was computed with the same method used in (**a**) but using 20 bootstrapped repeats for each profile, such that in every repeat the genomic regions used in the comparison were randomly sampled with replacement. A beta distribution (dotted line) was fitted to the bootstrapped values to estimate the density of the distribution. **c-d,** The proportion of S phase cells that are correctly identified by SPRINTER (y-axis) after introducing a varying fraction of errors in the replication timing classifications (x-axis) was calculated using 500 S phase cells randomly sampled from either the diploid (**c**) or tetraploid (**d**) ground truth dataset for 100 bootstrapped repeats (lines). The 95% confidence interval of the expected fraction of errors as calculated in (**b**) is shown (red box). **e-f,** For each experiment in (**c**) and (**d**), the proportions of S phase cells that are correctly identified by SPRINTER (y-axis) after introducing a varying fraction of errors in the replication timing classifications (x-axis) are separated by cell cycle phase for diploid (**e**) and tetraploid (**f**) cases, respectively.

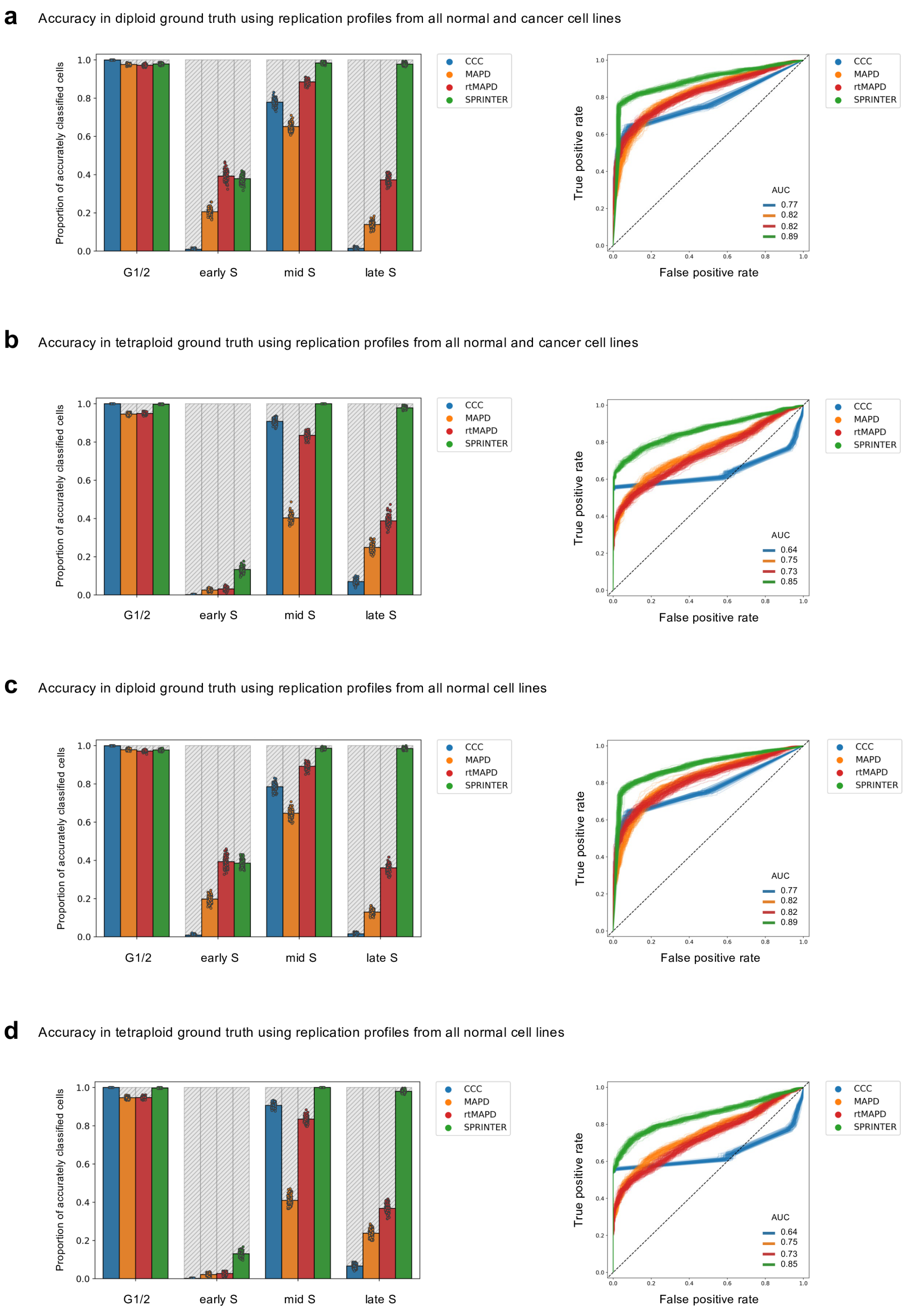

Supplementary Fig. 17: SPRINTER preserves high accuracy and sensitivity in identifying replicating cells when using different input replication profiles. (Left panels) The proportion of correctly identified G1/2 and S phase cells (y-axis) was computed for SPRINTER (green) and three previous methods (CCC, MAPD, and rtMAPD, which extends MAPD with replication timing) across four cell cycle phases (x-axis) for 100 subpopulations of cells (each dot), each of which was formed by randomly sampling 500 cells from a total of either (**a** and **c**) 4,410 diploid or (**b** and **d**) 4,434 tetraploid HCT116 cells. SPRINTER has been executed using the input replication profiles from either (**a** and **b**) all normal and cancer cell lines or (**c** and **d**) from all normal cell lines. (Right panels) For all the corresponding datasets and results, ROC curves (false positive vs. true positive rate) were calculated to measure performance in distinguishing G1 cells from actively replicating cells by using the classification scores computed by existing methods (blue, orange, and red) or combining SPRINTER's S and G2 p-values (using the minimum in green).

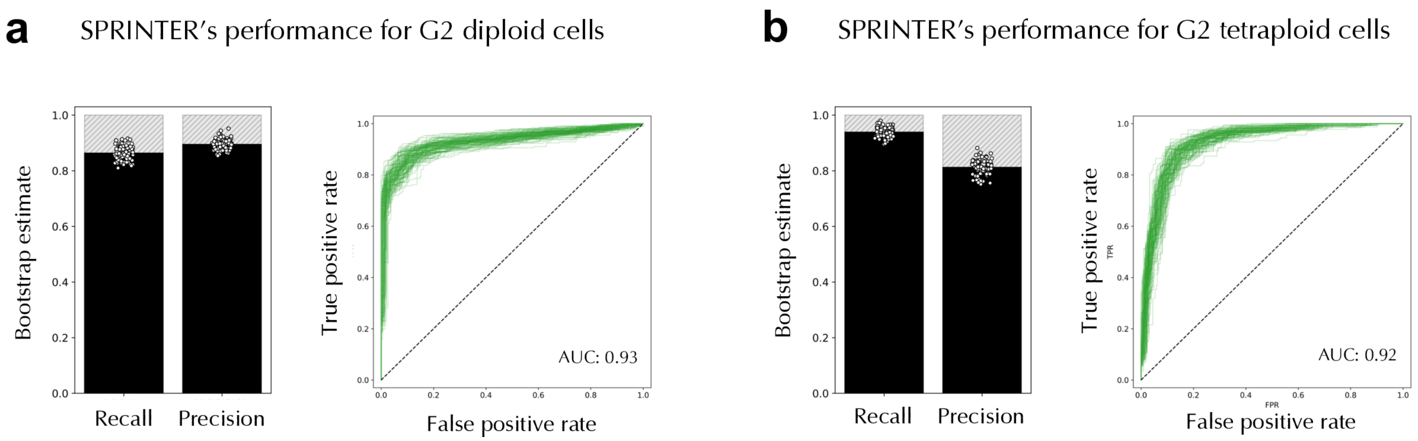

Supplementary Fig. 18: SPRINTER accurately infers G2 phase cells in both the diploid and tetraploid ground truth datasets. (Left plots) The precision and recall for G2 phase cells were computed by bootstrapping for 100 repeats the G1/G2 phase cells identified by SPRINTER from a total of either (**a**) 4,410 diploid or (**b**) 4,434 tetraploid HCT116 cells. (Right plots) ROC curves were calculated to measure the performance of SPRINTER in distinguishing G1 vs G2 phase cells for either the diploid or tetraploid cells analysed in (**a**) or (**b**), respectively, by using the G2 p-values computed by SPRINTER.

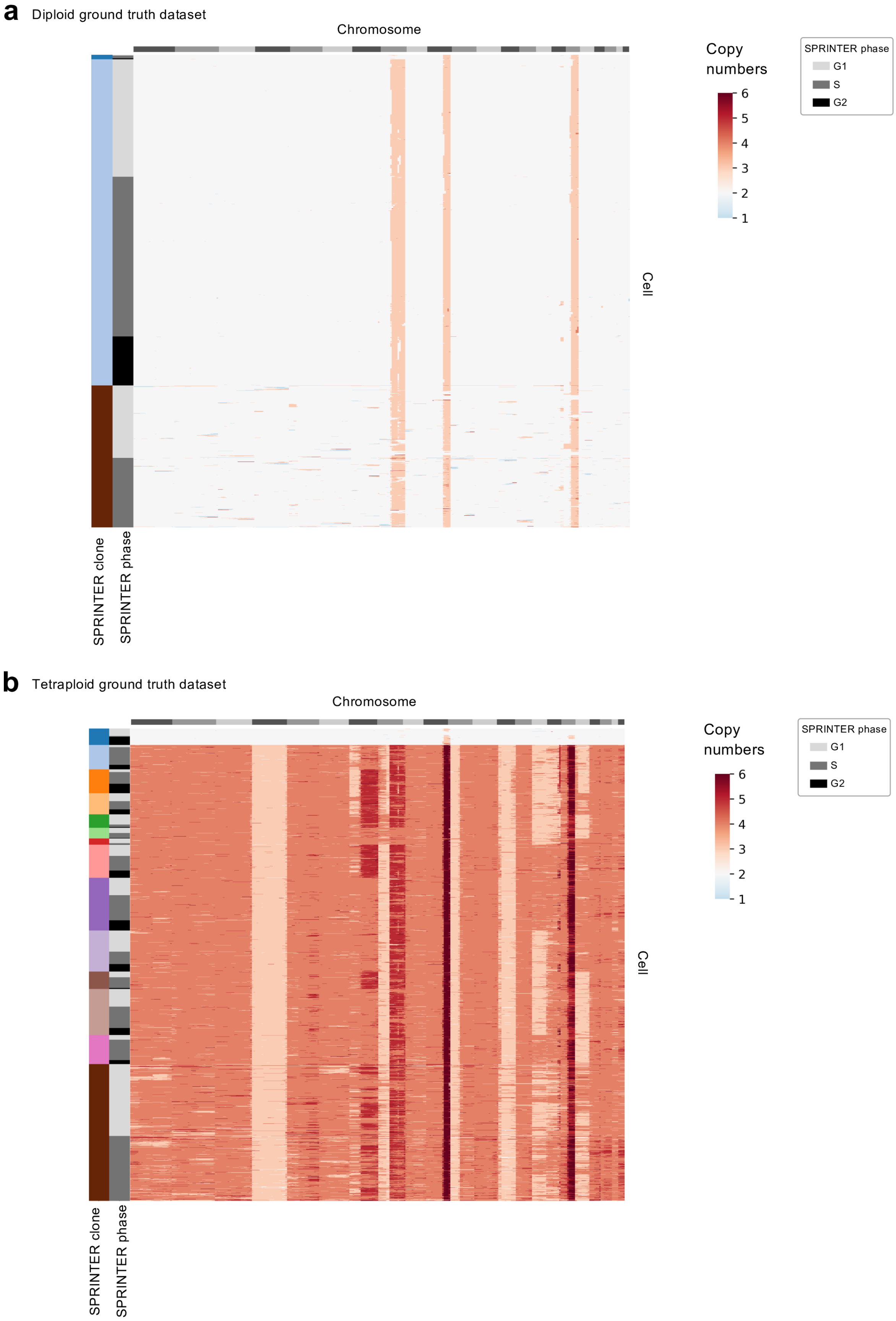

Supplementary Fig. 19: SPRINTER results for 4,410 diploid and 4,434 tetraploid ground truth cells. Baseline copy numbers (heatmap colours) were inferred by SPRINTER on (**a**) 4,410 diploid and (**b**) 4,434 tetraploid ground truth cells across ~3Mb genomic bins (columns). SPRINTER inferred clones (left-side colours) and assigned S phase and G2 cells to each clone (light grey for G1 phase, dark grey for S phase, and black for G2 phase), with multiple distinct clones identified in the tetraploid but not the diploid ground truth dataset (the dark brown bar denotes noisy cells excluded from any clone).

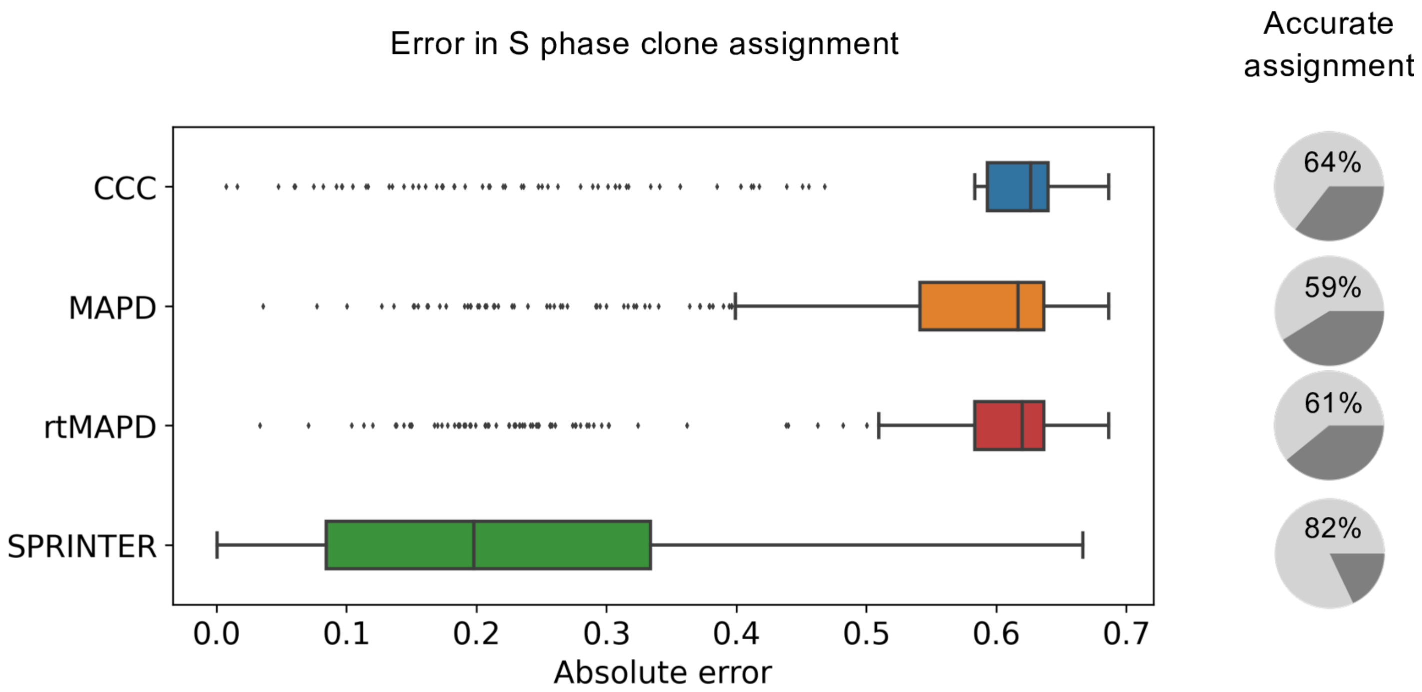

Supplementary Fig. 20: SPRINTER accurately assigns S phase cells to clones in contrast to previous approaches. The absolute error rate (x-axis) between the fraction of inferred S phase cells assigned to a clone by each method (y-axis) and the expected fraction was calculated per cell in 30 populations of 300 tetraploid cells each, altogether comprising 389 clones using all methods (colours). The proportion of clones for which the assigned true S fraction was compatible with the expected S fraction was computed using a Binomial test (Pie charts). In all panels, box plots show the median and interquartile range (IQR) with whiskers denoting values within 1.5 times the IQR from the first and third quartiles. Box plots show the median and the interquartile range (IQR) with whiskers denoting values within 1.5 times the IQR from the first and third quartiles.

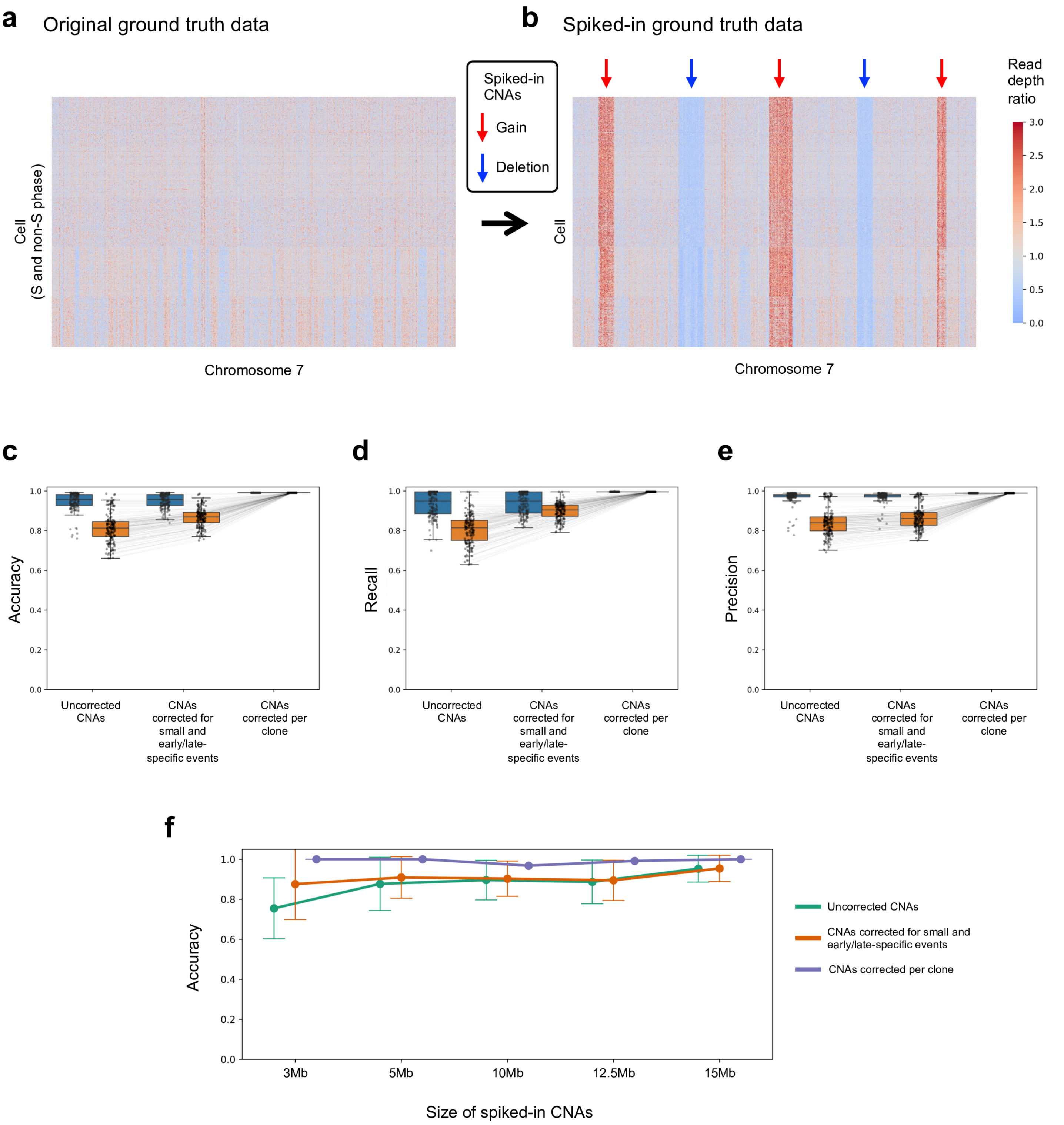

Supplementary Fig. 21: Spike-in experiment of CNAs demonstrates that SPRINTER accurately recovers most >3Mb copy-number events in both S and non-S phase cells. a-b, Copy-number gains (red arrows) and losses (blue arrows) of varying size have been spiked into diploid chromosomes in the cells from the diploid ground truth dataset by scaling the original read counts (**a**) by different scaling factors (2 and 1/2, respectively) to obtain spike-in datasets with known events (**b**). **c-e,** Accuracy (y-axis in **c**), recall (y-axis in **d**), and precision (y-axis in **e**) of SPRINTER have been measured per cell (each dot) when applied to 500 non-S (blue) and S (orange) phase cells with 55,000 spike-in events (110 events per cell) when considering the raw, uncorrected CNAs inferred directly by SPRINTER in each cell (left), the corrected CNAs inferred by SPRINTER (middle, including correction of replication-timing-specific CNAs in S phase cells and small CNAs), and the CNAs inferred by SPRINTER at the clone-level for each cell (right). Box plots show the median and the interquartile range (IQR), and the whiskers denote the lowest and highest values within 1.5 times the IQR from the first and third quartiles, respectively. **f,** The accuracy (y-axis) for the same CNAs inferred by SPRINTER with different corrections (colours) is measured for events of different sizes (x-axis) in the same cell populations with spike-in events as defined in (c-e), with error bars representing the 95% confidence interval.

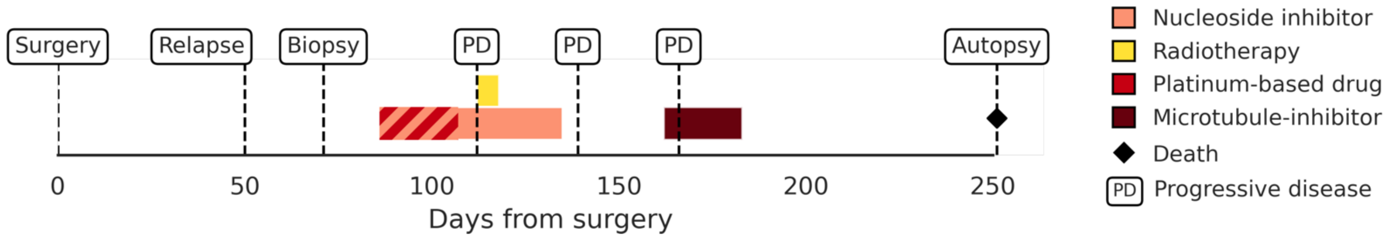

Supplementary Fig. 22: Clinical history of patient CRUKP9145 who participated in the TRACERx study and PEACE programme. Timeline of key clinical events including surgery, relapse, progressive disease (PD), treatments, and autopsy from surgery to death.

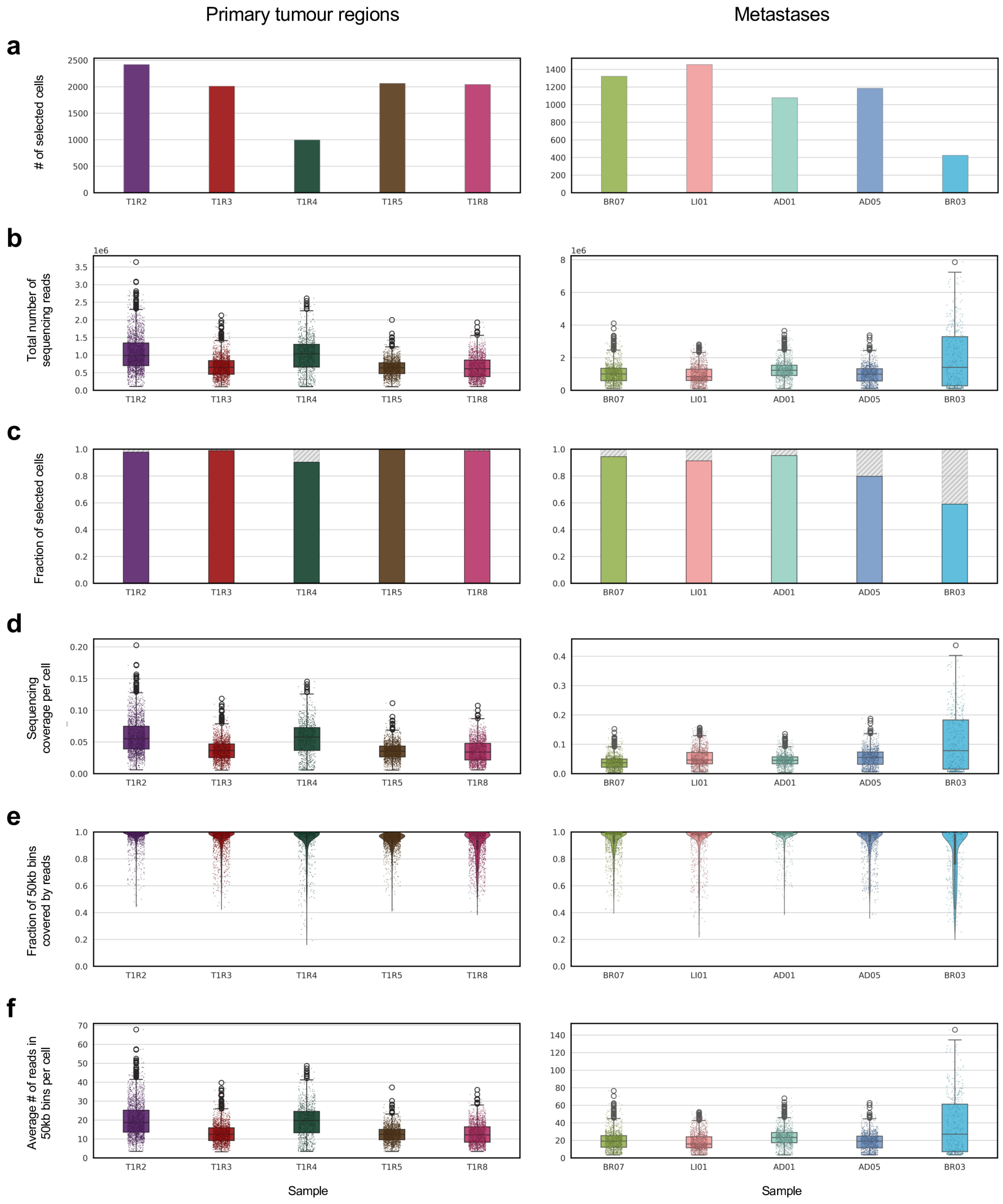

Supplementary Fig. 23: scDNA-seq features of the novel NSCLC dataset. Across 10 samples (x-axis) single-cell DLP+ sequenced from five primary tumour regions (left) and five metastases (right) of CRUKP9145, multiple sequencing features have been computed: (**a**) number of cells selected for analysis, (**b**) total number of sequenced reads, (**c**) fraction of selected cells with a sufficient number of sequencing reads (>100k) indicating successful DNA library preparation among all cells (threshold on yielded sequencing reads to define total cell count is estimated as the median of the 2nd percentile computed across all samples for all isolated objects with above average intensity, measured by nuclear imaging), (**d**) sequencing coverage per cell, (**e**) fraction of 50kb genomic bins covered by at least one sequencing read, and (**f**) average number of sequencing reads in 50kb genomic bins per cell. In all panels, box plots show the median and the interquartile range (IQR), and the whiskers denote the lowest and highest values within 1.5 times the IQR from the first and third quartiles, respectively.

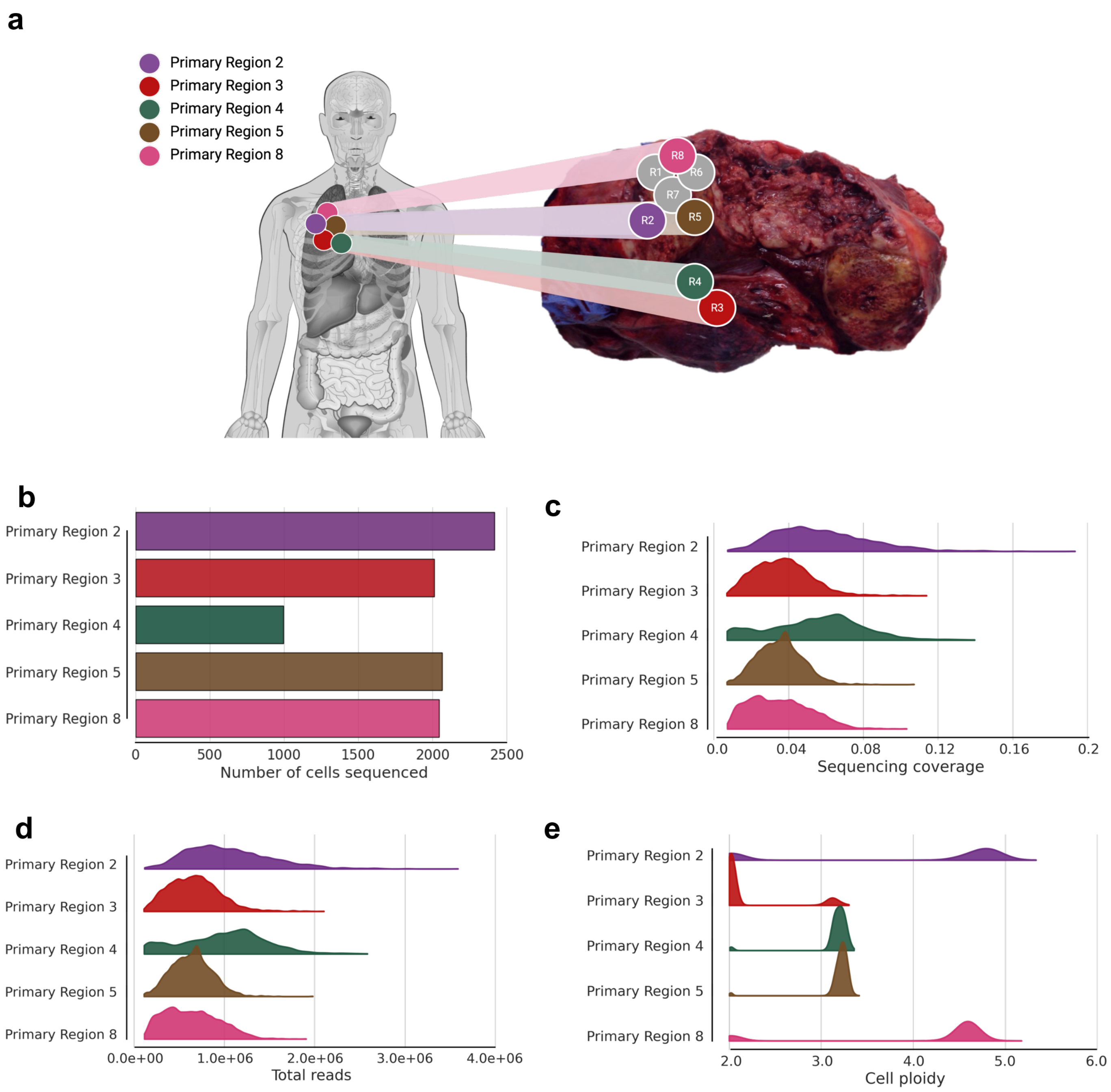

Supplementary Fig. 24: Sequencing details of all single cells sequenced from 5 primary tumour samples from patient CRUKP9145. a, The anatomical location of the five primary tumour samples (region 2, 3, 4, 5 and 8) that were single-cell sequenced (colours), and the location of three additional samples for which bulk sequencing was previously performed (grey). **b,** The number of cells (x-axis) sequenced with DLP+ from each sample in the primary tumour. **c,** A kernel density estimate of the distribution of sequencing coverage (x-axis) for all single-cell sequenced cells from each primary tumour sample (y-axis). **d,** A kernel density estimate of the distribution of total read counts (x-axis) for all single-cell sequenced cells from each primary tumour sample (y-axis). **e,** A kernel density estimate of the distribution of the cell ploidy inferred by SPRINTER (x-axis) for all cells assigned to a clone in each primary tumour sample (y-axis).

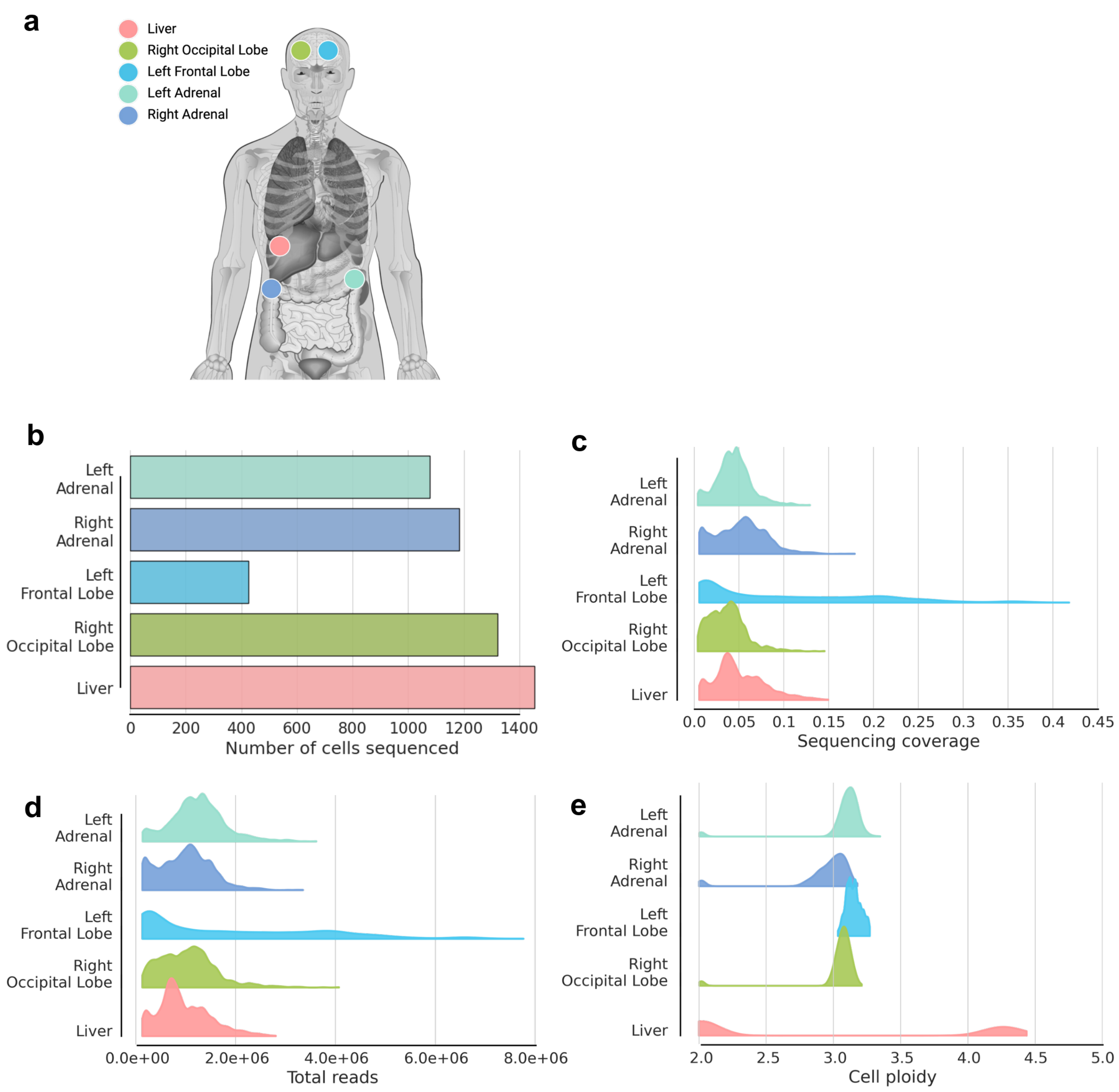

Supplementary Fig. 25: Sequencing details of all single cells sequenced from five metastatic sites from patient CRUKP9145. a, The anatomical location of the five different metastases that were single-cell sequenced (circles on body map). **b,** The number of cells (x-axis) sequenced with DLP+ from each metastasis (y-axis). **c,** A kernel density estimate of the distribution of sequencing coverage (x-axis) for all single-cell sequenced cells from each metastatic site (y-axis). **d,** A kernel density estimate of the distribution of total read counts obtained for all cells sequenced from every metastasis (y-axis). **e,** A kernel density estimate of the distribution of the cell ploidy inferred by SPRINTER (x-axis) for all cells assigned to a clone by SPRINTER in each metastatic site (y-axis).

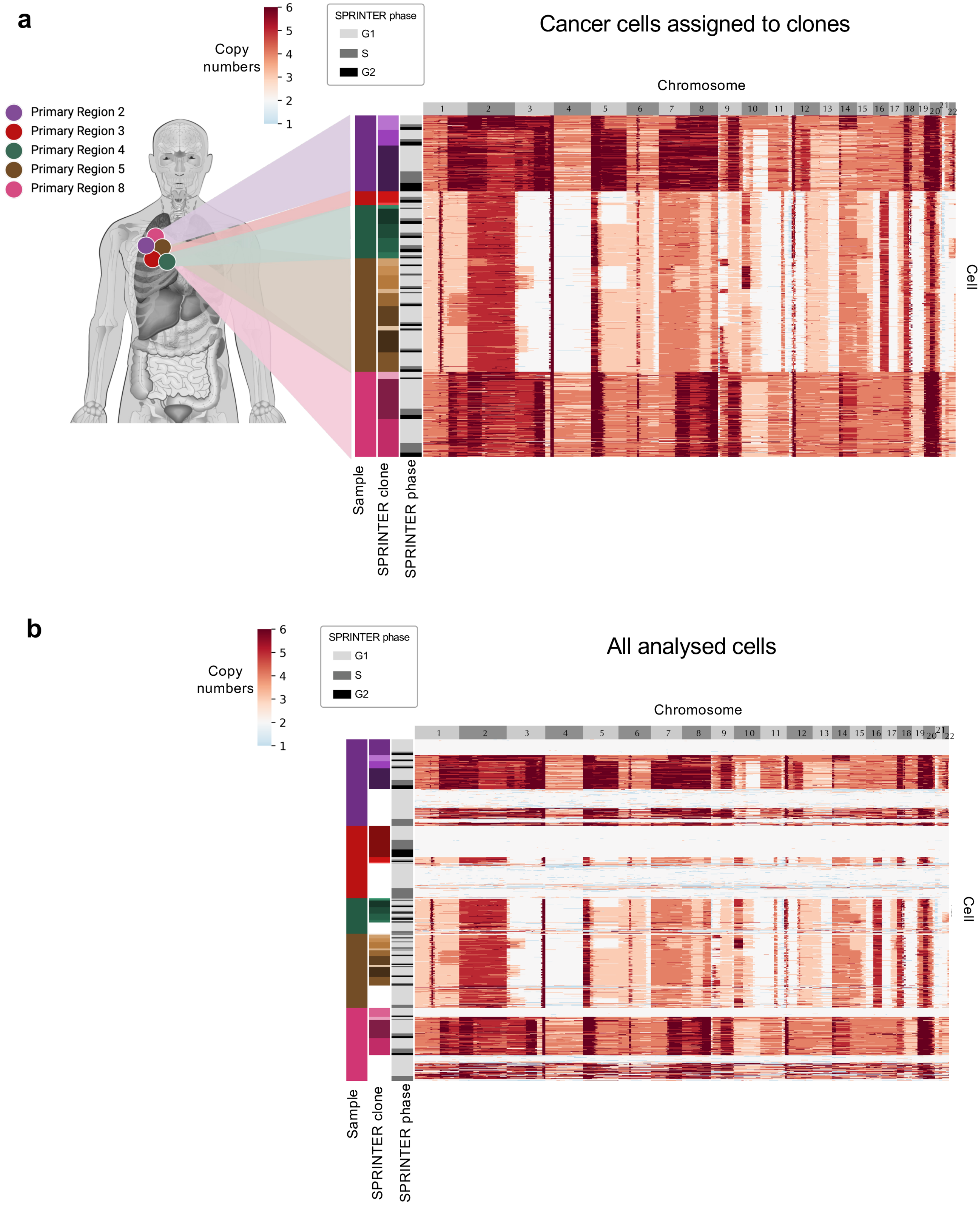

Supplementary Fig. 26: SPRINTER's results for cells sequenced from five primary tumour samples from patient CRUKP9145. Baseline copy numbers (heatmap colours) were inferred by SPRINTER on 9,532 cells (rows) sequenced from five primary tumour samples (left bar) across ~1Mb genomic bins (columns), displaying only the cancer cells assigned to clones (4,265 cells) (**a**) or all cells (**b**). SPRINTER-inferred clones (middle bar) and assigned S phase and G2 cells to each clone (light grey for G1 phase, dark grey for S phase, and black for G2 phase in right bar). The anatomical location of the samples (coloured circles) is displayed in (**a**).

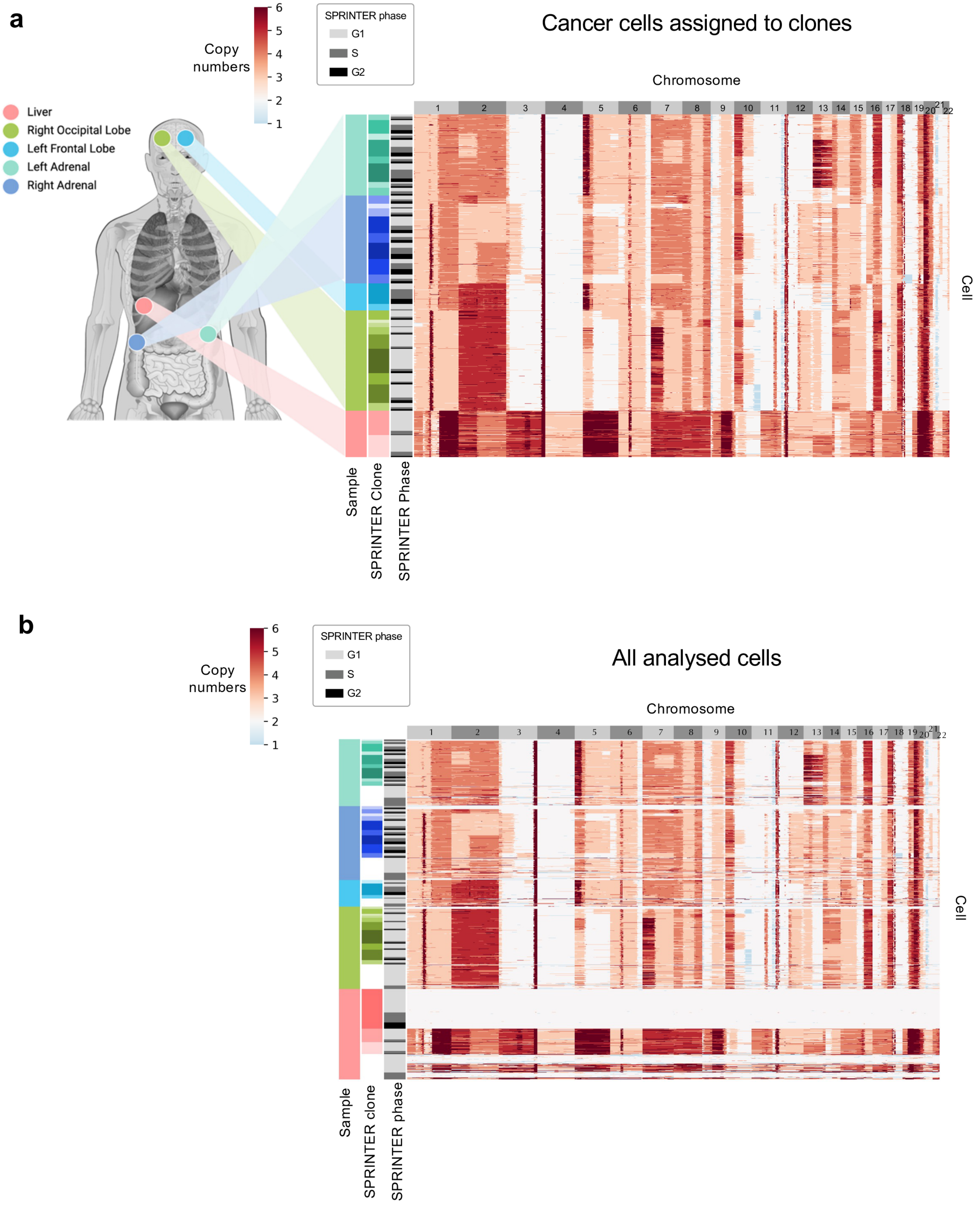

Supplementary Fig. 27: SPRINTER's results for cells sequenced from five metastases from patient CRUKP9145. Baseline copy numbers (heatmap colours) were inferred by SPRINTER on 5,462 cells (rows) sequenced from five metastases (left bar) across ~1Mb genomic bins (columns), displaying only the cancer cells assigned to clones (3,047 cells) (**a**) or all cells (**b**). SPRINTER-inferred clones (middle bar) and assigned S phase and G2 cells to each clone (white for G1 phase, grey for S phase, and black for G2 phase in right bar). The anatomical location of the samples (coloured circles) is displayed in (**a**).

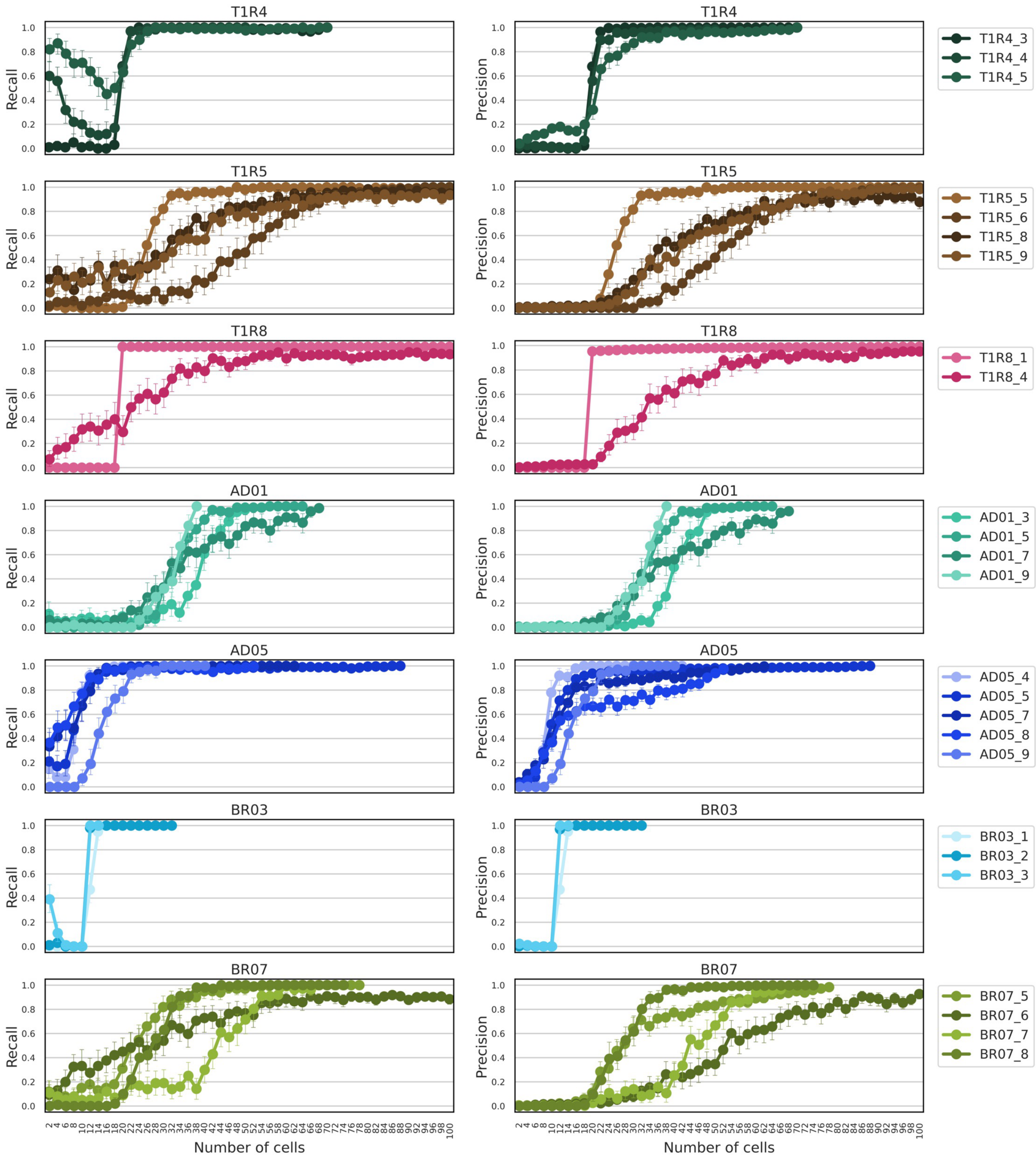

Supplementary Fig. 28: SPRINTER accurately recovers most clones containing more than 30 cells. SPRINTER was run on 125,000 datasets obtained by randomly subsampling a varying number of cells (x-axis, with 50 values between 1 and 100) from each clone (colour, 25 in total) in each NSCLC sample (row) for 100 repeats. Each dataset is obtained by adding the subsampled cells to all other remaining cells sequenced from the same sample. Recall (y-axis on left-side plots) and precision (y-axis on right-side plots) are measured for each subsampled clone in each dataset generated (each dot represents the mean across repeats and whiskers indicate the related standard deviation).

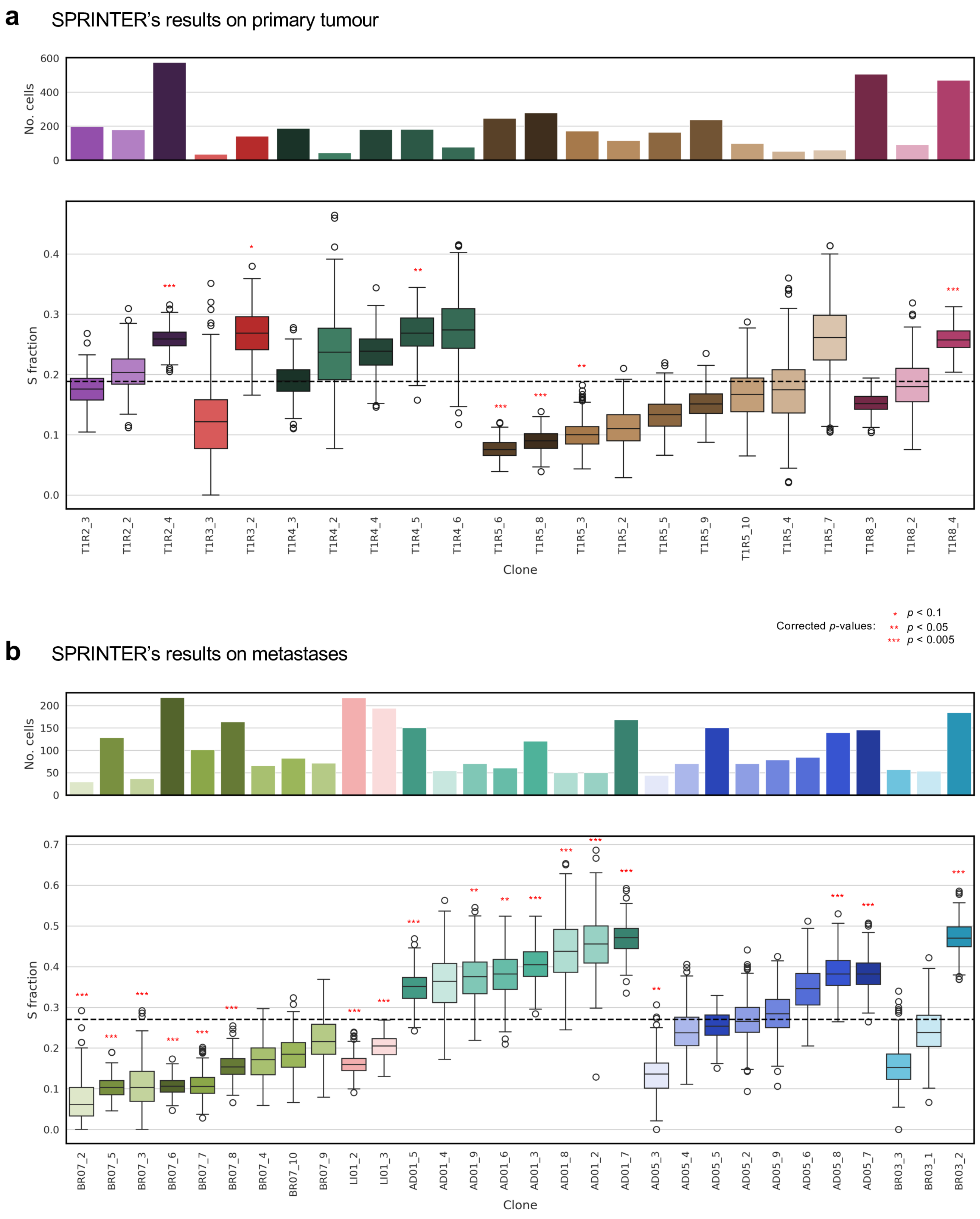

Supplementary Fig. 29: Clones have significantly different S fractions compared to the average S fraction in the tumour from patient CRUKP9145. The distribution of the S fraction (bottom y-axis) of each clone identified by SPRINTER (x-axis) with varying numbers of cells (top y-axis) in (**a**) five primary tumour samples and (**b**) five metastases were calculated by bootstrapping (with 300 samples) using the S phase cells identified and assigned to clones by SPRINTER. The S fraction of each clone was compared to the average tumour S fraction (dashed black line) using a two-sided Binomial test, applying the Benjamini-Hochberg method for multiple hypothesis correction with family-wise error rate 0.1 (stars indicate significant corrected p-values). Box plots show the median and the interquartile range (IQR), and the whiskers denote the lowest and highest values within 1.5 times the IQR from the first and third quartiles, respectively.

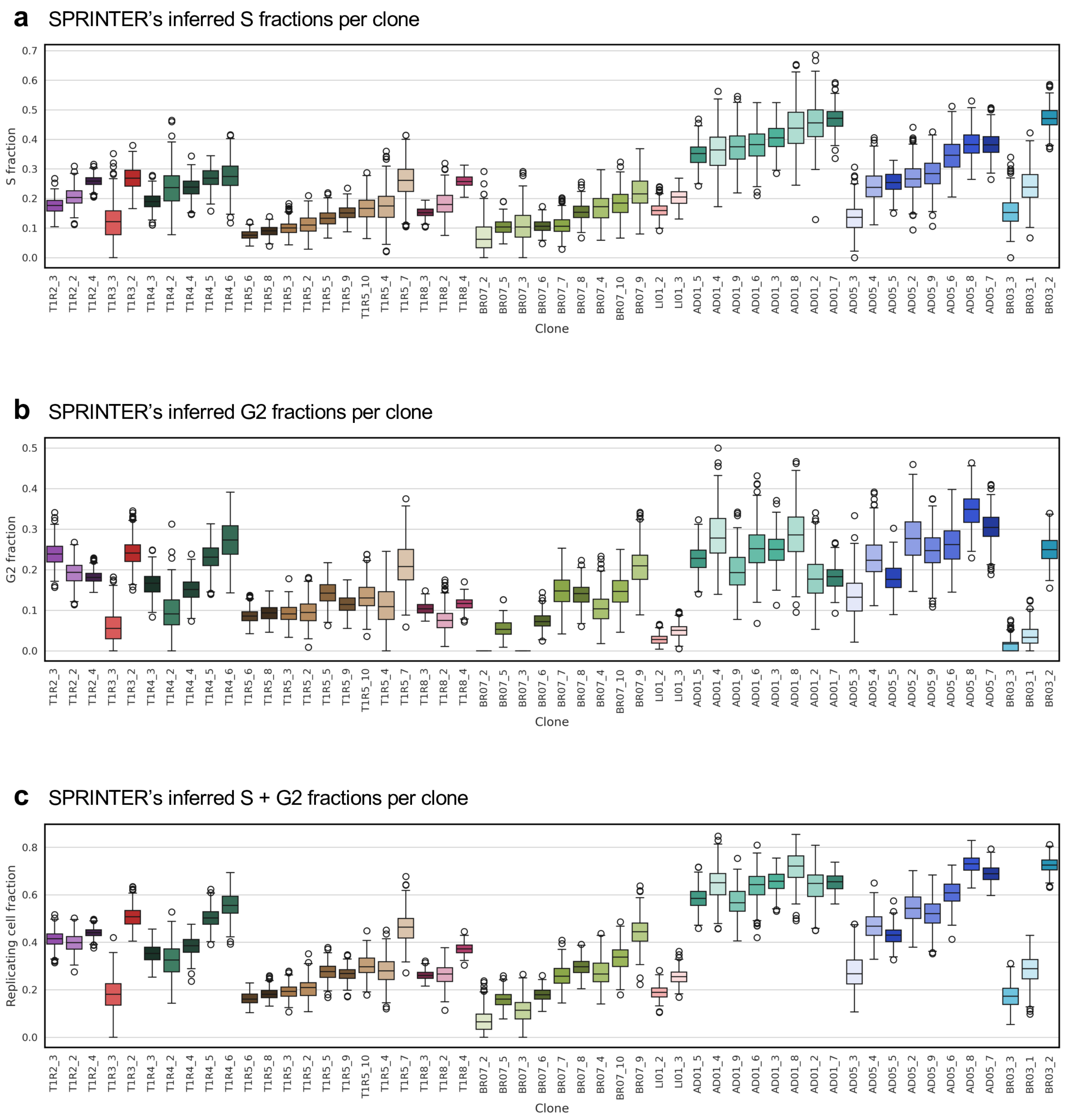

Supplementary Fig. 30: Patterns of proliferation measured by SPRINTER are consistent when considering S fraction, G2 fraction, or the fraction of total replicating cells. a-c, The distribution of the (**a**) S fraction, (**b**) G2 fraction, and (**c**) total replicating cell (S and G2) fraction (y-axis) of distinct SPRINTER-identified clones (x-axis) in all primary and metastatic tumour samples were calculated by bootstrapping (with 300 repeats) using S phase and G2 cells identified and assigned to clones by SPRINTER. Box plots show the median and the interquartile range (IQR), and the whiskers denote the lowest and highest values within 1.5 times the IQR from the first and third quartiles, respectively.

a Relationship between S and G2 fractions in CRUKP9145

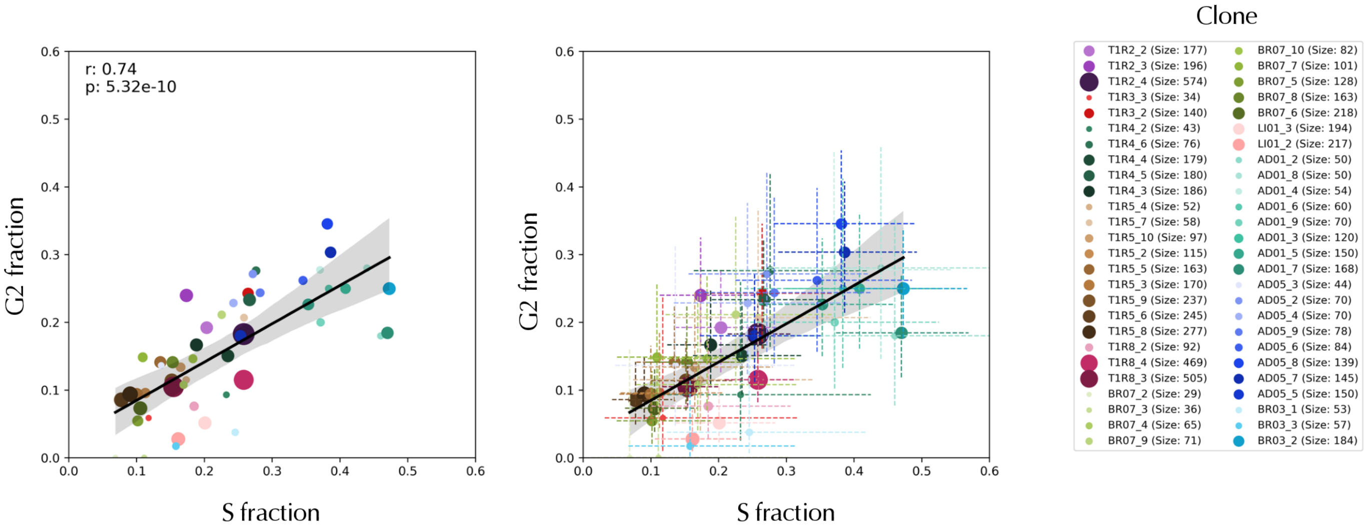

b Comparison of S and G2 fractions with overall expectation per sample

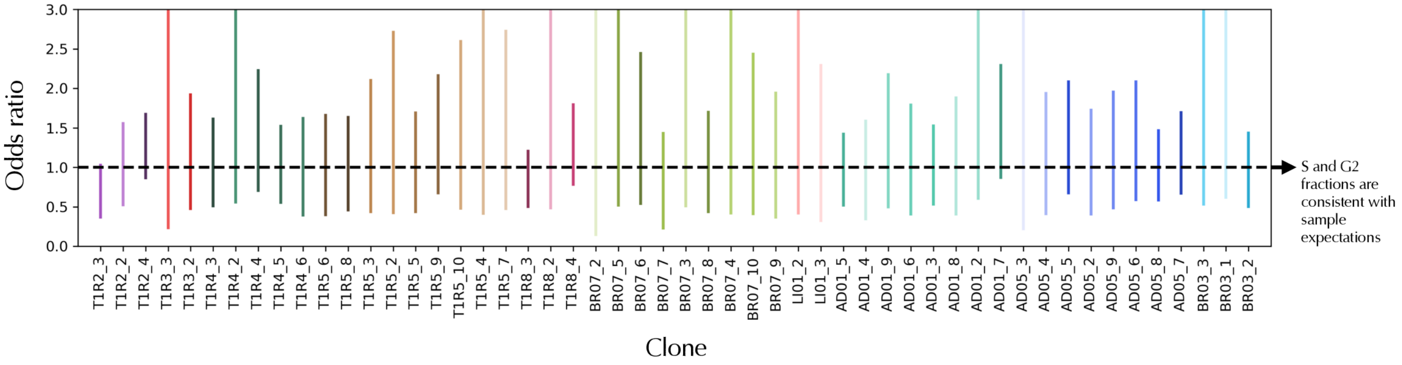

Supplementary Fig. 31: The S and G2 fractions in the NSCLC dataset have a high significant linear correlation. a, The estimated S (x-axis) and G2 (y-axis) fractions are estimated by SPRINTER for each clone in the NSCLC dataset (coloured circle with size proportional to number of cells) without (left) or with (right) estimated 99% confidence intervals around the median (dashed lines). The best linear regression is calculated (black line with the 99% confidence interval represented by the shaded area). **b,** The 95% confidence interval for the odds ratio (vertical lines on y-axis) of the relative rate of G2 over S fractions was calculated for each clone (x-axis) and compared with the corresponding sample expectation (dashed line, with the consistency for each sample indicated by the vertical lines of similar colours crossing the dashed horizontal line).

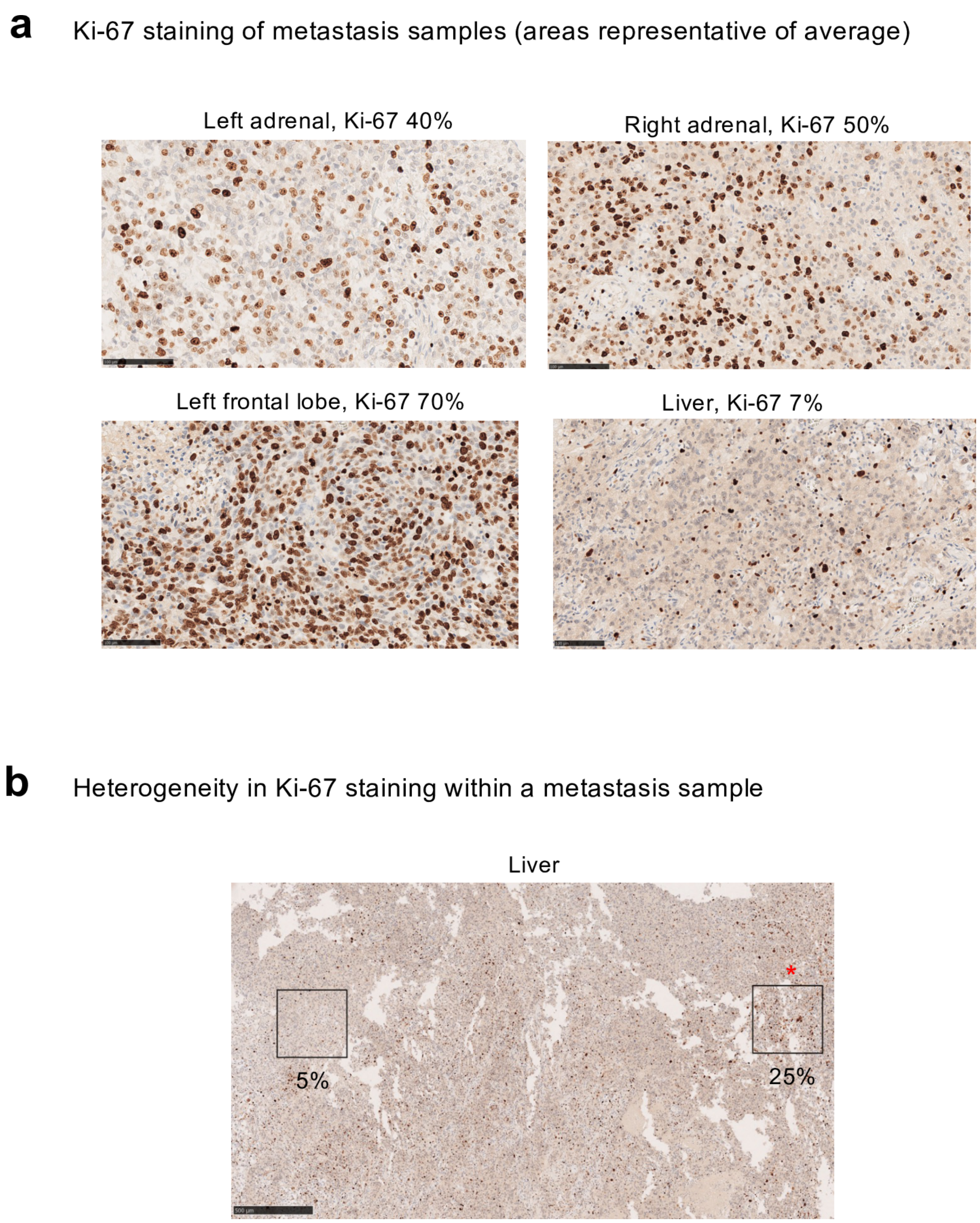

Supplementary Fig. 32: Ki-67 staining of tumour slides of metastases from patient CRUKP9145. a, FFPE slides showing areas representative of the average Ki-67 in each available metastasis sample. S phase cancer cells are stained with Ki-67 (brown). **b,** Heterogeneity in Ki-67 within an FFPE slide in the liver metastasis (boxes). SPRINTER results are broadly consistent with the selected area of Ki-67 (~25%, red star). In all cases, one representative slide taken as close as possible to the sequenced tissue was reviewed.

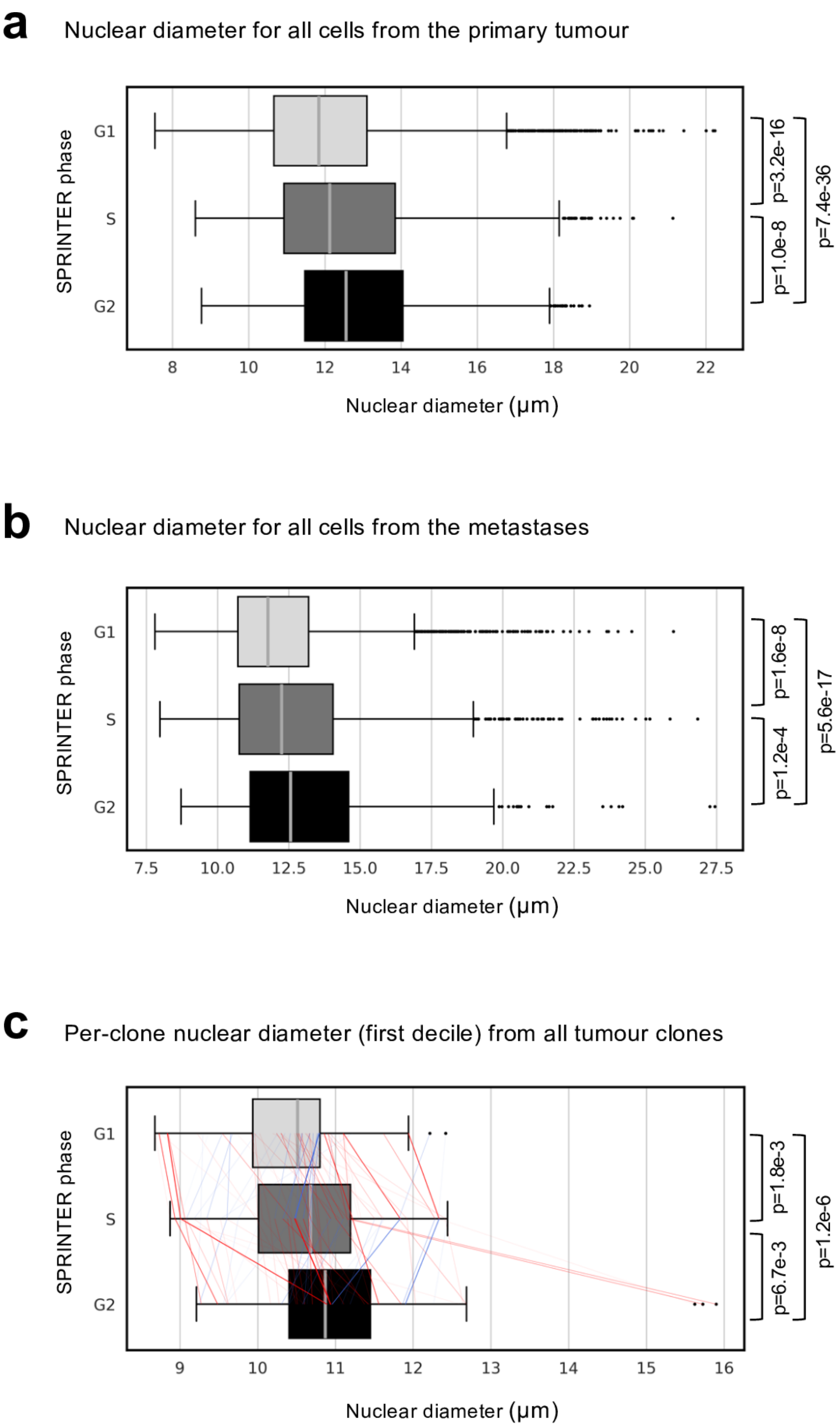

Supplementary Fig. 33: DLP+ nozzle-based nuclear imaging validates SPRINTER's phase predictions in primary tumour and metastatic samples from patient CRUKP9145. a-b, Nuclear diameter (x-axis) was measured (in micrometres and normalised by the mean of each sample) by DLP+ nozzle-based imaging for every cell with successfully recorded images from (**a**) primary tumour (total 9,532 cells) and (**b**) metastatic (total 5,037 cells) samples inferred by SPRINTER to be in G1, S or G2 phase. Each pair of distributions was compared using a one-sided Mann-Whitney U test (*p*-values reported on the right side). **c,** The nuclear diameter per clone (x-axis) was calculated using the first decile across all the cells in each clone (each dot) that were assigned to different cell cycle phases by SPRINTER (y-axis). Across cell cycle phases, clones are linked by lines, such that the line width is proportional to clone size and the line colour indicates whether the nuclear diameter per clone has increased as expected (red) or not (blue). Nuclear diameters in different cell cycle phases were compared per clone using a one-sided Wilcoxon signed-rank test (*p*-values on right). In all panels, box plots show the median and the interquartile range (IQR), and the whiskers denote the lowest and highest values within 1.5 times the IQR from the first and third quartiles, respectively.

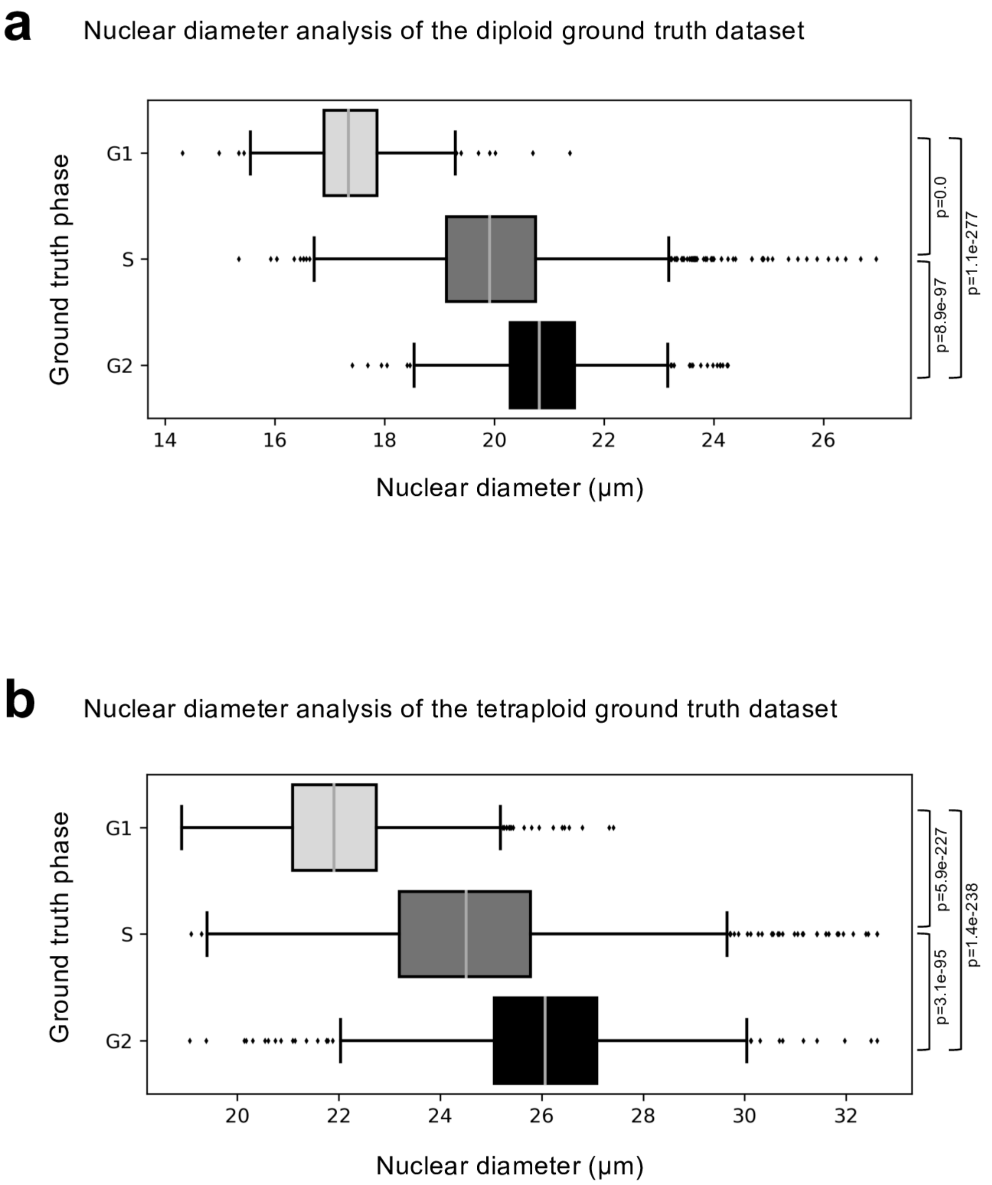

Supplementary Fig. 34: Nuclear diameter is related to cell cycle phase in the ground truth datasets. Nuclear diameter (x-axis) is measured (in micrometres) by DLP+ nozzle-based imaging for every sorted G1, S, and G2 phase cell in the (**a**) diploid and (**b**) tetraploid ground truth datasets (for all 5,051 diploid and 5,154 tetraploid cells with imaged nuclei, including cells with <100k sequencing reads). Each pair of distributions was compared using a one-sided Mann-Whitney U test (p-values reported on the right side). In all panels, box plots show the median and the interquartile range (IQR), and the whiskers denote the lowest and highest values within 1.5 times the IQR from the first and third quartiles, respectively.

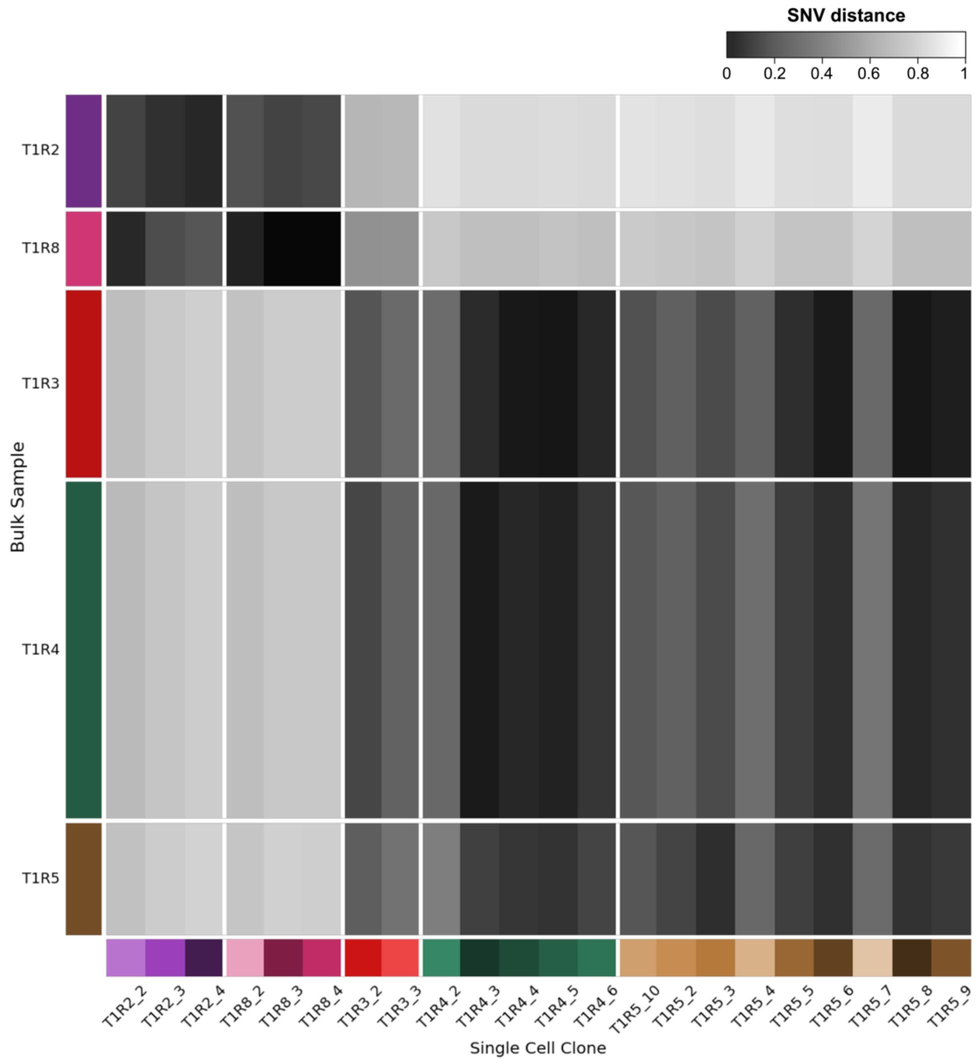

Supplementary Fig. 35: Primary tumour clones inferred by SPRINTER consistently harbour SNVs most similar to the SNVs obtained from bulk sequencing of the corresponding sample. The SNV distance between each single-cell tumour clone inferred by SPRINTER (x-axis) and each bulk tumour sample analysed in previous studies (y-axis) was calculated as the fraction of different subclonal SNVs, with lower values indicating higher similarity (black) and higher values indicating lower similarity (white).

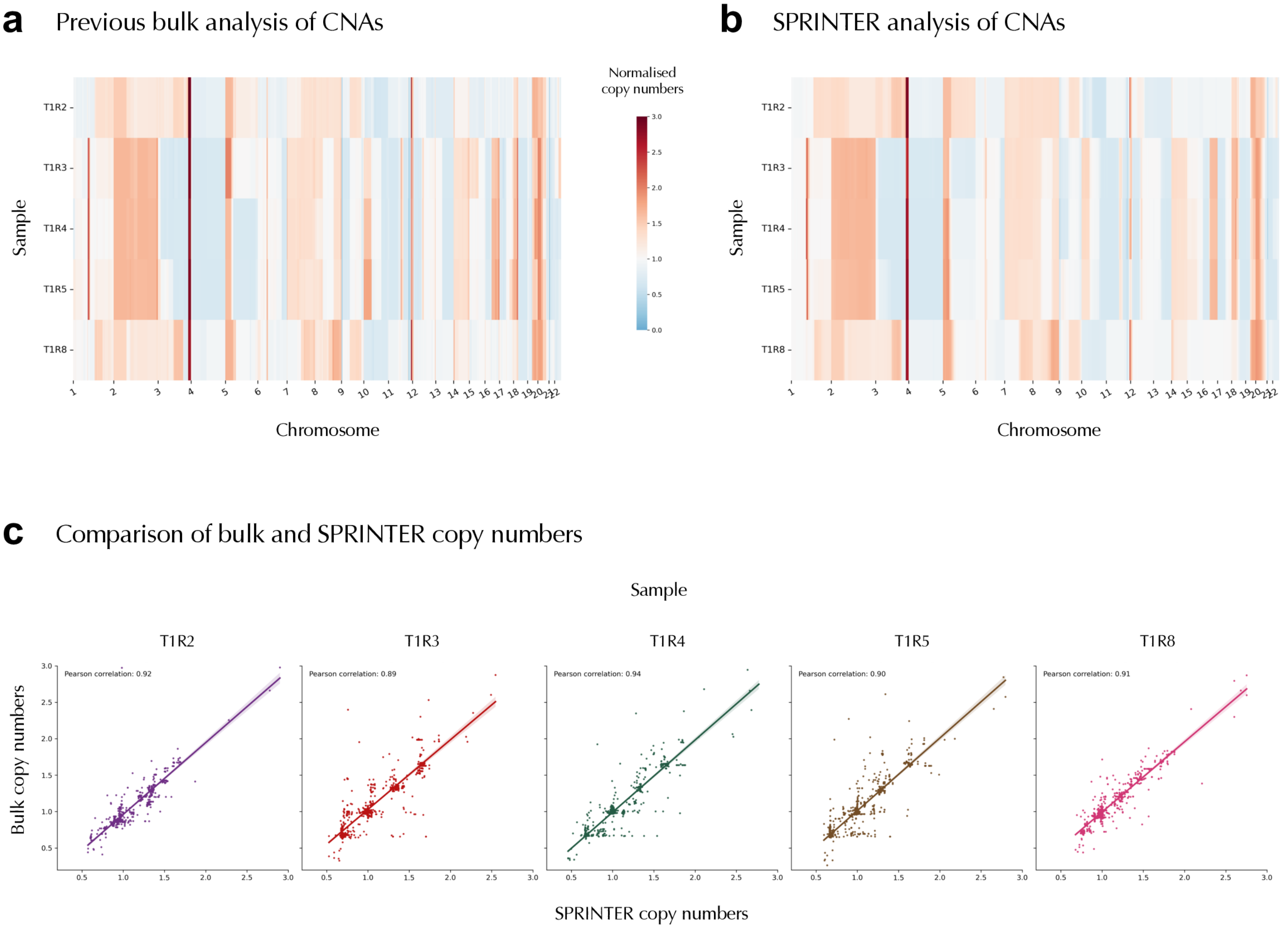

Supplementary Fig. 36: SPRINTER infers CNAs in the NSCLC dataset that are highly consistent with previous bulk studies of matched primary tumour regions. a, Normalised fractional copy numbers (colours, representing averages across all cells within the bulk sample) from previous bulk studies for five primary tumour regions (rows) in 1Mb genomic bins across all autosomes (columns) were calculated after correcting for tumour sample purity and ploidy. **b,** For each matched primary tumour region (rows), normalised copy numbers (colours) for the same 1Mb genomic bins across all autosomes (columns) were computed as the average of the copy numbers inferred by SPRINTER for all cancer cells sequenced from the same sample. **c,** For each primary tumour region (each plot), the normalised copy numbers inferred in previous bulk studies (y-axis) for 1Mb genomic bins (dots) is compared to normalised copy numbers calculated using SPRINTER. A Pearson correlation is computed for each sample (coefficient labelled at the top and linear relation represented as a line).

a All SNVs from primary tumour and metastases used for phylogenetic analysis

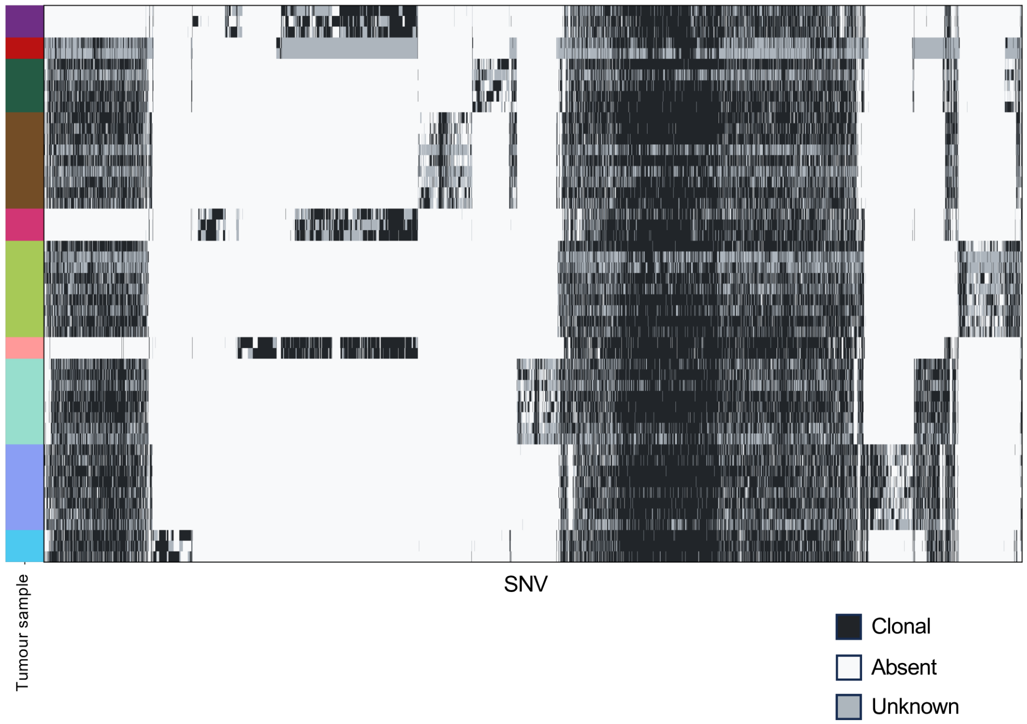

b Selected SNVs from primary tumour and metastases used to reconstruct tumour phylogeny

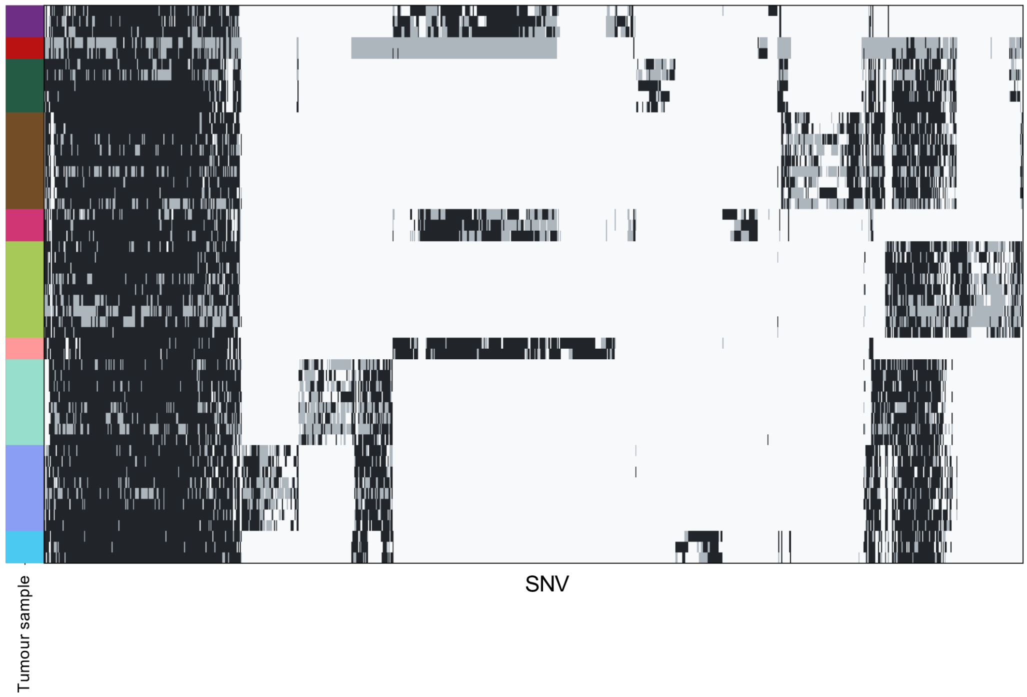

Supplementary Fig. 37: SNV genotypes used to reconstruct tumour phylogeny from the primary tumour and metastatic samples from patient CRUKP9145. SNVs in chromosomes 1, 3, 4, and 17 not affected by mutation losses (x-axis) are classified as clonal (black), absent (white), or unknown (grey) in each clone inferred by SPRINTER (y-axis) across primary tumour (first five colours) and metastatic (last five colours) samples when considering (**a**) all SNVs in those chromosomes and (**b**) the 1000 input SNVs used to reconstruct the tumour phylogeny.

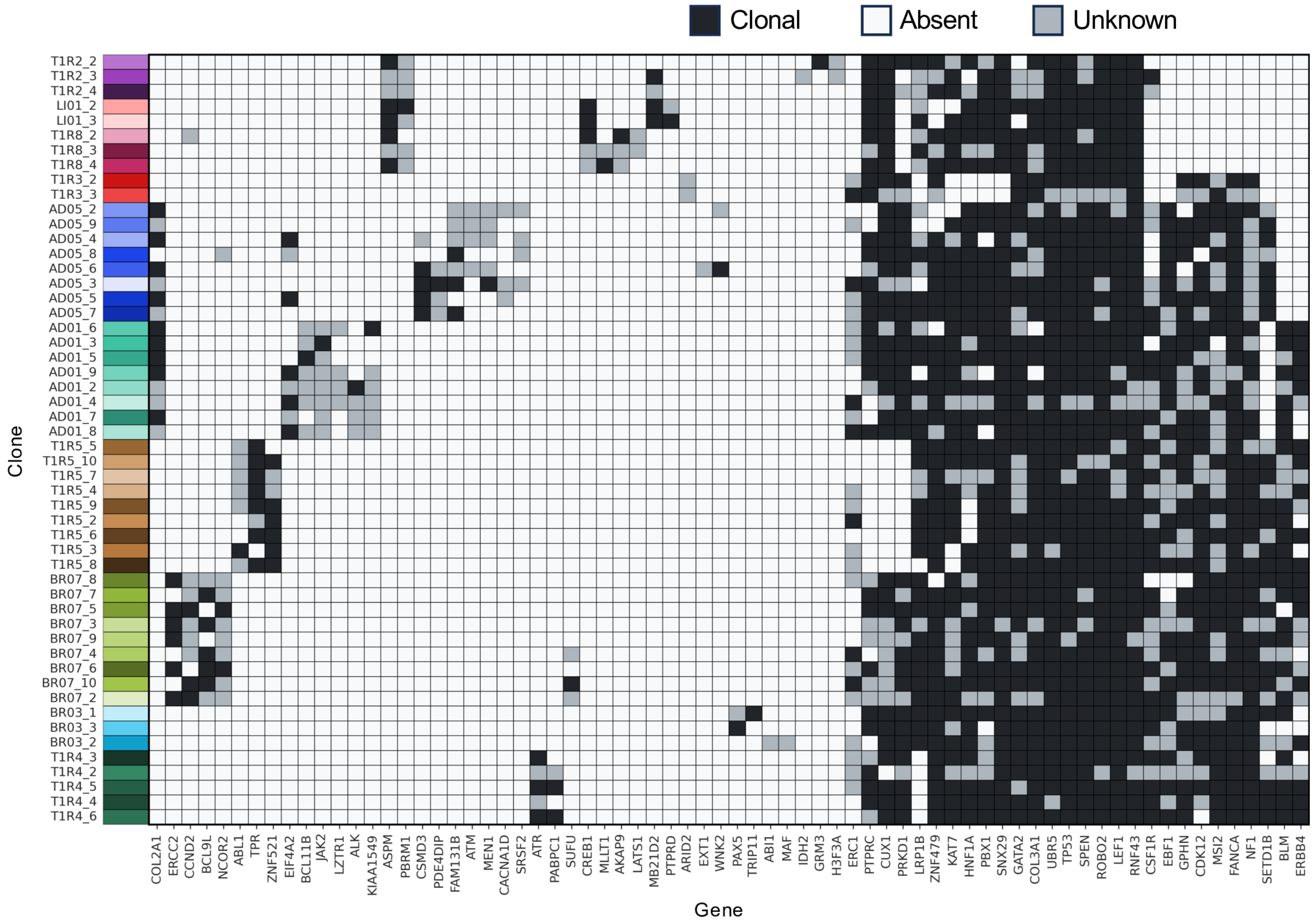

Supplementary Fig. 38: Identification of driver mutations in the clones inferred by SPRINTER from the primary tumour and metastatic samples from patient CRUKP9145. The driver mutations identified (with corresponding genes reported on the x-axis) are classified as clonal (black), absent (white), or unknown (grey) in each clone inferred by SPRINTER (y-axis) across primary tumour and metastatic samples (shades of different colours).

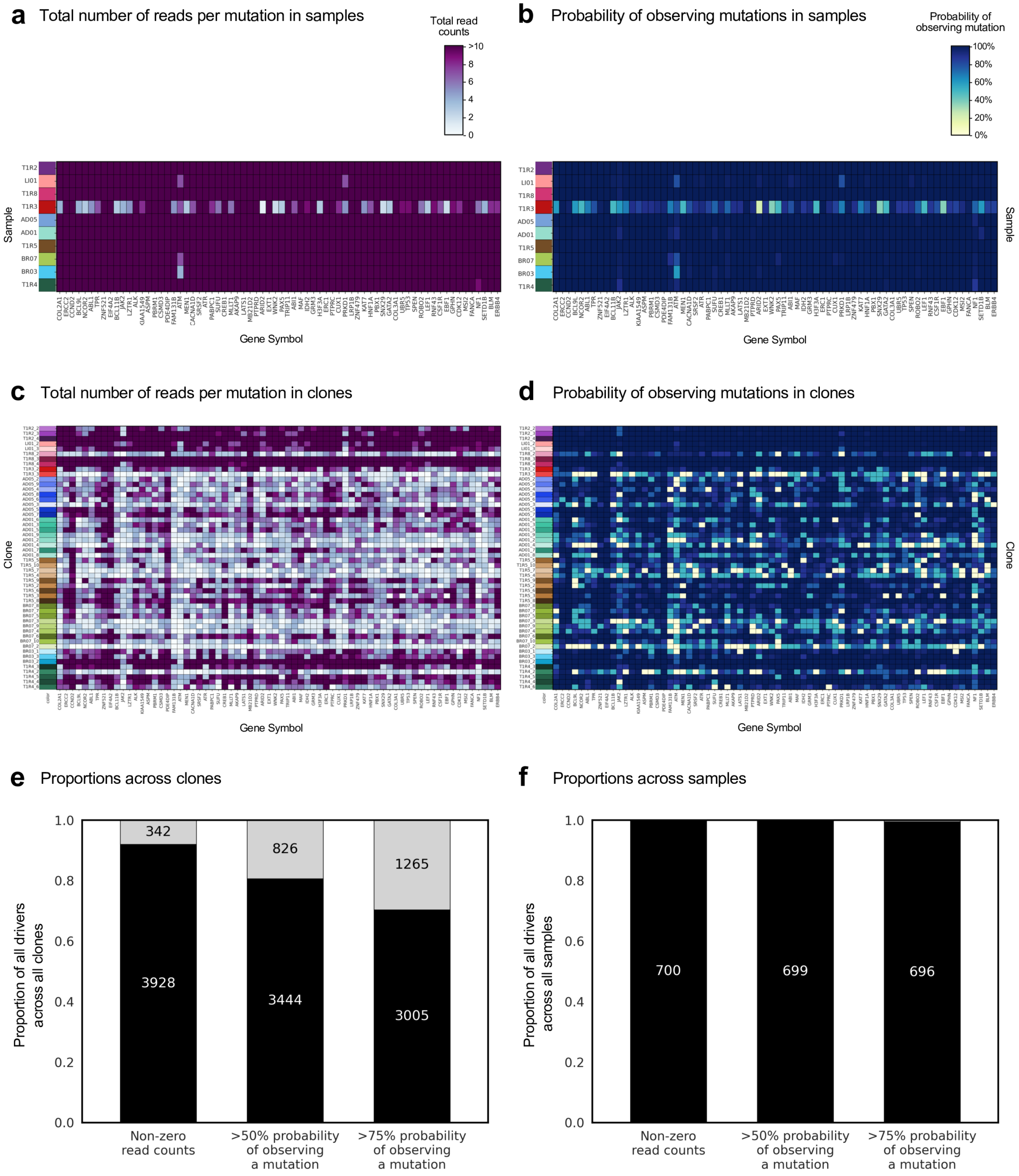

Supplementary Fig. 39: The novel NSCLC dataset provides sufficient power to identify most mutations across samples and clones. a-b, For each driver mutation in each gene (x-axis) and in every sample (y-axis), the total number of reads covering the corresponding genomic locus (**a**) and the probability of observing more than one variant read computed using a binomial model with an underlying mutant allele frequency of 10% (**b**) are reported. **c-d,** For each driver mutation in each gene (x-axis) and in every SPRINTER clone (y-axis), the total number of reads covering the corresponding genomic locus (**c**) and the probability of observing more than one variant read computed using a binomial model with an underlying mutant allele frequency of 50% (**d**) are reported. **e-f,** The proportion (y-axis) and number (labels) of all loci with non-zero read counts, >50% probability of observing a mutation if present, and >75% probability of observing a mutation if present (x-axis) were computed either across all SPRINTER clones (**e**) or across all samples (**f**).

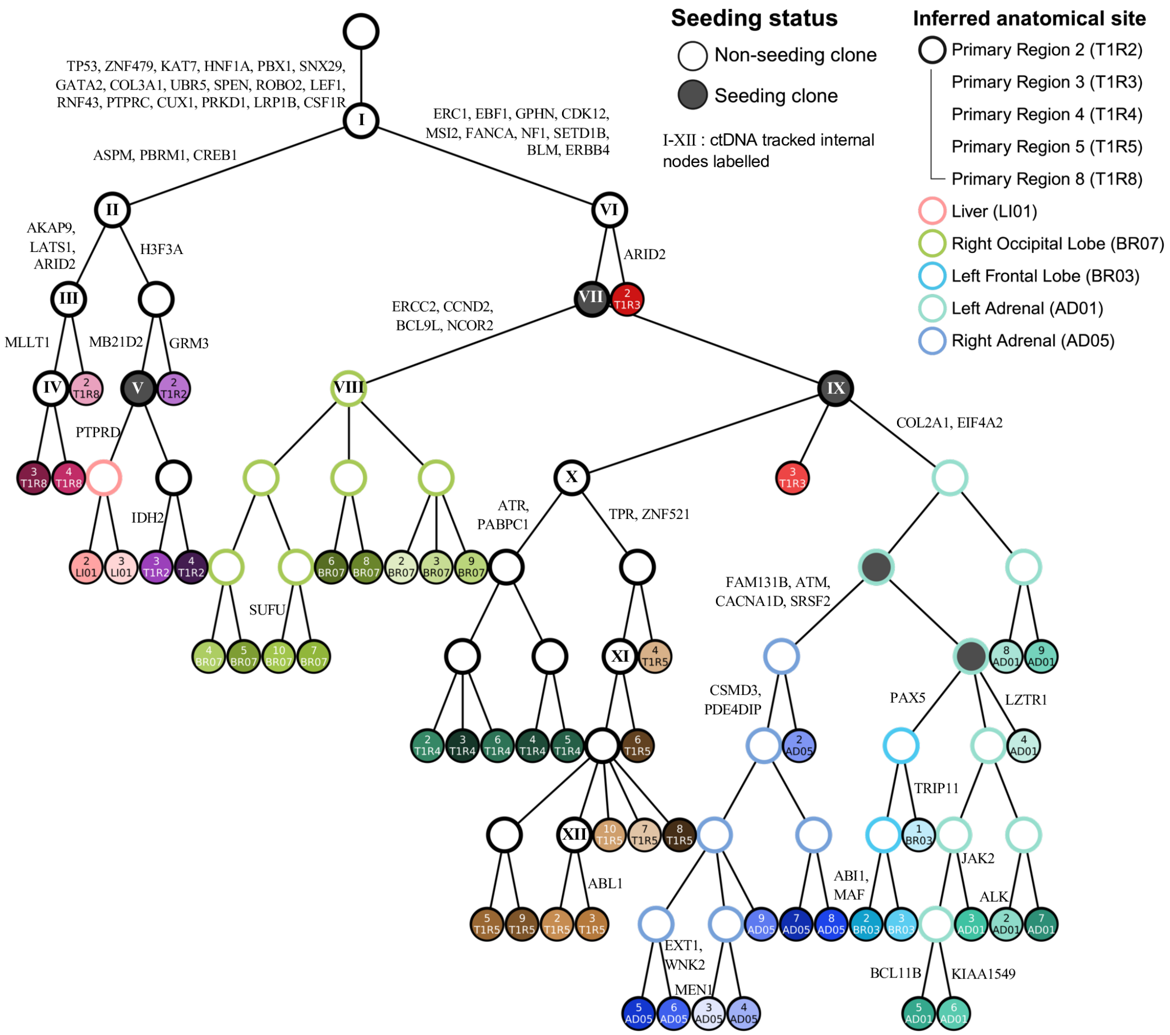

Supplementary Fig. 40: Reconstructed evolution of driver mutations in the NSCLC dataset. The tumour phylogeny was reconstructed using the SNVs from SPRINTER's single-cell clones (leaves of the tree) in the primary tumour and metastatic samples from patient CRUKP9145 (clones are uniquely coloured with source samples represented by shades of the same colour). Seeding clones (dark grey) and the anatomical location of the remaining ancestral clones (white internal colour with border coloured according to the inferred anatomical site) were inferred using the MACHINA algorithm. SNVs tracked in a previous ctDNA study were harboured by some ancestral clones (roman numerals). The edges of the phylogeny are labelled with the driver mutations that were inferred to occur in those edges.

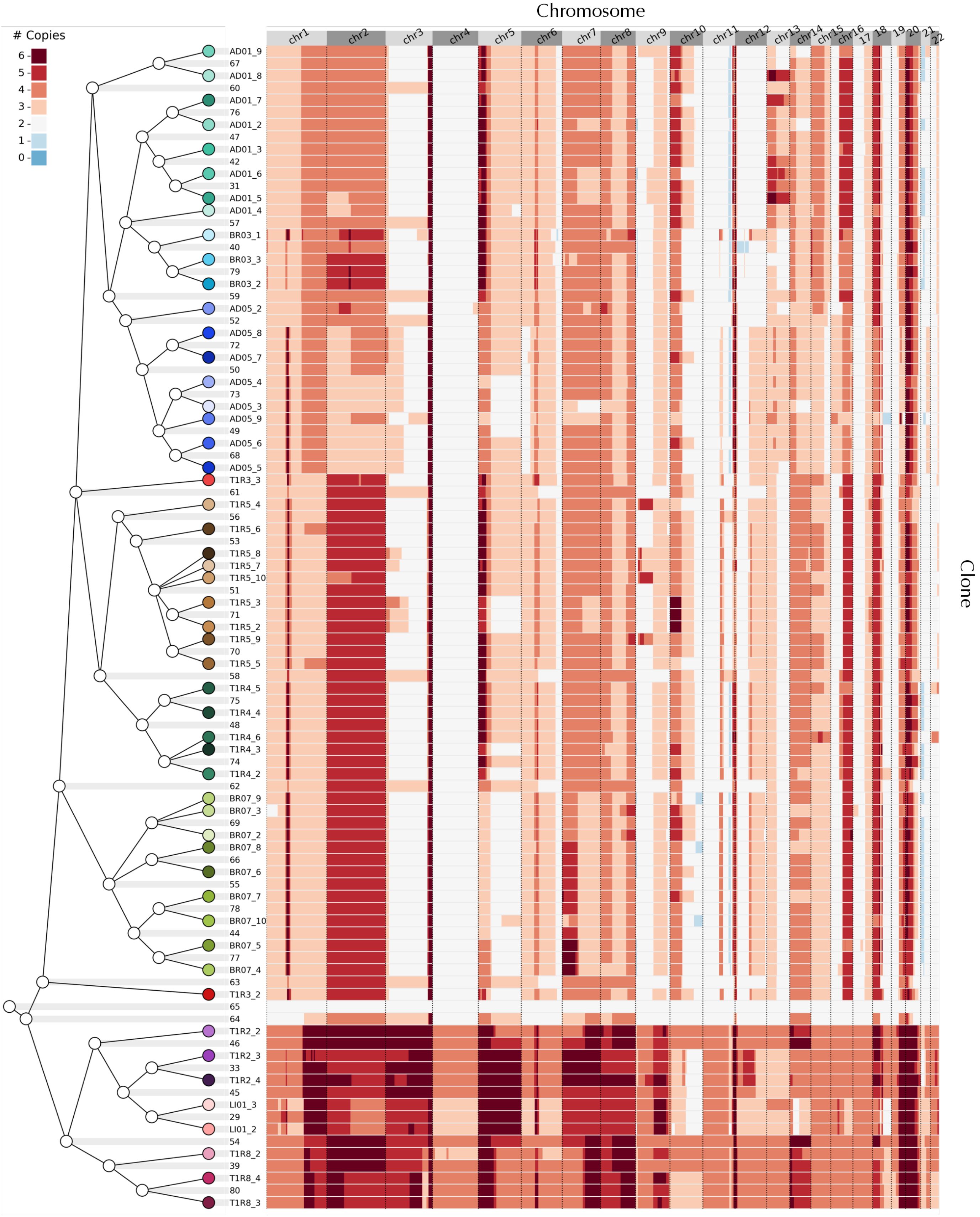

Supplementary Fig. 41: Reconstructed evolution of CNAs for the NSCLC dataset. CNA evolution was reconstructed by the MEDICC2 algorithm, which took as input the SNV tree topology and the copy numbers (colours) inferred by SPRINTER across the genome (columns) for extant clones (rows with coloured circles) in order to infer the copy numbers of the ancestral clones (rows without coloured circles).

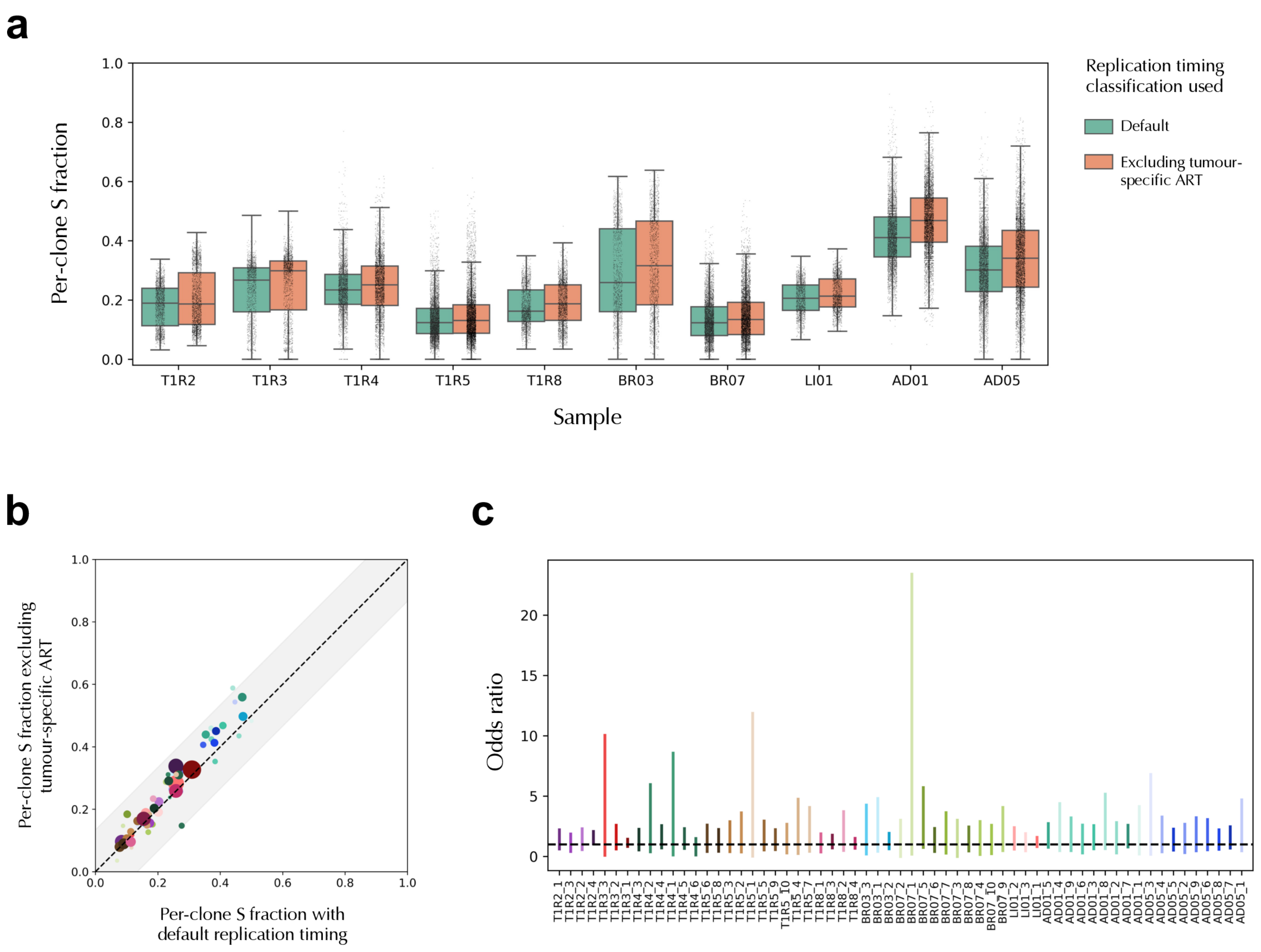

Supplementary Fig. 42: SPRINTER's estimates of S fractions are not affected by clone-specific altered replication timing (ART). a, The bootstrapped estimate of the S fraction of each clone (y-axis) was calculated by sampling cells with replacement in each SPRINTER-inferred clone from each sample of the NSCLC dataset (x-axis), using either the default replication timing classification (green) or excluding genomic regions in which high-confidence ART events (found in most clones in >2 samples) have been identified (orange). **b,** The fraction of S phase cells assigned to each clone (each dot was sized proportionally to the corresponding number of cells and with colours matching those defined in (**c**)) has been directly estimated by SPRINTER either using the default replication timing classification (x-axis) or excluding high-confidence ART events (y-axis). The expected range of uncertainty (grey shadow with diagonal represented as a dashed line) is calculated using the average size of the 99% confidence interval estimated using bootstrapping per clone from SPRINTER's results. **c,** The 99% confidence interval for the odds ratio (vertical lines on y-axis) was calculated for each clone (x-axis) comparing the S fractions estimated using the default replication timing classification to those excluding high-confidence ART events (dashed line, with the consistency between estimates indicated by the vertical lines crossing the dashed horizontal line).

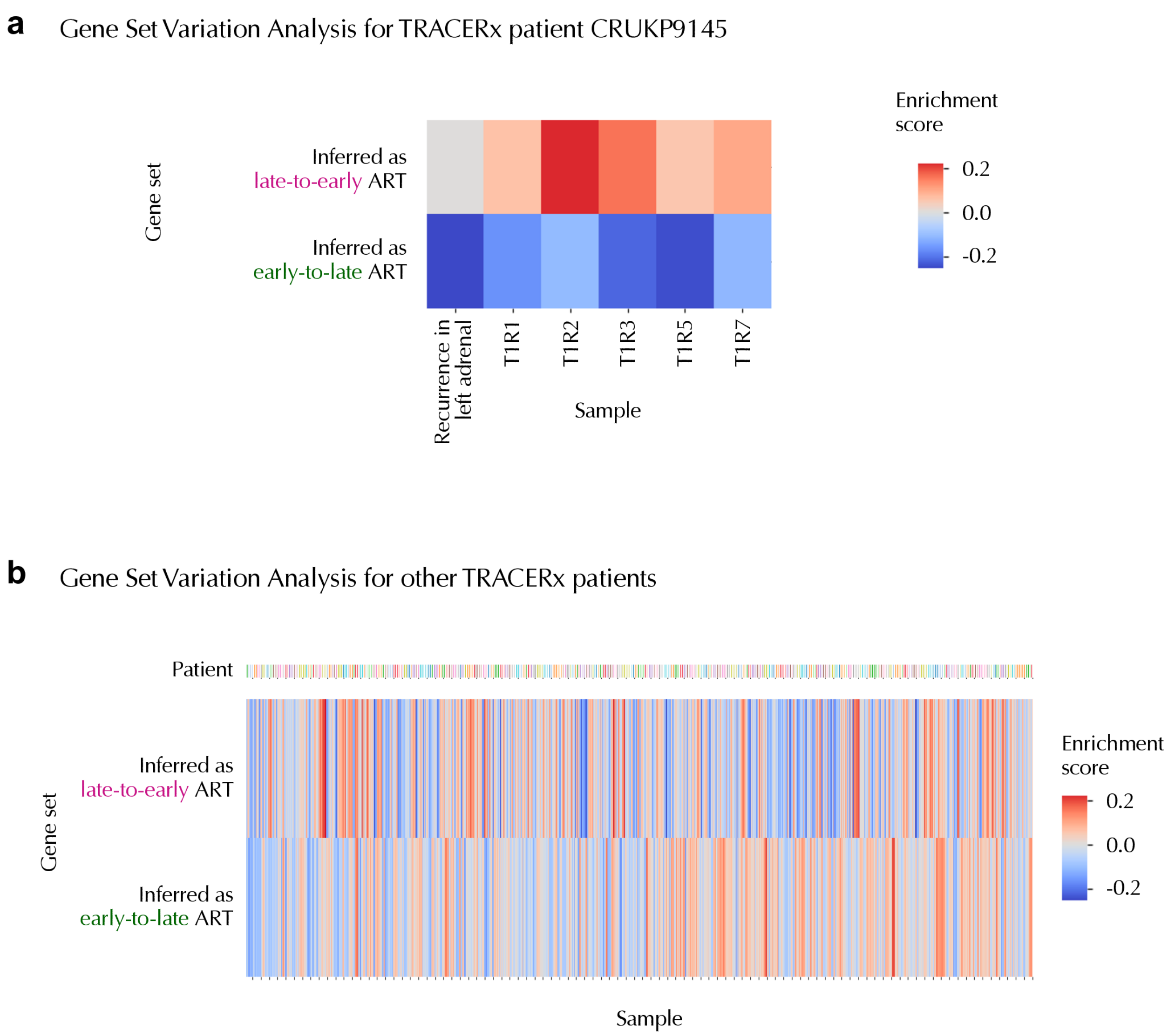

Supplementary Fig. 43: Gene set variation analysis using bulk RNA-sequencing in the TRACERx cohort supports the inferred ART for patient CRUKP9145. **a,** Gene set variation analysis enrichment scores (colours, with higher scores indicating increased expression, and lower scores indicating decreased expression) for the set of genes inferred to have altered replication timing (ART) in at least one sample from patient CRUKP9145 in either the late-to-early or the early-to-late direction (y-axis) in each CRUKP9145 tumour sample with available bulk RNA-sequencing data (including primary tumour regions and a pre-mortem recurrence sample in the left adrenal, x-axis). **b,** Gene set variation analysis enrichment scores (colours) calculated for the same gene sets with ART as defined in CRUKP9145 in (**a**), but for all tumour samples (total 915 samples) with available RNA-sequencing data (x-axis) from 347 other TRACERx patients (top bar).

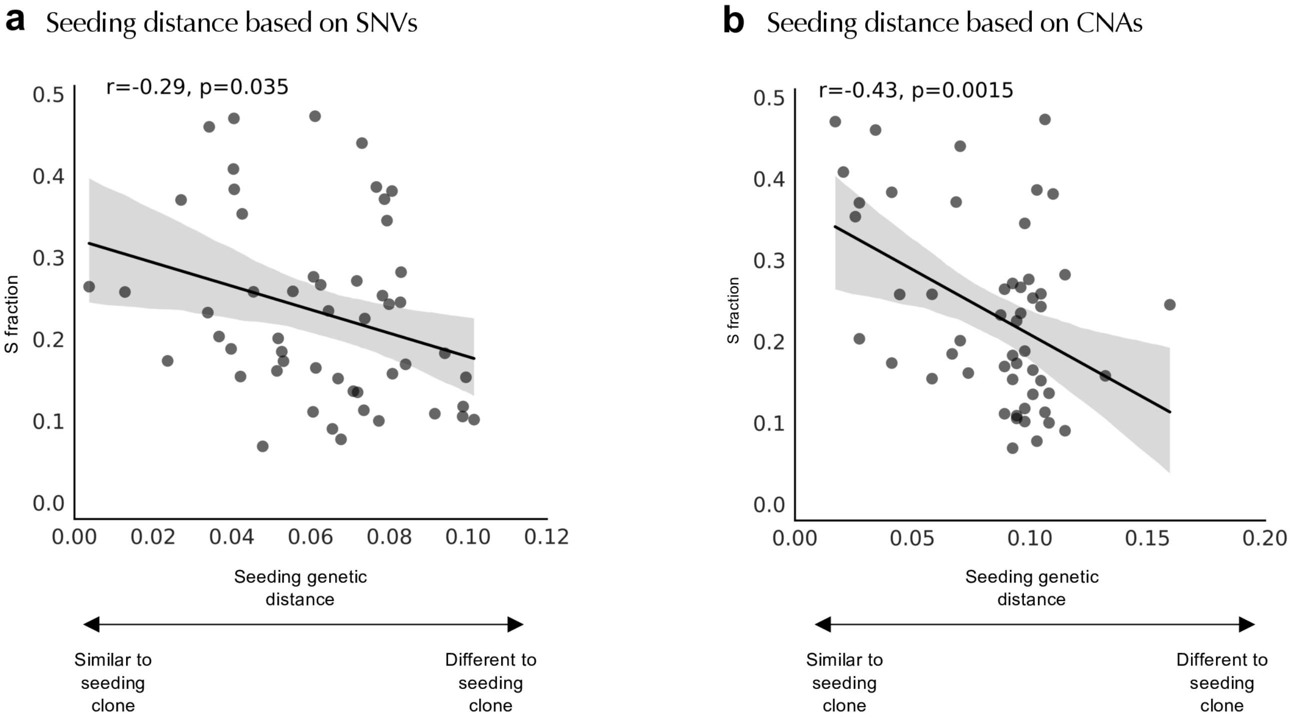

Supplementary Fig. 44: The negative correlation between seeding genetic distance and clone proliferation rate indicates that clones with more similarity to seeding clones have increased proliferation. For each SPRINTER clone (dot) inferred in the primary tumour and metastatic samples from patient CRUKP9145, SPRINTER's inferred S fraction (y-axis) was compared to the seeding genetic distance (x-axis) computed with respect to the closest seeding clone based on either (**a**) SNVs or (**b**) CNAs. In each case, a two-sided Pearson correlation test was performed (correlation coefficients and p-values are reported) and the 95% confidence interval was calculated for linear regressions (shaded areas).

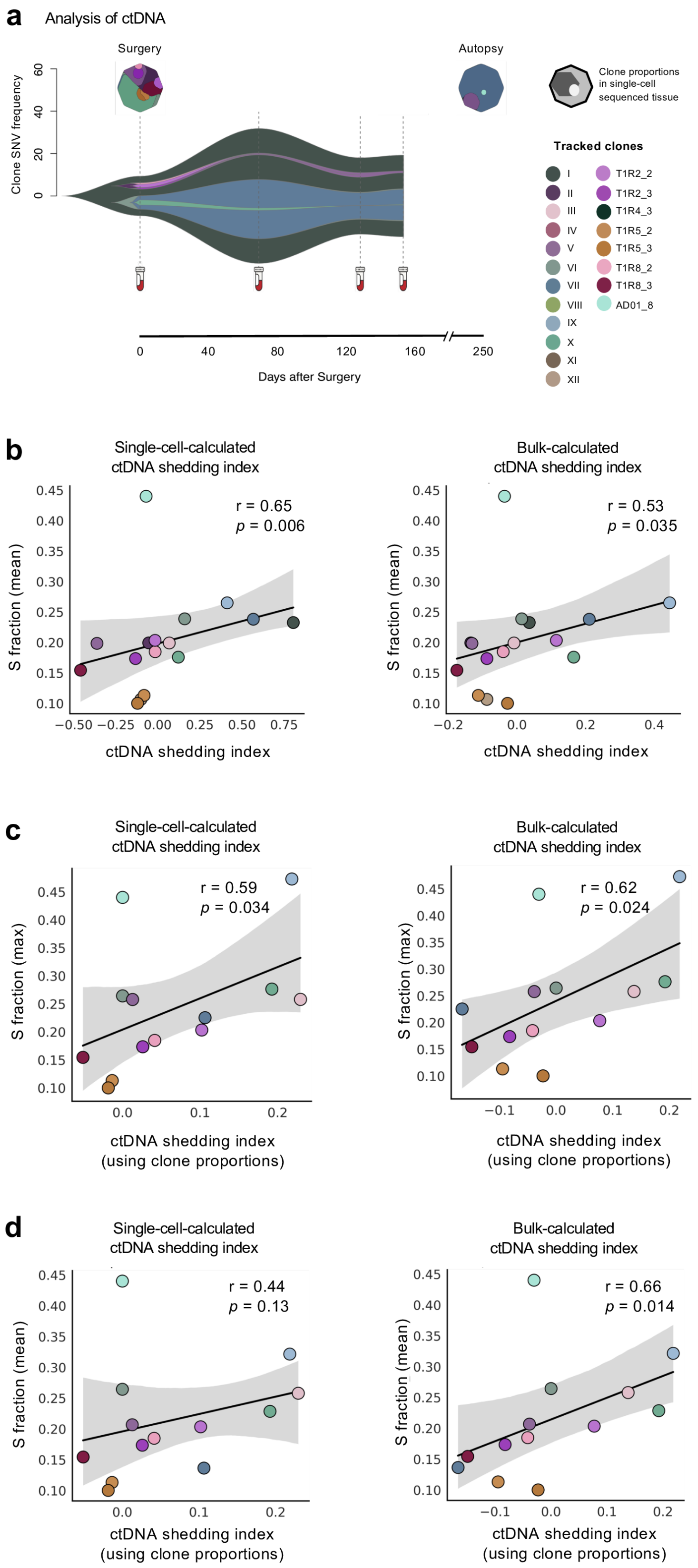

Supplementary Fig. 45: High clone proliferation rate is linked to increased clone-specific ctDNA shedding. a, The frequency of SNVs harboured by ancestral clones tracked with ctDNA (y-axis) was measured at four time points (dashed grey lines) across the disease course (x-axis in days, with surgery and autopsy times annotated). For each ctDNA-tracked clone (colours) the corresponding proportion of cancer cells was measured using scDNA-seq data at the surgical and autopsy time points (hexagons). **b,** For each ctDNA-tracked clone (dot), a ctDNA shedding index (x-axis) was calculated using the frequency of SNVs for either (left) SPRINTER clones or (right) previous bulk clones, and compared to the mean S fraction (y-axis) inferred from descendant SPRINTER clones. In each case, a two-sided Spearman correlation test has been performed (correlation coefficients and p-values are reported) and the 95% confidence interval has been calculated for linear regressions (shaded areas). **c-d,** For each ctDNA-tracked clone (dot), a ctDNA shedding index (x-axis) was calculated using the clone proportion (i.e., proportion of cells belonging to the clone) for either (left) SPRINTER clones or (right) previous bulk clones, and compared to the maximum (**c**) or mean (**d**) S fraction (y-axis) inferred from the uniquely assigned descendant SPRINTER clones. In each case, a two-sided Spearman correlation test has been performed (correlation coefficients and p-values are reported) and the 95% confidence interval has been calculated for linear regressions (shaded areas).

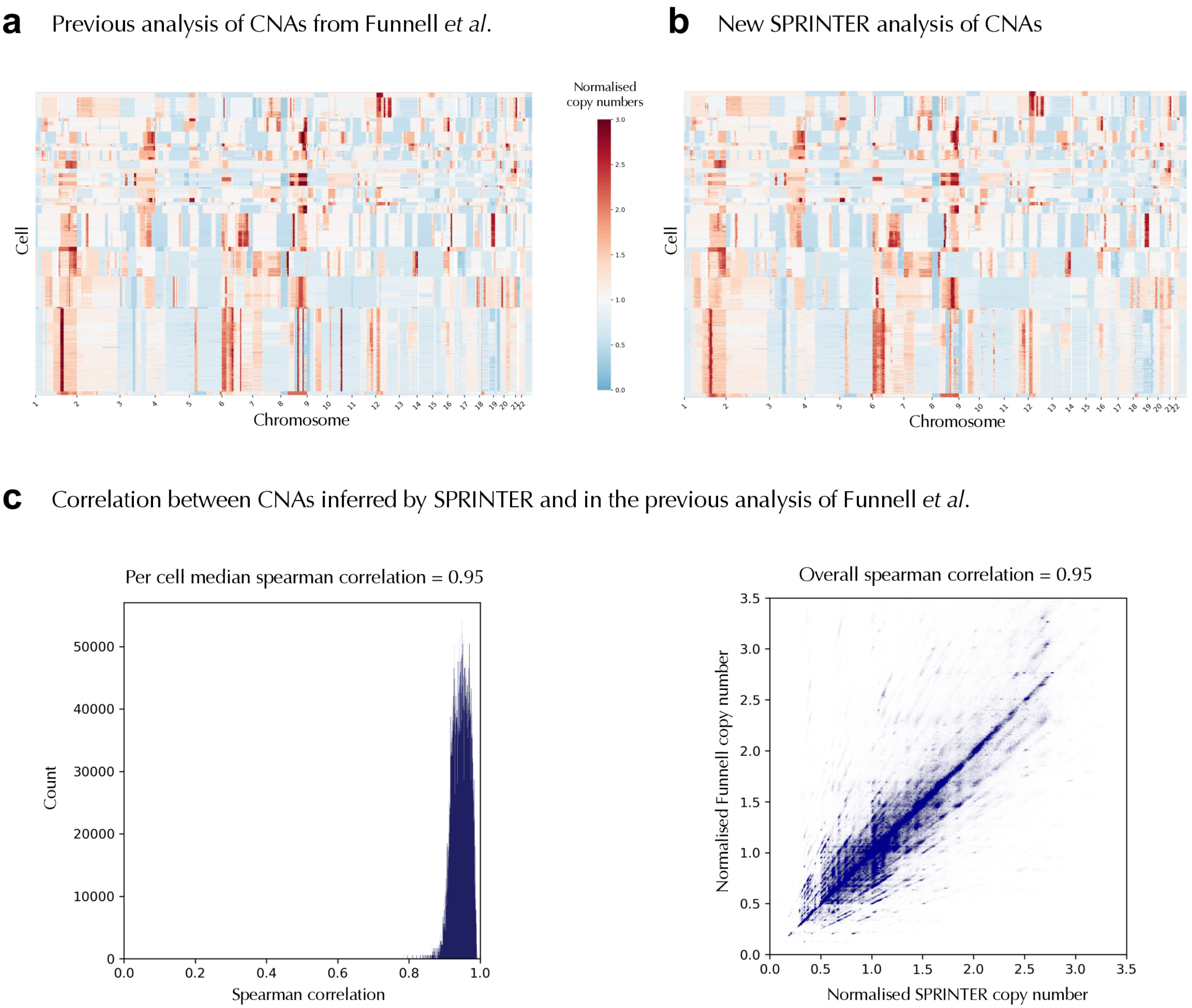

Supplementary Fig. 46: SPRINTER infers CNAs that are highly consistent with previous analysis by Funnell et al. a-b, Heatmaps of copy number normalised by ploidy (colours) in every non-S phase cell (y-axis) across the genome (x-axis) as inferred previously in the analysis by Funnell *et al.* (**a**) and by SPRINTER (**b**). **c,** (Left) Spearman correlation between copy numbers normalised by ploidy inferred previously in the Funnell *et al.* analysis and by SPRINTER in non-S phase cells (x-axis) measured per cell (y-axis showing count of cells). (Right) Copy numbers normalised by ploidy inferred by SPRINTER in non-S phase cells (x-axis) and by previous Funnell *et al.* analysis (y-axis) for every cell (dots), demonstrating an overall Spearman correlation of 0.95.

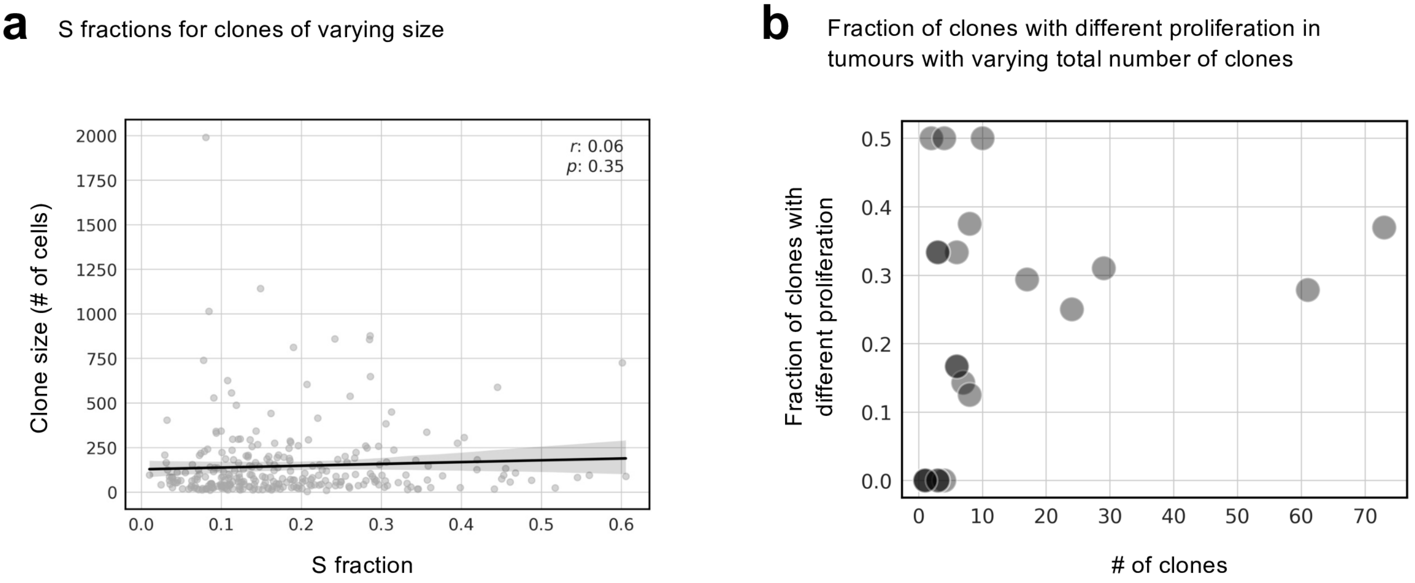

Supplementary Fig. 47: SPRINTER's results are not affected by clone size or number of clones. a, S fraction (x-axis) and clone size (number of cells per clone, y-axis) were measured for each clone (dots) identified across all patients in the previous TNBC and HGSC datasets, showing no clear correlation (Pearson correlation coefficient and test p-value are reported, and the 95% confidence interval for the linear regression is shown in shaded grey). **b,** Number of clones (x-axis) and fraction of clones with S fraction significantly different to the average per patient (tested using a Binomial test, y-axis) were measured for each patient in the previous TNBC and HGSC datasets (dots), showing no clear correlation.

a Deletions in TSGs associated with proliferation

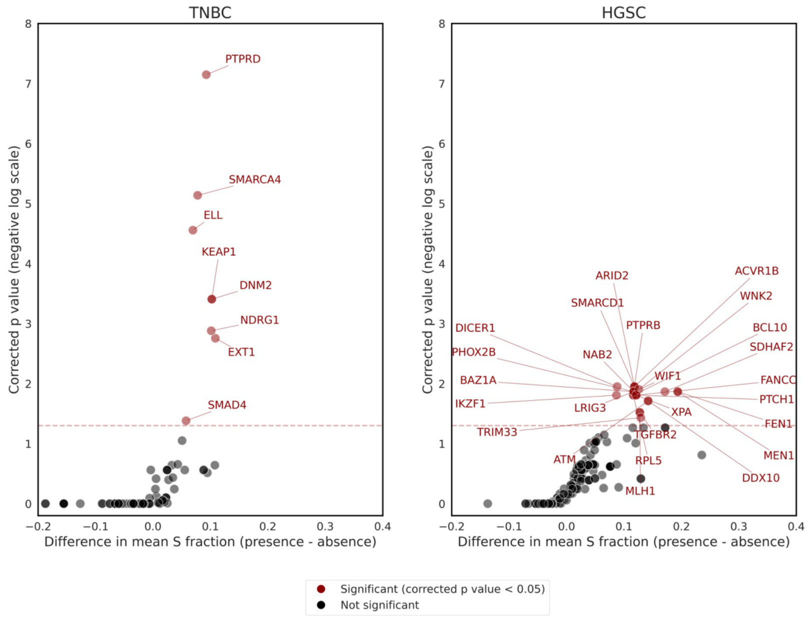

b Driver mutations associated with proliferation

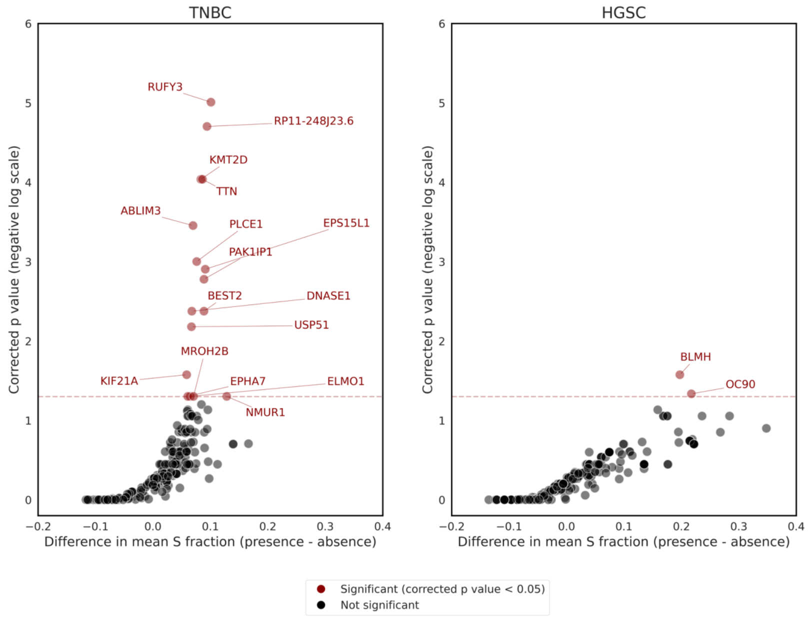

Supplementary Fig. 48: Driver mutations and deletions of tumour suppressor genes (TSGs) associated with high clone proliferation. a, For each known TSG (dots, obtained from the COSMIC Cancer Gene Census excluding oncogenes), a one-sided Mann-Whitney U test was performed between the S fractions of clones with or without a deletion of the TSG to determine a corrected p-value (y-axis, negative log scale), and the related difference between the average S fractions was calculated (x-axis). After applying the Benjamini-Hochberg multiple hypothesis correction (with family-wise error rate 0.05), genes passing the test (red with annotations, with the minimum corrected threshold indicated with the dotted line) are enriched in clones with increased proliferation. **b,** For each driver mutation (dots), a one-sided Mann-Whitney U test was performed between the S fractions of clones with or without the mutation, and the related difference between the average S fractions was calculated (x-axis). After applying the Benjamini-Hochberg multiple hypothesis correction, genes passing the test (red with annotations, with the minimum corrected threshold indicated with the dotted line) are enriched in clones with increased proliferation.

Supplementary Notes

1 Assessing the accuracy of S and G2 phase identification

We evaluated SPRINTER's accuracy in identifying replicating cells in the generated ground truth datasets. We found that SPRINTER outperformed two previously established methods, the cell cycle classifier (CCC)[1] and the MAPD method[2], in the identification of S phase cells in all generated diploid and tetraploid phases (Fig. 2a). We show similar improvements with SPRINTER even when compared to a version of MAPD that integrates replication timing (rtMAPD), demonstrating that just integrating reference replication scores in existing methods is not sufficient to provide the improved accuracy enabled by the novel features of SPRINTER (Fig. 2a). When each method was applied to 100 populations of 500 diploid or tetraploid cells sampled uniformly from each cell cycle phase, previous methods failed to identify cells in early and late S phase (0.2-20.5%). In contrast, SPRINTER enabled the identification of nearly all late S phase cells (>97.3%) and a fraction of early S phase cells (15.5-36.1%), while also improving the identification of mid S phase cells (>98.5% vs 39.4-90.3%) and maintaining high G1 accuracy (>94-99%, with SPRINTER's S phase calls in the G1 ground truth consistent with low infiltrating errors, Supplementary Fig. 12). Importantly, we found that SPRINTER's accuracy was robust to a higher fraction of replication-timing errors than the maximum expected in different normal and cancer cells (Supplementary Fig. 16) as well as to the use of different input subsets of replication scores obtained from both normal and cancer cell lines (Supplementary Fig. 17). These results demonstrate SPRINTER's robustness even in the presence of replication timing errors or alterations specific to the cells analysed. We further confirmed SPRINTER's improved accuracy using a previous phase-sorted scDNA-seq dataset[2] of 5,970 cells from the lymphoblastoid cell line GM12878 (Supplementary Fig. 14), demonstrating SPRINTER's applicability to different scDNA-seq platforms (i.e., 10x CNV Solution) and also confirming that these improvements are not specific to the newly generated ground truth datasets, but they extend to other datasets and scDNA-seq technologies. At the same time, previous methods displayed generally high accuracy in the identification of mid S phase cells, indicating that these methods remain useful in studies that mostly rely on the identification of these cells, such as replication timing studies[2]. Lastly, we found that SPRINTER accurately identified G2 cells (>80% precision and recall, Supplementary Fig. 18) and provided the best prediction of actively replicating (S and G2 phase) cells at all possible cut-offs of the inferred classification scores (Fig. 2b).

2 Assessing the impact of improved identification of replicating cells

We assessed whether SPRINTER enables the accurate identification of clones with the same or different proliferation rates. To do this, we used the ground truth datasets to generate 600 pairs of diploid and tetraploid cell subpopulations with varying numbers of cells (150-900), with varying fractions of actively replicating cells (20-30%), and with either the same or a different (±30-50% relative change) replicating cell fraction in the corresponding paired clone (Fig. 2c,d). We applied

every method to each pair to assess whether the inferred number of actively replicating cells (S and G2 phase) was sufficient to correctly classify if the pair had the same replicating cell fraction or not. We found that SPRINTER enabled the correct classification of most pairs with high accuracy (>85-95%, Fig. 2d). In contrast, previous methods only displayed limited accuracy of <70% for most pairs and even lower accuracy of <60% for most subpopulations with <450 cells (Fig. 2d). Given that the size of tested clones is compatible with those found in previous single-cell studies[1,3-5], SPRINTER's accuracy is thus required for accurate proliferation analyses of single-cell data. Furthermore, by applying all methods to an additional dataset of 4,163 diploid and tetraploid unsorted cells, we found that methods that aggregate all cells together for S phase identification, especially MAPD, failed to accurately identify S phase cells in heterogeneous samples containing cells with different ploidies (both diploid and tetraploid cells), in contrast to SPRINTER, which preserved its accuracy (Supplementary Fig. 15). This is because distinct subpopulations of cancer cells with different ploidies within the same sample might require the use of different thresholds for accurately classifying S phase cells. This result further highlights that it is not only the use of replication scores but also the other novel features of SPRINTER, especially the cell-specific test for S phase cells, that are key to enabling accurate S phase identification in heterogeneous cancer samples.

3 Assessing the clone assignment of S phase cells

We evaluated whether SPRINTER enables accurate clone assignment of S phase cells. Preliminary studies have attempted to overcome this challenge by using basic assignment heuristics based on correlation metrics[6] that do not account for replication fluctuations (Fig. 2e,f). To assess SPRINTER's novel clone assignment, we generated 30 subpopulations of 300 cells each, comprising altogether 389 clones from tetraploid ground truth cells, which contain clearly distinct clones (Supplementary Fig. 19) that are expected to have the same proliferation based on previous studies[7]. On each subpopulation, we applied both SPRINTER and previous heuristics, which involved inferring the same number of clones as SPRINTER using hierarchical clustering and assigning S phase cells identified by either CCC or MAPD to the clone with maximum correlation. We found that SPRINTER outperformed previous approaches (Fig. 2f) with significantly lower error rates per clone both in the fraction of true S phase cells (<12% vs. >35%, $p < 10^{-59}$ signed-rank test) and in the fraction of inferred S phase cells ($p < 10^{-53}$ signed-rank test, Supplementary Fig. 20). While previous methods obtained significantly different S fraction estimates than the expected values for >32% of clones, SPRINTER obtained consistent results for nearly all clones (>98%, Fig. 2f). Through an experiment involving the spike-in of CNAs in these ground truth datasets, we also showed that the accurate clone assignments provided by SPRINTER enable the recovery of most CNAs >3Mb in both S and non-S phase cells (Supplementary Fig. 21).

4 Consistency with previous bulk analysis of the NSCLC case

We evaluated the consistency of SPRINTER's clone inference on the NSCLC case with a previous bulk analysis of the same primary tumour, which included the same five samples and three

additional samples (Fig. 3d). SPRINTER's results on the TRACERx primary tumour samples were compared to previous bulk analyses[8,9]. To do this, we defined a distance based on SNVs to map each bulk clone identified in previous analyses to the closest single-cell clone inferred by SPRINTER. The distance between two clones was computed as the fraction of non-truncal SNVs uniquely present in only one clone over the total number of non-truncal SNVs. For this analysis, only SNVs that were identified as non-truncal in bulk analyses and were present in at least one bulk and one single-cell clone were used. Additionally, SNVs in single-cell clones were identified as present if they were regarded as either clonal or unknown according to the previous phylogenetic classification of SNVs as clonal, absent, or unknown (Supplementary Notes 25 and 26).

We found that the SNVs identified in SPRINTER-inferred clones were consistently most similar to those identified from the same sample in the previous bulk analysis (Supplementary Fig. 35). Similarly, the CNAs inferred by SPRINTER were found to be highly consistent with the CNAs inferred in the previous bulk analysis (Supplementary Fig. 36). We also matched each bulk clone to the most similar SPRINTER clone using SNVs: while bulk clones generally matched to single-cell clones identified in the same sample (Fig. 3d), we also found that several single-cell clones did not have a matched bulk clone, confirming the increased resolution of SPRINTER's single-cell analysis. Moreover, we observed that clones with higher proliferation did not always correspond to higher proportions of cells in the primary tumour, suggesting that these clones might have arisen later in time than those with lower proliferation (e.g., green clone in region 4 vs. burgundy clone in regions 1, 2, 6, and 8 in Fig. 3d).

5 Corroborating SPRINTER's estimates with Ki-67 analysis

We orthogonally validated SPRINTER's results using pathological analysis of Ki-67 staining on tumour areas adjacent to each sequenced sample. A Ki-67 score was measured for all primary and metastatic samples for which a slide of sufficient quality was available (i.e., sufficiently low necrosis and high tumour content, details in Supplementary Note 8), including primary region 4 and metastatic samples from the left adrenal, right adrenal, left frontal lobe, and liver (Fig. 3b and Supplementary Fig. 32). The Ki-67 and SPRINTER's estimates were nearly the same in primary region 4 (25% vs 23.5%) and, despite the distance between the Ki-67 and sequenced areas being larger in metastases, the estimates were overall consistent in the metastases as well (Supplementary Fig. 32a). Notably, the samples that could not be analysed using Ki-67 due to poor slide quality were successfully analysed using SPRINTER, highlighting a further advantage of the method. Lastly, Ki-67 analysis also corroborated the heterogeneous proliferation rates estimated by SPRINTER within the same sample in both the primary tumour and metastases: every analysed sample contained tumour areas within a slide with clearly different S fractions, with at least one area in each sample matching a clone estimate from SPRINTER (Fig. 3b and Supplementary Fig. 32b).

6 Corroborating SPRINTER's results with nuclear and clinical imaging

We further validated SPRINTER's results using DLP+ nuclear and patient clinical imaging. First, we measured the nuclear diameter of every sequenced cell, leveraging the nuclear images obtained as part of the DLP+ protocol[1]. As expected from previous studies[1] and from the generated ground truth datasets (Supplementary Fig. 34), we found that nuclear diameter significantly increased across the cell cycle phases identified by SPRINTER (Fig. 3c and Supplementary Fig. 33, $p < 10^{-10}$ Mann–Whitney U test). Even more importantly, we found a significant increase in diameter across the cell phases identified by SPRINTER per clone (Fig. 3c and Supplementary Fig. 33, $p < 0.0043$ signed-rank test). Second, we measured tumour growth rates using computed tomography (CT) and magnetic resonance (MR) imaging acquired for five serial time points through the patient's metastatic disease course collected during routine clinical management and collected as part of the TRACERx study (Supplementary Note 9). Consistent with the expectation that higher proliferation results in increasing disease burden, we found the average growth rates of the left adrenal, left frontal lobe, right adrenal, and right occipital lobe metastases (3.34, 1.94, 1.1, and 0.43 log(mm^3/day) in Extended Data Fig. 6) ranked metastases in the same order as the average S fractions estimated by SPRINTER (40.9%, 37.1%, 30.8%, and 13.4% in Fig. 3a). Additionally, these results confirm that increased sample S fractions relate to increased proliferation, rather than changes in the length of cell cycle phases.

7 TRACERx and PEACE tumour tissue samples of a patient with NSCLC

The five primary tumour samples and five anatomically distinct metastatic samples analysed in this study have been obtained from patient CRUKP9145 (equivalent to patient CRUK0516) with NSCLC from the TRACERx study[8,9] (https://clinicaltrials.gov/ct2/show/NCT01888601, approved by an independent Research Ethics Committee, 13/LO/1546) and the PEACE autopsy study[10] (https://clinicaltrials.gov/ct2/show/NCT03004755, approved by an independent Research Ethics Committee, 13/LO/0972). Informed consent was obtained as part of these studies. The patient was a 60-year-old male with stage IIIA squamous cell carcinoma, who underwent surgical removal of the primary tumour and who subsequently relapsed and died 251 days later after receiving multiple lines of chemotherapy and radiotherapy (Supplementary Fig. 22). The patient died with metastases in multiple anatomical sites and was enrolled in the PEACE autopsy programme, through which a post-mortem examination was performed.

Samples for single-cell sequencing were taken from five anatomically distinct samples from the primary tumour removed at surgery (regions 2, 3, 4, 5, and 8) and five different metastases sampled at autopsy (left adrenal, right adrenal, liver, left frontal lobe, and right occipital lobe) (Supplementary Figs. 24 and 25). All surgically resected primary tumour samples were macroscopically reviewed by a pathologist. Spatially separated tumour samples were collected, photographed, and snap frozen in liquid nitrogen for subsequent sequencing. Tissue sampling at autopsy occurred as soon as possible following patient death (63 hours after death) and was led by a pathologist, guided by information on the patient's clinical history and pre-mortem radiological

imaging results. The analysed metastases were sampled and annotated with an anatomical description by the attending pathologist. Each metastasis sample was bisected along the long axis and one half immediately snap-frozen in liquid nitrogen before long-term storage at −80°C, with the other half fixed in 10% neutral buffered formalin prior to embedding in paraffin blocks (formalin-fixed paraffin-embedded, FFPE) before storage at room temperature. In order to avoid cross-contamination, fresh instruments were used to handle each individual tumour. Moreover, the primary tumour biopsy was tested using immunohistochemistry to determine the percentage of tumour cells expressing PDL1 as part of standard clinical care (using the PDL1 IHC 22C3 Dako PharmDx assay), and this information was collected as part of the TRACERx trial.

8 Pathological assessment of Ki-67

Ki-67 immunohistochemistry was performed on sections taken from FFPE tissue blocks created from the five primary and five metastatic samples analysed in this study from patient CRUKP9145 with NSCLC. Immunohistochemistry was performed using a Bond-III Autostainer (Leica Biosystems) according to the manufacturer's instructions. The FFPE samples tested were taken directly adjacent to the frozen area that was sequenced. Due to the larger size of the metastatic lesions and their division into FFPE and frozen sections as described above, slides for metastases tended to have a greater distance from the sequenced area than that seen for primary tumour samples. Slides with high levels of necrosis or low purity which prohibited accurate Ki-67 measurement were discarded for further pathological analysis (including primary regions 2, 3, 5, and 8, and the metastatic right occipital lobe sample). Fractions of positively stained cancer cells were scored by a pathologist in line with clinical guidelines to determine average Ki-67 scores per sample and within-sample Ki-67 scores.

9 Patient clinical imaging analysis

For patient CRUKP9145 with NSCLC, pseudoanonymised imaging obtained in routine clinical follow-up during the metastatic disease course (from disease relapse through to the last scan before death) was collected as part of the TRACERx study[9] (Extended Data Fig. 6). 3D tumour volumes of all measurable metastases with matched single-cell sequencing data were contoured using the software ITK-SNAP[11] by an oncology clinician and reviewed by a consultant clinical oncologist. Linear regression lines were calculated and plotted across lesion-specific volumes for all imaging timepoints. The gradient of the plotted regression line represented the change in lesion volume over time (in mm^3). Finally, the log change in tumour volume over time ($\log10(mm^3)$) was used to capture the average lesion growth rate from lesion detection to death.

10 Classification of replication timing for genomic regions

Cells replicate their DNA in a specific pre-defined order, which has been shown to be highly conserved across different cells, tissue types[12,13], and even cancer cells[12-16]. In fact, >50% of the

genome has preserved early or late replication timing across different cell types and cancers (Supplementary Fig. 1). For the identification and copy-number analysis of S phase cells, SPRINTER classifies and uses only genomic bins that are expected to have conserved early or late replication timing, while all genomic bins are used to perform the other steps of SPRINTER. To identify early and late genomic bins, we use reference replication profiles obtained from experimental Repli-Seq data[17] from different normal and cancer cell lines. Specifically, each profile provides a replication score for each genomic region (in the form of a log2 ratio), with higher positive values indicating earlier replication timing and lower negative values indicating later replication timing. In this study, we have collected three distinct sets of reference replication profiles obtained by analysing Repli-Seq data in previous studies:

(i) Previously generated datasets for lung adenocarcinoma (A549, H1650, H1792, and H2009) and normal cell of origin (TT1 and pulmonary alveolar epithelial type II cells i.e., T2P) cell lines[18];
(ii) Previously generated datasets for breast cancer (T47D, MDA453, SK-BR3, and MCF-7) and normal cell of origin (HMEC and MCF10A) cell lines[18];
(iii) A range of publicly available cancer and normal cell lines from the ENCODE database[19] (A549, BG02, BJ, Caki2, HUVEC, G401, H460, HeLa-S3, HepG2, IMR90, keratinocyte, LNCAP, SK-N-MC, and SK-N-SH).

In addition, in this study we used an additional set of replication profiles generated using the Repli-Seq protocol[17] as part of previous studies[18] for three lung squamous cell carcinoma cell lines (H520, H2170, SW900) and a corresponding normal cell of origin cell line (HBEC3). As such, we applied SPRINTER using three different subsets of these Repli-Seq datasets to obtain the related replication profiles:

(i) A set of normal lung and breast cell lines all generated using the same sequencing platform and protocol (TT1, T2P, HBEC3, HMEC, and MCF10A);
(ii) A set of all normal cell lines also including ENCODE data (TT1, T2P, HBEC3, HMEC, MCF10A, BG02, BJ, HUVEC, IMR90, and keratinocyte);
(iii) All available normal and cancer cell lines listed above.

Given a subset of replication profiles, SPRINTER uses the related replication scores to classify the replication timing of different genomic regions. Specifically, for each genomic bin (50kb by default), SPRINTER calculates the average score across the input subset of reference replication profiles. Since the average replication scores form a bimodal distribution (Supplementary Fig. 1), we use fixed thresholds (i.e., <-0.5 for late bins and >0.5 for early bins by default) to classify each bin as either early replicating, late replicating, or unknown. Importantly, we found that the classification of these bins is mostly conserved when using different subsets of reference replication profiles, which were also generated by different Repli-Seq experiments (Supplementary Fig. 1). Moreover, we found that these classifications were not substantially affected by the use of more stringent definitions: for example, we observed that the replication timing classifications were mostly conserved when each bin was defined as either early or late replicating only if the bin had the same classification across nearly all (>95%) of the cell lines included in the chosen subset of replication profiles.

Overall, SPRINTER classifies >85% of the genome as early or late when considering the default classifications, and >50-70% when considering the more stringent definitions described above. By default, SPRINTER uses the subset of reference replication profiles obtained from the normal lung and breast cell lines (TT1, T2P, HBEC3, HMEC, and MCF10A) that were generated within the same study to minimise batch effects in the estimated replication scores (since these cell lines were sequenced with the same sequencing platform and protocol) and the impact of cancer-specific replication timing alterations. However, we found that using different input subsets of the replication scores does not affect SPRINTER's results (Supplementary Fig. 17).

11 Calculation of read depth ratios

Read depth ratios (RDRs) are the main sequencing signal used across all steps of the SPRINTER algorithm. Specifically, RDRs are used in each cell to capture read-count fluctuations that are induced by either CNAs or replication. Similar to previous single-cell studies of CNAs[5], SPRINTER partitions the reference genome into m small genomic bins (50kb by default, the same defined in Supplementary Note 10). The RDR of each genomic bin is computed from DNA sequencing data by comparing the observed number of sequencing reads aligned to that genomic region with the expected number of aligned sequencing reads for the same genomic region calculated using a control. As such, SPRINTER computes the RDR x_b of each bin b in three steps for each cell independently.

In the first step, SPRINTER aggregates the read counts of neighbouring bins into larger windows, since small bins may not have a sufficiently high number of reads to provide accurate estimates of RDRs due to the low sequencing coverage of scDNA-seq. As such, for each cell separately, SPRINTER splits the reference genome into sliding windows of cell-specific size w, such that each bin represents the start of a different window. In contrast to previous methods[1-3,5,6], w is chosen independently for each cell in order to obtain a fixed number of reads on average M across all windows. This step is important because the total number of sequencing reads per cell R varies across different cells using scDNA-seq technologies (especially those based on tagmentation[1,4]) according to their total DNA content; for example, G2 cells are expected to yield a higher total number of reads than G1 cells (Supplementary Fig. 8). Therefore, the choice of a fixed average number of reads across windows allows SPRINTER to estimate RDRs with the same expected variance, which is important for comparative analyses across cells. Specifically, SPRINTER defines w as the number of bins to be aggregated within the same window, calculated as $w = \left\lfloor {}^{M}/_{R/m} \right\rceil$ for each cell, and the number of reads r_b to be considered for each bin b of the cell is calculated by aggregating the read counts for all bins within the corresponding window. Moreover, SPRINTER defines windows of different sizes for two of the key steps: smaller windows are used for the identification of S phase cells ($M = 200$ in this study), and larger windows are used for the inference of CNAs ($M = 2000$ in this study). Furthermore, during the identification of S phase cells, SPRINTER defines the windows in order to only combine genomic bins with the same early or late replication timing. Therefore, during the identification of S phase cells and based on the classification of early and late bins described above (Supplementary Note 10), SPRINTER defines windows by combining

bins with the same replication timing when they are neighbouring in the reference genome or if they are sufficiently close (<150kb by default).

In the second step, SPRINTER calculates the control number of sequencing reads $\tilde{r}_b$ for each bin b by considering the same windows defined in the first step. Previous studies calculated $\tilde{r}_b$ by counting the number of aligned reads in b by using a matched normal sample or by using normal cells as a control[5]. However, these approaches rely on sequencing a sufficiently high number of normal cells, which cannot be guaranteed when sequencing tumour tissues. Therefore, like previous studies[1], SPRINTER calculates $\tilde{r}_b$ for all bins by using available computational methods (e.g., the ART algorithm[20]) to simulate DNA sequencing reads with the same sequencing features as those of the sequenced cells (i.e., DNA library size, insert size, etc.) and by re-aligning the sequencing reads to the reference genome using the same procedure (e.g., using BWA for re-alignment to the human reference genome) as the analysed cells (to guarantee the same alignment biases). Note that different sequencing error profiles of the simulated reads are not expected to generate significantly different sequencing read counts when considering genomic bins larger than hundreds of thousands of base pairs, as in this study.

In the last step, SPRINTER obtains the RDR x_b of each bin b using the calculated read counts r_b in the sequenced cells and $\tilde{r}_b$ in the simulated control by applying the same approach as in the existing CHISEL algorithm[5]. Specifically, SPRINTER calculates x_b of each bin b as follows

$$x_b = \frac{r_b}{\tilde{r}_b} \cdot \frac{\tilde{R}}{R}$$

where $R, \tilde{R}$ represent the total number of sequencing reads for the corresponding cells and the total number of control reads, respectively.

12 Replication-aware correction of GC bias

SPRINTER introduces a replication-aware method to correct RDRs for GC sequencing bias[21,22], incorporating the classification of replication timing in order to preserve RDR fluctuations induced by replication in S phase cells. In particular, GC bias is a typical bias in DNA sequencing experiments that affects calculated RDRs[21,22] by introducing variations in the RDRs of genomic bins with different GC content (i.e., fraction of GC nucleotides) that are not due to any underlying difference in the sequenced genome[22]. Previous methods correct GC bias in RDRs by fitting a function that models the relationship between RDRs and GC content (e.g., using linear models or local regressions[1,3,21,22]). Although these approaches have proven to be successful in enabling accurate CNA identification, they also lead to the erroneous correction of RDR fluctuations induced by replication in S phase cells (Supplementary Fig. 5). In fact, since early replicating genomic regions are GC enriched and late replicating regions are GC depleted, replication-induced fluctuations are identified as GC bias and erroneously corrected, discarding the main signal used to identify S phase cells.

To overcome this limitation, SPRINTER introduces a replication-aware correction of RDRs for GC bias by leveraging two key observations. First, groups of bins with the same replication timing (early or late) are less affected by fluctuations induced by replication as they replicate at more similar times. Thus, SPRINTER infers GC biases in early and late bins separately using a quantile linear regression. Second, bins with higher GC content tend to replicate earlier than bins with lower GC content and their RDRs increase during S phase (Supplementary Fig. 5). Thus, SPRINTER identifies the inferred regressions that are still affected by GC bias as those with an inferred slope substantially higher than other cells and corrects them. Below, we describe the details of this approach for correcting GC sequencing bias while preserving replication fluctuations.

The main idea underlying SPRINTER's GC correction approach is to infer the GC correction independently within groups of bins that are either early or late replicating. Since bins within the same replication group replicate their DNA at more similar times than bins in different groups, the RDRs of bins within each of these groups are less affected by replication fluctuations, and, therefore, GC correction can be estimated more accurately. Similar to previous studies[1,3], we use a linear model for estimating the GC bias within each replication group by comparing RDRs and GC content across bins. Intuitively, a linear slope of 0 is expected when there is no GC bias and, conversely, varying values of the linear slope would indicate the presence of different GC biases. To improve the robustness of the regression and overcome the effect of further variations within each replication group (e.g., due to differences between replicated/unreplicated bins within the same group), we use a quantile linear regression using the median as quantile (which is less affected by the presence of outliers and other variations than standard linear regression), fitting the model using existing standard algorithms for quantile regressions[23]. For each cell independently, we thus estimate the GC bias within each replication group (i.e., early replicating bins, late replicating bins, and all bins together) and we combine these estimates by averaging the inferred linear slopes and intercepts.

Despite the approach proposed above, the inferred linear model of GC bias can still be affected by replication due to the presence of some replicated and unreplicated bins within each replication group (i.e., within early or late groups of bins). However, we can predict the effect of replication on the inferred linear slopes: replication induces higher values of the inferred linear slopes because bins with higher GC content tend to replicate earlier than bins with lower GC content, inducing higher values of RDRs for higher GC content bins and, thus, higher linear slopes (Supplementary Fig. 6). Based on this observation, we apply an approach that aggregates information across all cells sequenced together to identify the inferred slopes still affected by replication, since cells sequenced with the same DNA sequencing library are expected to have similar GC biases. Specifically, we model the distribution of inferred linear slopes for GC bias as a Normal mixture model with two components, such that the largest component with lower inferred GC slopes is used as a reference and the minor component with higher inferred GC slopes is defined as the outlying distribution, composed of inferred slopes still affected by replication. As such, within each replication group, we infer the GC linear slope by preserving the inferred value if this falls within the 95% confidence interval of the reference distribution, or otherwise correcting it to the mean of the reference distributions.

Lastly, different datasets generated with different DNA library preparation procedures can result in substantially different GC biases in the DNA sequencing data. In datasets affected by strong GC bias (e.g., high inferred GC linear slopes >6 as found in some previous datasets[3]), accurate GC correction is more sensitive to errors or underestimations. Therefore, in these datasets we adopt the same approach to estimate GC bias, but with more stringent and conservative statistics. Specifically, we estimate the GC linear slope per cell by taking the maximum rather than the average across different replication groups when the median inferred value of GC slopes is high (i.e., >2 by default) in any replication group for non-outliers.

13 Replication-aware copy-number segmentation

In SPRINTER, we developed a replication-aware algorithm for copy-number segmentation, which is applied to each cell independently to identify segments of neighbouring bins that are likely affected by the same CNAs. The algorithm uses in input the RDR x_b computed for each bin b and a bipartition of the selected genomic bins into two replication groups, defined as two sets $E \subset \{1, \dots, m\}$ and $L \subset \{1, \dots, m\}$ containing the bins with either early or late replication timing (as defined in Supplementary Note 10). As such, the algorithm is composed of two steps: the first identifies candidate breakpoints in each replication group independently, and the second infers segments by only selecting the breakpoints that are compatible with CNAs. These two steps are detailed below.

The first step infers candidate breakpoints in each replication group independently by identifying change points in RDRs using a Hidden Markov Model (HMM) to capture the dependency across neighbouring genomic bins as done in previous studies[1]. In particular, we model RDRs as a mixture of k Normal distributions, such that the RDR x_b of each bin b is modelled as a draw from a Normal distribution with a mean u, which is proportional to the underlying copy number c_b and replication state t_b, and standard deviation σ. Since the emissions of an HMM model must be independent, we only use RDRs estimated from non-overlapping windows of bins (see Supplementary Note 11) in this model by considering the first bin of each window as representative, with the other bins overlapping the window assigned to the same state as this representative bin after the inference has been made. As such, given a possible value of k, the maximum likelihood estimates of u and σ for each of the k mixture components are estimated using the standard Baum–Welch algorithm for HMMs and the most likely HMM state of each bin (i.e., assignment to one of the components) is inferred using the standard Viterbi algorithm. Since the value of k is unknown and higher values of k generally lead to solutions with higher likelihood but also higher model complexity, we repeat the process with varying values of k (from 2 to 12 by default) and the best value is chosen using the standard Bayesian Information Criterion (BIC) to balance the choice between fit of the data and model complexity. In each replicating group, we thus define the candidate breakpoints for the segments as pairs of neighbouring bins with different inferred HMM states (with bins inferred with the same HMM state being those assigned to the same mixture component that are thus inferred to have the same underlying values of copy number c_b and replication state t_b). Specifically, given the number of components k^E and k^L inferred in the early

and late replication groups, we use two integer variables $h_b^E \in \{1, \dots, k^E\}$ and $h_b^L \in \{1, \dots, k^L\}$ to represent the inferred HMM state for each bin b when $b \in E$ or $b \in L$, respectively.

The second step infers copy-number segments by selecting and preserving only those segments that are likely to have been induced by CNAs. In fact, the observed RDR x_b of each bin b is expected to be directly proportional to both the copy number c_b and replication state t_b, i.e., $u \propto c_b \cdot t_b$. As such, RDR fluctuations across the genome can be induced by either CNAs (changes in the copy numbers c_b) or by replication in S phase cells (changes in the replication state t_b). By separately inferring breakpoints in either the early or late group, we expect that most of the identified breakpoints are induced by CNAs, since most of the breakpoints induced by replication occur between early and late bins and are thus discarded by considering early and late groups separately. However, changes in the inferred HMM states of neighbouring bins can still be induced by either CNAs (changes in the copy numbers c_b) or by replication (changes in the replication state t_b) since bins within the same early or late group can still replicate at different times in S phase cells (i.e., they can still have slightly different values of the replication state t_b, Extended Data Fig. 3 and Supplementary Figs. 11-13). We use the expected differences in the size of the genomic regions affected by these changes to distinguish these two remaining cases: while segments induced by replication tend to be short (since consecutive genomic regions with the same replication timing are short, i.e., <1Mb on average with a median of 250kb, Supplementary Figs. 1-3), CNAs measured in single-cell studies affect large genomic segments[24] (i.e., ~42Mb on average with >99.9% of CNA segments >2Mb in size, as measured in previous single-cell studies[3], Supplementary Fig. 2) and are hence expected to induce segments containing both early and late replicating bins. Inspired by this principle, we thus introduce an approach to combine the breakpoints that were inferred separately in each group and then select only the corresponding segments that are supported by breakpoints in both replication groups.

We first combine the breakpoints obtained in the two replication groups and obtain candidate segments as sequences of neighbouring bins that are not separated by any breakpoint previously identified in either the early or late replication group. As a result, we obtain $\widehat{m}$ candidate segments such that each segment s is composed of early and late bins that have been inferred to have the same HMM state, i.e., $s \subset E \cup L$ such that $\left|\{h_b^E : b \in s, b \in E\}\right| = 1$ and $\left|\{h_b^L : b \in s, b \in L\}\right| = 1$. We define the inferred HMM state for each segment as the pair (h_s^E, h_s^L) where $h_s^E = h_b^E$ for any bin $b \in s \cap E$ and $h_s^L = h_b^L$ for any bin $b \in s \cap L$. However, not every segment necessarily includes both early and late replicating bins since the breakpoints have been inferred between segments that might not be adjacent in the reference genome. For example, if five neighbouring genomic bins $\{1, \dots, 5\}$ with $\{1, 5\} \in E$ and $\{2, 3, 4\} \in L$ are inferred with the following HMM states $(1^E, 1^L, 2^L, 2^L, 2^E)$, a segment will be obtained such that it only includes the late bins 3 and 4 with HMM state $h_s^L = 2^L$ and with no early bin to define h_s^E. When this case occurs for a segment, we assign the missing early/late HMM state for the segment based on the next or previous neighbouring segment with a matching late/early HMM state, respectively (if this does not exist, the segment is discarded). As such, each segment s is assigned with an inferred HMM state (h_s^E, h_s^L).

Lastly, we use the inferred HMM states for all segments to select and preserve only the segments supported by breakpoints in both replication groups. Since most CNAs tend to affect large genomic regions, we expect that a segment defined by CNAs will induce changes in the HMM states of both early and late bins compared to the two neighbouring segments. For example, if segment s is induced by CNAs, we expect both that $h_s^E \neq h_{s-1}^E$ or $h_s^E \neq h_{s+1}^E$ and that $h_s^L \neq h_{s-1}^L$ or $h_s^L \neq h_{s+1}^L$. Conversely, segments induced by replication within the same replication group are expected to display differences only in either the early or late HMM state, since breakpoints were inferred in the replication groups independently (such that replication-induced differences between early and late regions were not identified as breakpoints). Segments induced by replication are thus not expected between different replication groups and are only expected within either the early or late replicating groups (due to the presence of bins that replicate earlier than other bins in the same group). Specifically, segments induced by replication are expected within the early group in early S phase, while segments induced by replication are expected within the late group in late S phase (Extended Data Fig. 3 and Supplementary Figs. 11-13). As such, we identify segments induced by replication as those segments that do not display changes in one of either the early or late HMM states compared to the two neighbouring segments. More specifically, we define a segment s to be induced by replication either if $h_s^E = h_{s-1}^E$ and $h_s^E = h_{s+1}^E$, or if $h_s^L = h_{s-1}^L$ and $h_s^L = h_{s+1}^L$. Therefore, we remove the segments identified as replication-induced, and, by removing the related breakpoints, the corresponding bins are included in the neighbouring segments with matching early or late inferred HMM states. The only exception to this selection is the presence of neighbouring segments that have been identified to be induced by replication using two different replication groups. For example, given four segments with the following HMM states $((1,1),(1,2),(1,2),(2,2))$, both the second and third segments are defined to be induced by replication, but the second is defined based on early HMM states (first element of pairs), while the third is defined based on late HMM states (second element of pairs). Since these cases are unexpected but can occur when CNAs affect genomic regions genomically close to segments induced by replication, these segments are not removed but are preserved by SPRINTER as segments induced by CNAs.

To conclude, we also note that rare CNAs that exclusively overlap large domains of early and late replicating regions can induce segments that cannot be identified with the approach described in this section. However, these segments can be correctly recovered in later SPRINTER steps. Specifically, in G0/1/2 phase cells all CNAs can be accurately inferred during the CNA identification step (Supplementary Note 15), since all RDR fluctuations can be related to CNAs in these cells, and the segments are re-inferred from all RDR values of all bins. Moreover, in S phase cells these rare CNAs can be later corrected using the CNAs inferred for the G0/1/2 phase cells assigned to the same clone (Supplementary Note 19).

14 Cell-specific identification of S phase

For S phase identification, SPRINTER leverages the expected RDR fluctuations between early and late replicating bins to introduce a statistical permutation test that can be applied to each cell independently. Specifically, we model the RDR x_b of bin b in a cell to be directly proportional to

the total copy number $c_b \in \mathbb{N}$ and the replication state $t_b \in [1, 2]$, which represents the replicated or unreplicated status of b in the actual phase of the cell, i.e., $x_b \propto t_b \cdot c_b$. Note that t_b has been modelled as a continuous variable within $[1, 2]$ rather than a discrete variable within $\{1, 2\}$ to account for the fact that each bin can potentially contain genomic regions with different replication timing. As such, we expect $t_b = 1$ for all bins in G0/1 phase cells, $t_b = 2$ for all bins in G2 phase cells, and both $t_b = 1$ and $t_b = 2$ for non-empty subsets of bins in S phase cells. While S phase cells can be identified as those cells with varying values of t_b across bins, these variations cannot be directly identified from RDRs since each x_b is also influenced by different copy numbers c_b. However, SPRINTER has already identified segments of bins likely affected by the same CNAs and, hence, with the same values of c_b (Supplementary Note 13). We thus leverage SPRINTER's copy-number segments to correct RDRs for the effect of varying values of c_b. To do this, we calculate the replication timing profile (RTP) $\tilde{x}_b \in \mathbb{R}$ for each bin b by normalising x_b by the median value of RDRs computed across all bins in the same segment (Extended Data Fig. 4 and Supplementary Fig. 7); as such, we expect that $\tilde{x}_b$ only depends on t_b, i.e., $\tilde{x}_b \propto t_b$. To avoid biased estimates due to varying numbers of early and late bins in different segments, we estimate the median RDR in each segment by bootstrapping the same number of early and late bins (using the minimum sample size between the two groups).

The resulting RTPs provide a signal to identify S phase cells since the RTP $\tilde{x}_b$ of each bin b only depends on the replication state t_b, and varying values of t_b across the genome are a hallmark of S phase cells. Although the values of t_b are unknown, we expect early bins to replicate before late bins (Extended Data Figs. 1-3 and Supplementary Figs. 11-13) and, hence, we expect in S phase cells that a subset of early replicating bins always have higher values of t_b than a subset of late replicating bins across the genome (i.e., $t_{b_E} \geq t_{b_L}$ for an early bin b_E and a late bin b_L). Specifically, S phase cells in the early stages of replication are expected to have a subset of early bins with higher t_b than all late bins (since none of the late bins have started replication), while S phase cells in the later stages of replication are expected to have a subset of late bins with lower t_b than all early bins (since all early bins have already replicated, Extended Data Fig. 3 and Supplementary Figs. 11-13). In order to use the RTPs to capture these two cases, we introduce the following two test statistics

$$\frac{|\{b : b \in E, \tilde{x}_b > L(1-q)\}|}{|L|} \quad \text{and} \quad \frac{|\{b : b \in L, \tilde{x}_b < E(q)\}|}{|E|}$$

where E, L are the sets of all early or late bins, $L(1-q)$ is the $1-q$ quantile for the RTPs of late bins, and $E(q)$ is the q quantile for the RTPs of early bins. In particular, $L(1-q)$ and $E(q)$ are used as reference RTP values to count how many early and late bins have higher and lower values, respectively, than those with a different replication timing, using a user defined value q to control sensitivity ($q = 0.05$ by default). Note that this statistic is expected to be robust to the presence of alterations or errors in replication timing classifications, since it requires only a subset of bins, not all early or late bins, to display the expected signal in RTPs.

To identify S phase cells, SPRINTER introduces a statistical permutation test to assess if either of these two statistics has a value significantly different than what is expected by chance in each cell, indicating the presence of a significantly large subset of early bins with substantially higher

RTPs than late bins, or a significantly large subset of late bins with substantially lower RTPs than early bins. Specifically, SPRINTER performs a permutation test by randomly permuting the early and late replication timing of bins (by default 10^5 permutations) and computing the null distributions of both statistics. Since variations of t_b are expected to occur along the entire genome during S phase, we perform the test on each chromosome independently and the resulting values of each statistic are combined using the harmonic mean; this approach helps overcome noise and errors that can be localised to certain genomic regions. A p-value is thus computed by fitting a Beta distribution to the empirical null distribution generated by the test, and the two p-values obtained for each statistic are combined using the minimum. Finally, a multiple-hypothesis correction is applied for all cells using the Holm–Šidák method and each cell that passes the correction is defined as S phase. In contrast to previous approaches that aggregate all sequenced cells together, SPRINTER's method is applied to each cell independently, providing a significance assessment for each cell individually and making the method suitable to heterogeneous tumour samples characterised by cells with different ploidies and CNA rates.

15 Inference of baseline copy numbers in G0/1/2 phase cells

SPRINTER infers CNAs in G0/1/2 phase cells by identifying the baseline copy number c_b of every bin b in each of these cells. In particular, we consider c_b as the baseline number of copies that the cell has in its corresponding G0/1 phase. This definition is necessary because while G2 cells have a doubled value of c_b for all bins, RDRs cannot be used to unequivocally distinguish the underlying copy numbers of cells with the same CNAs that are either in G0/1 or G2 phase. In fact, the RDR x_b of every bin b is linearly proportional to the underlying number of copies $\dot{c}_b$ according to a cell-specific scale factor γ that depends on the cell ploidy, i.e., $x_b = \gamma \cdot \dot{c}_b$ for every bin b. Therefore, since the cell ploidy is unknown, there always exists at least two values of γ that equivalently explain the same expected values of x_b, one for G1 and one halved for G2. Moreover, inferring baseline copy numbers is an essential feature for clone inference since clones are only distinguished by different, inherited CNAs and not by temporary variations of the underlying copy numbers induced by G1 to G2 by replication.

Like previous studies[1,5], we adopt a parsimonious approach that finds the value of γ yielding the lowest values of baseline copy numbers c_b to explain the observed RDRs. Specifically, we use a Hidden Markov Model (HMM) to assess the likelihood of multiple plausible values of γ and we choose the best candidate using the Bayesian Information Criterion to select among models of varying complexity (corresponding to higher copy numbers). To obtain a model which is more specific and robust than previous HMM algorithms[1,3], we also integrate the segments previously inferred by the second step of SPRINTER to fix most of the model parameters (e.g., RDR variance and CNA rates, Supplementary Note 13). Note that CNA segments are re-inferred directly from RDRs in this step for G0/1/2 phase cells using all genomic bins and considering every potential RDR fluctuation since G0/1/2 phase cells are not affected by replication fluctuations. Therefore, rare CNA segments that might have been erroneously excluded from the second step of SPRINTER because they occur in genomic regions with only early or late replication timing can be correctly recovered

in this step and can also be used later to correct potentially missed CNAs in S phase cells assigned to the same clone.

In detail, SPRINTER infers the baseline copy number c_b of every bin b in every G0/1/2 phase cell in two steps: the first step identifies the scale factor γ, relating c_b to the observed RDR x_b such that $c_b = \gamma \cdot x_b$ as shown in previous studies[5], and the second step infers CNA segments and the corresponding copy numbers. Moreover, the RDRs estimated in larger windows and using all genomic bins (Supplementary Note 11) are used for this step (as well as subsequent steps related to CNAs) since most CNAs affect large genomic regions (e.g., >2Mb[1,3,5,25], Supplementary Fig. 2). Below, we describe the details of these two steps.

First, to identify the scale factor γ, SPRINTER evaluates a set of plausible values for γ and chooses the best candidate using model selection. In particular, SPRINTER enumerates plausible values of γ by guessing all realistic values of copy numbers c_b (i.e., 2, 3, 4, …) for all the bins belonging to the most frequent copy-number state, which was identified during the preliminary segmentation performed in the previous second step of SPRINTER (Supplementary Note 13). Specifically, similar to previous studies[5], the set Γ of plausible values of γ is obtained as

$$\Gamma = \{\frac{1}{|B|} \sum_{b \in \mathrm{B}} x_b / _{\theta} \, : \, \theta \in \{2, \dots, \breve{\theta}\}\}$$

where B is the largest set of bins identified with the same copy-number state by SPRINTER's segmentation and $\breve{\theta}$ is the fixed maximum value for the copy number of bins in B (by default $\breve{\theta} = 4$). As such, SPRINTER evaluates each candidate $\gamma \in \Gamma$ by computing the likelihood $\mathcal{L}(\gamma; x_1, \dots, x_m)$ using an HMM with Normal emissions and a diagonal covariance matrix as described in the next step.

Next, SPRINTER aims to identify CNA segments and the corresponding copy numbers by evaluating each candidate value of the scaling factor γ identified earlier and using an HMM (with a model different than the one applied in Supplementary Note 13). While HMMs have been used in previous methods for single-cell CNA inference[1,3], SPRINTER introduces a simpler model to improve robustness (i.e., fewer parameters) by integrating additional information separately inferred in previous SPRINTER steps. In particular, SPRINTER fixes three main parameters: (1) the means of the Normal distributions are fixed to the expected RDR values for all possible copy numbers, which can be directly obtained from the given γ as $\{\gamma \cdot c : c \in \{0, \dots, \lfloor \gamma \cdot \max_b x_b \rceil\}\}$; (2) the variances of the Normal distributions are fixed using a sample estimate computed from the segments previously inferred in the second step of SPRINTER; and (3) the probability of changing each copy-number state in the transition matrix is fixed to a constant based on the number of previously inferred segments. Moreover, similar to previous SPRINTER steps (Supplementary Note 13), only non-overlapping windows are used as emissions of the HMM model. Thus, $\mathcal{L}(\gamma; x_1, \dots, x_m)$ is computed by only fitting the starting probabilities (i.e., the probability of the first copy-number state in each chromosome) using the standard Baum–Welch algorithm, and the corresponding baseline copy numbers $c_1, \dots, c_m$ are inferred using the standard Viterbi algorithm. Since higher values of γ tend to lead to higher likelihood values but also higher model complexity (i.e., higher number of parameters increasing

with the number of available copy numbers), SPRINTER chooses the best value of γ and corresponding baseline copy numbers $c_1, \dots, c_m$ using the standard model-selection BIC as in previous steps.

16 Inference of CNA-based clones

SPRINTER infers clones by identifying distinct subpopulations of cells that share the same complement of CNAs, which have been previously inferred by SPRINTER for all G0/1/2 phase cells (Supplementary Note 15). This inference is not straightforward because cells in the same clone are not expected to have identical CNAs due to the presence of cell-specific CNAs and errors in the inferred CNAs. While the previous CHISEL algorithm has introduced a hierarchical clustering approach to overcome these challenges[5], this approach relies on fixed and known estimates of the distance between cells and related clones, namely the cell-to-clone distance computed using the Hamming distance of the inferred CNAs. Briefly, given a known estimate of the maximum cell-to-clone distance ϵ between two cells in the same clone, CHISEL infers the clones by cutting the reconstructed hierarchy into groups of cells that have pairwise distances lower than ϵ. However, the rates of cell-unique CNAs and errors can vary substantially in different datasets and across different scDNA-seq technologies. Therefore, SPRINTER improves CHISEL's approach by introducing an auto-tuning procedure to automatically estimate the maximum cell-to-clone distance. Specifically, SPRINTER empirically computes the distribution of the distance between pairs of cells with similar ploidies (measured as the average inferred copy number) and uses a mixture model to identify the distribution of the pairwise distances for cells within the same clone. This is done under the expectation that distances computed between cells in different clones are higher than those computed between cells belonging to the same clone. As such, the inferred component with the lowest mean is used to estimate the maximum cell-to-clone distance.

In detail, in order to automatically estimate the maximum cell-to-clone distance ϵ per sample of sequenced cells, SPRINTER empirically computes the distribution of the pairwise distances between all cells by using a randomly sampled subset of G0/1/2 phase cells sampled from the largest group of tumour cells with the same ploidy (identified using the same method described in Supplementary Note 17). We expect this mixed distribution to be composed of two groups of unknown components. The first group is formed by a single component, which is the distribution of the distances between pairs of cells that belong to the same clone. In fact, when comparing pairs of cells belonging to the same clone, the distances between cells is only influenced by the expected error rate and cell-specific CNAs, forming a distribution of pairwise cell distances within the same clone. The second group is composed of other distributions with higher values of distances that are obtained when comparing cells belonging to different clones. As such, SPRINTER deconvolves these distributions using a Gaussian mixture model and it selects the distribution of distances for cells within the same clone as the component identified with the lowest mean. Finally, using this latter distribution, SPRINTER obtains ϵ as a fixed quantile (0.7 by default in order to be conservative in the definition of clones) of the inferred distribution with the lowest mean.

17 Correction of ploidy and clone errors

SPRINTER introduces a hypothesis-testing approach to identify and correct artefactual clones derived from errors in the inferred ploidy of certain cells (where cell ploidy is measured as the average inferred copy number). In fact, as shown in previous studies[3,5], errors can frequently occur in the inference of the scale factor γ despite the use of model selection, leading to the erroneous inference of ploidy and related CNAs for certain cells and, consequently, to the identification of artefactual clones. Since artefactual clones derive from cells that should belong to other clones but have been inferred with different, erroneous ploidy and copy numbers, the key idea of the approach introduced by SPRINTER is to test whether the cells in clones with different ploidy, i.e., with a ploidy different to most other tumour cells, can be equally explained by the ploidy and CNAs of other clones. Specifically, each clone with different ploidy is compared to any other clone by inferring CNAs using the same value of γ and testing if the CNAs inferred for the cells in the former clone are significantly different to the CNAs of cells from the latter clone. As such, clones that do not pass the test are the result of ploidy errors and are combined.

In detail, SPRINTER introduces a statistical test in which every clone i inferred with a ploidy different than most other tumour cells is compared to any other tumour clone j. In particular, clones are defined to have different ploidy when the ratio between the two ploidies is higher than a fixed threshold ξ (by default equal to 1.2), i.e.,

$$\frac{\max\{{}^{1}/_{m}\sum_{m} c_{b,i}, {}^{1}/_{m}\sum_{m} c_{b,j}\}}{\min\{{}^{1}/_{m}\sum_{m} c_{b,i}, {}^{1}/_{m}\sum_{m} c_{b,j}\}} > \xi$$

As such, the test between clones i and j inferred with different ploidies is performed in two steps.

In the first step, the baseline copy numbers of all cells in clones i and j are recalculated using the same value of the scaling factor γ (i.e., assuming the clones have the same ploidy), which is obtained from the clone with the highest ploidy. This is done by scaling by γ the average RDR within every segment of each cell in clones i and j, and by rounding it to the nearest integer. Moreover, the segments are obtained per cell by combining neighbouring bins within the same chromosome that have been previously inferred as having the same copy numbers by SPRINTER.

In the second step, we test whether the cells in clones i and j have significantly different copy numbers based on the values recalculated in the first step. To do this, we assess if the cells in clone i have significantly higher pairwise distances (computed like in Supplementary Note 16 with the Hamming distance between copy numbers) to cells in clone j than another random group of cells in the same clone j. Specifically, we randomly sample groups of cells of equal size without replacement (up to 100 cells per clone) and we compute the pairwise distances between cells in clone i and the cells in the first group of clone j, as well as between cells in the two groups obtained from clone j. As such, we obtain two empirical distributions of pairwise distances (one between cells in clones i and j, and one between the two groups of cells in clone j) and use a one-sided Mood's median test to assess if the distances between cells in clones i and j are significantly higher than those between the two groups of cells in clone j.

Finally, if clones i and j are found to have significantly different distances (with a significance level of 1% with Bonferroni correction), this result indicates that the two clones are not only distinguished by errors in the identified ploidy but also by different CNAs; thus, the clones are not changed. Otherwise, if clones i and j have non-significantly different distances, clone i is disregarded and the related cells will be assigned to other existing clones. Specifically, the cells in clone i are assigned to the best other existing clone using the same Bayesian probabilistic approach applied for the clone assignment of S phase cells (Supplementary Note 18).

18 Clone-assignment of S phase cells

To enable the accurate assignment of S phase cells to clones, SPRINTER introduces a method that first corrects RDR fluctuations due to replication in each S phase cell independently, and next applies a Bayesian probabilistic approach to assign each corrected S phase cell to the maximum-a-posteriori clone.

First, SPRINTER uses the previously-inferred copy-number segments to correct the RDRs of each S phase cell independently for replication fluctuations. Since the RDR x_b of each bin b is directly proportional to the baseline copy number c_b and the replication state t_b (i.e., $x_b \propto t_b \cdot c_b$, Supplementary Note 14), SPRINTER computes a replication-corrected RDR $\dot{x}_b$ by subtracting the effect of t_b from x_b. Even though t_b is unknown, SPRINTER's segmentation has identified in the second step (Supplementary Note 14) groups of bins Γ_c with the same value c of copy numbers and, among these, it also identifies subgroups of bins $\mathrm{T}_{c,t}$ with the same value t of replication state. As such, within each segment we correct x_b of every bin b by a scaling factor that depends on both the average RDRs of Γ_{c_b} and T_{c_b,t_b} so that every bin within the same segment has the same expected value for $\dot{x}_b$. In particular, SPRINTER computes $\dot{x}_b$ as follows

$$\dot{x}_b = x_b \cdot \frac{\frac{1}{|\Gamma_{c_b}|} \sum_{b \in \Gamma_{c_b}} x_b}{\frac{1}{|\mathrm{T}_{c_b,t_b}|} \sum_{b \in \mathrm{T}_{c_b,t_b}} x_b}$$

where Γ_{c_b} is defined by all segments identified with the same copy-number state c_b, and T_{c_b,t_b} is defined by bins within these segments that are also inferred to have the same underlying value of replication state t_b by the HMM algorithm used by SPRINTER's segmentation within either the early or late replication timing group. The resulting values of the replication-corrected RDRs are thus expected to only depend on the baseline copy numbers, i.e., $\dot{x}_b \propto c_b$, and can be used for CNA analysis (Extended Data Fig. 4 and Supplementary Fig. 7).

Next, SPRINTER introduces a Bayesian approach to assign each cell to a maximum-a-posteriori clone based on the replication-corrected RDRs. If k is the number of inferred clones, we model the assignment of a cell to a clone as the discrete variable $\tau \in \{1, \dots, k\}$ such that $\tau = i$ indicates that the cell is assigned to clone i. SPRINTER thus infers the assignment τ^* that maximises the posterior probability $P(\tau \mid \dot{x}_1, \dots, \dot{x}_m)$, which is proportional to the product of the likelihood $\mathcal{L}(\tau;\, \dot{x}_1, \dots, \dot{x}_m)$ and the prior probability $P(\tau)$, i.e.,

$$\tau^* = \operatorname*{argmax}_{\tau \in \{1,\dots,k\}} P(\tau \mid \dot{x}_1, \dots, \dot{x}_m) = \operatorname*{argmax}_{\tau \in \{1,\dots,k\}} \mathcal{L}(\tau;\ \dot{x}_1, \dots, \dot{x}_m) \cdot P(\tau).$$

For every clone i, we compute $\mathcal{L}(\tau;\ \dot{x}_1, \dots, \dot{x}_m)$ by modelling each $\dot{x}_b$ as having been drawn from a Normal distribution where the mean is the expected value of the replication-corrected RDRs computed based on the clone copy numbers. Specifically, the clone copy number $C_{b,i}$ of each bin b is defined as the most frequent value for the inferred copy number c_b across all the G0/1/2 phase cells belonging to clone i. As such, the likelihood is computed for each clone i as follows

$$\mathcal{L}(\tau;\ \dot{x}_1, \dots, \dot{x}_m) = \prod_{b \in \{1,\dots,m\}} f\left(\dot{x}_b \mid C_{b,i} \frac{\sum_b \dot{x}_b}{\sum_b C_{b,i}}, \sigma^2_{i,b}\right)$$

where f is the Normal probability density function with mean $C_{b,i} \frac{\sum_b \dot{x}_b}{\sum_b C_{b,i}}$ (which is the expected value of $\dot{x}_b$ given the copy numbers of clone i) and $\sigma^2_{i,b}$ is the maximum likelihood estimate of the variance, computed using all the bins expected to have the same copy number $C_{b,i}$ in the clone. Moreover, we compute the prior probability $P(\tau)$ using the size of each clone, which we estimate using the number N_i of G0/1/2 phase cells belonging to i, i.e., $P(\tau) = {N_\tau}/{\sum_i N_i}$. SPRINTER thus assigns each cell to clone τ^*.

19 Inference of copy numbers in S phase cells

SPRINTER infers the CNAs of the identified S phase cells using the assigned clones (Supplementary Note 18). For each S phase cell, SPRINTER infers the baseline copy number c_b of every bin b by using the replication corrected RDRs $\dot{x}_b$ (calculated in Supplementary Note 18, Extended Data Fig. 4 and Supplementary Fig. 7) and by using the inferred CNAs for the clone τ^* to which the S phase cell has been assigned. In particular, SPRINTER uses the same HMM applied to infer CNAs in G0/1/2 phase cells (Supplementary Note 15), but uses the replication corrected RDRs $\dot{x}_b$ and fixes the scale factor γ based on the expected copy numbers $C_{1,\tau^*}, \dots, C_{m,\tau^*}$ of the assigned clone τ^* (defined by consensus using the corresponding G0/1/2 phase cells), i.e.,

$$\gamma = \frac{\sum_b \dot{x}_b}{\sum_b C_{b,i}}$$

The inference of the baseline copy numbers for every cell in each clone allows SPRINTER to compute a CNA-based distance between each cell and the corresponding clone, using the Hamming distance between the corresponding copy numbers across bins (like in Supplementary Note 16). SPRINTER uses this distance to identify and potentially discard outlying cells. In fact, outliers are expected in single-cell sequencing experiments due to experimental failures or other types of artefacts[1,5]. Given a fixed and user-tuneable expected proportion $\epsilon \in [0, 1]$ of outliers (by default chosen to be $\epsilon = 0.7$ based on previous studies[1,3,5]), SPRINTER excludes the ϵ-proportion of cells with the largest clone distance in the group of G0/1/2 and S phase cells independently. Note that outliers are excluded independently across G0/1/2 and S phase cells and using a fixed threshold

to allow comparison across different clones and samples without affecting the overall estimated fractions of S phase cells.

Lastly, small, rare CNAs that exclusively occur in genomic regions with only early or late replication timing cannot be identified with this method in S phase cells, since the fluctuations induced by these rare CNAs would be erroneously corrected as replication-induced fluctuations and related segments would have been excluded in the second step of SPRINTER. Therefore, SPRINTER adds a specific correction for these cases that corrects the CNAs inferred for each S phase cell whenever a replication-timing-exclusive CNA is inferred for the assigned clone (i.e., in the G0/1/2 phase cells belonging to the assigned clone, since all CNAs can be accurately inferred in the G0/1/2 phase cells given all bins and all RDR fluctuations are used in the CNA analysis of these cells). The same approach is also adopted to correct small CNAs that could be more difficult to identify in S phase cells. As such, SPRINTER identifies clone-specific copy numbers using a consensus approach (similar to previous approaches[5]), such that the clone-specific copy number of every genomic bin is defined as the most common copy number across all of the corresponding G0/1/2 phase cells. SPRINTER identifies every segment defined by these clone-specific copy numbers that only occurs in early or late genomic regions, or that is small (<5Mb by default), and corrects the corresponding copy numbers in every S phase cell assigned to the same clone to the clone-specific copy number. Using a spike-in experiment of CNAs of varying size, we showed that this approach enables SPRINTER to accurately recover most CNAs of >3Mb in size in both G0/1/2 and S phase cells (Supplementary Fig. 21).

20 Identification of G2 cells per clone

SPRINTER identifies G2 phase cells in each inferred clone separately among the corresponding G0/1/2 phase cells. Although G2 phase cells cannot be distinguished from G0/1 phase cells solely based on RDRs (Supplementary Note 15), G2 phase cells are expected to yield higher total read counts than G1 phase cells in most scDNA-seq experiments. In fact, the total read count $R_j = \sum_b r_{b,j}$ of any cell j is expected to scale with the DNA content of the cell, especially for tagmentation-based technologies such as DLP+[1] (Supplementary Fig. 8). Since G2 phase cells have doubled their DNA content compared to G0/1 phase cells within the same clone, we expect that $R_j > R_l$ for a G2 phase cell j and a G0/1 phase cell l. Based on this, SPRINTER introduces an importance sampling method to estimate the fraction μ of G2 phase cells in each clone by deconvolving the distributions of total read counts generated by either G0/1 or G2 phase cells using a Negative Binomial mixture model. Additionally, the method integrates information from the identified S phase cells: since G2 phase cells are also expected to yield higher read counts than S phase cells on average (Supplementary Fig. 8), we constrain the inference of μ such that the resulting G2 phase cells have an expected read count higher than the expected read count of S phase cells. As such, the probability of each cell being in G0/1 or G2 phase is computed using the likelihoods of the fitted model and a uniform prior, and G2 phase cells are defined as those with a probability below a certain threshold of being in G0/1 phase (<0.3 by default). Below, we describe the details of this method.

Among all the G0/1/2 phase cells identified in each clone, SPRINTER identifies G2 phase cells per clone by using an importance sampling method based on the total read count $R_j = \sum_b r_{b,j}$ of every G0/1/2 phase cell j in the clone. In particular, we model the total read counts of cells within the same phase as a Negative Binomial distribution, such that $R_i \sim NB(\mathcal{R}^1, \Delta^1)$ for total read counts of G0/G1 phase cells and $R_j \sim NB(\mathcal{R}^2, \Delta^2)$ for total read counts of G2 phase cells. Note that a Negative Binomial model was chosen to account for potential overdispersion in the observed total read counts in addition to standard Poisson models. Moreover, since the total read counts of different cells can also be affected by varying ploidy and cell-unique CNAs, we use a quantile sigmoid regression to correct the total read counts of all cells based on the average copy number estimated by SPRINTER. Note that a sigmoid regression has been chosen since the relationship is not necessarily linear in scDNA-seq as shown in the generated datasets (Supplementary Figs. 8 and 10).

Since we do not know which cells are in either G0/G1 or G2 phase, we model the observed total read counts $R_1, \ldots, R_n$ for the n sequenced cells as a mixture of the two unknown distributions $NB(\mathcal{R}^1, \Delta^1)$ and $NB(\mathcal{R}^2, \Delta^2)$. As such, we develop an importance sampling approach to obtain a maximum-a-posteriori estimate of the fraction μ of G2 cells in the clone, used to fit a model to separately classify G0/G1 vs G2 phase cells. Specifically, plausible values of μ are chosen from a candidate distribution that we define as the weighted combination of two uniform distributions in which a higher weight (2/3 by default) is assigned to values of μ that are proportionally lower than or equal to the fraction of inferred S phase cells. Note that these weights are chosen since the number of G2 cells is generally expected to be lower than the number of S phase cells in a clone (since S phase tends to be longer than G2 phase), but our method does not disregard the opposite alternative. Moreover, the maximum value μ is estimated based on the fact that G2 phase cells are expected to have higher total read counts than S phase cells on average. Therefore, the maximum value of μ is estimated as the fraction of G2 phase cells with total read counts higher than the mean total read count in S phase cells on average across clones. For each sampled value μ among the candidates, we fit $NB(\mathcal{R}^1, \Delta^1)$ and $NB(\mathcal{R}^2, \Delta^2)$ by separating the $n \cdot \mu$ cells with lowest and highest total read counts. We then use the standard Broyden-Fletcher-Goldfarb-Shannon algorithm in each group to find the maximum-likelihood estimates of the corresponding parameters. The likelihood of the fit is used as the importance weight for each sampled value of μ and the posterior probability is estimated by using a reverse-linear transformation on these weights. Based on the models fitted using a maximum-a-posteriori value of μ, SPRINTER calculates the posterior probability of every cell being in either G1 or G2 phase using a uniform prior. Based on a fixed threshold to control the sensitivity of inferring G2 phase cells (30% by default), SPRINTER infers as G2 phase all the cells that have a probability below this threshold of belonging to the distribution of G1 phase cells. All the remaining cells are defined as G0/1 phase cells.

21 Single-cell whole-genome DNA sequencing

We performed single-cell whole-genome DNA sequencing on all cells from the HCT116 ground truth datasets and from the NSCLC case of the TRACERx/PEACE patient using the DLP+ protocol as previously described[1,3]. Given that only snap-frozen patient tissue was available for this study, all

HCT116 cells and patient tissue samples underwent single nuclei isolation prior to DLP+ sequencing. Overall, the protocol is composed of three main steps, briefly described below.

Single nuclei preparation and staining

Single nuclei were isolated from snap-frozen patient tissue using a modified version of the previously described "Frankenstein protocol" (dx.doi.org/10.17504/protocols.io.bqxymxpw). In brief, patient tissue between 1x1mm and 3x3mm in size was transferred to a 15ml falcon tube along with 500µl of chilled Nuclei EZ Lysis Buffer (Sigma, N3408) and briefly homogenised using the TissueRuptor II (Qiagen, 9002757). The homogenate was topped up with 1ml of chilled Nuclei EZ Lysis Buffer and incubated on ice for 5 minutes, with gentle mixing 1-2 times during the incubation. The nuclei suspensions were filtered using a 70µm mesh filter into a fresh 2ml tube and pelleted at 500g for 5 minutes at 4°C. The supernatant was removed, and the nuclei were gently resuspended in 1.5ml of Nuclei EZ Lysis Buffer before incubating for 5 minutes on ice. After incubation, the nuclei were pelleted, the supernatant was removed, and the nuclei were resuspended in 1ml of a 1:1 ratio of 1x PBS + 0.04% BSA and Nuclei EZ Lysis Buffer. The nuclei were pelleted again and resuspended in 1ml of 1x PBS + 0.04% BSA before filtering through a 40µm mesh filter and quantified using the LUNA-FX7 automated cell counter (Logos Biosystems).

The nuclei suspensions were stained as previously described[1]. In brief, nuclei were stained with LIVE/DEAD Fixable Far Red (ThermoFisher, L34973), and incubated at 37°C for 20 minutes before being pelleted and resuspended in 500µl of 1x PBS + 0.04% BSA. For the primary tumour samples, the nuclei were stained with Hoechst 33342 (Thermofisher Scientific, 62249) and incubated on ice for 20 minutes. The nuclear suspensions were then quantified using the LUNA-FX7 and diluted to an optimal loading concentration of 200 nuclei/µl in preparation for isolation using the CellenONE F1.4 instrument (Cellenion).

For the ground truth dataset, nuclei were isolated from HCT116 single cell suspensions as previously described[1]. In brief, the volume of cells was doubled with Nuclei EZ Lysis Buffer before being pelleted at 500g for 5 minutes at 4°C. The supernatant was removed, and the nuclei were resuspended in a suitable volume of 1x PBS + 0.04% BSA before being quantified with the LUNA-FX7 and diluted to 200 nuclei/µl in preparation for isolation using the CellenONE F1.4. Staining was not necessary as the nuclei previously underwent Hoechst 33342 staining during the FACS process.

CellenONE nuclei isolation and nuclear imaging

Single nuclei suspensions were loaded into the CellenONE F1.4, a contactless piezoelectric dispensing instrument, and dispensed using a glass nozzle into nanochips (TakaraBio, 340046) containing 5184 individual nanowells pre-printed with Illumina-compatible combinatorial dual-indexed sequencing primers (Integrated DNA Technologies) as previously described[1]. As part of the CellenONE's nuclear dispensing process, morphological information, including nuclear diameter, for each isolated nucleus was automatically captured using the CellenONE's software (version 2.0.1.1029). These nozzle images were used to measure nuclear diameter in this study. Note that due to the curvature of the dispensing nozzle, nuclear diameter recorded by the nozzle-based images can appear consistently slightly larger when compared to microscopy imaging. For the

HCT116 ground truth datasets, all diploid and tetraploid cells were dispensed into the same nanochip for processing. Similarly, for the NSCLC case, all cells obtained from the same tumour sample were dispensed into the same nanochip, so that library preparation was performed simultaneously on all cells within the same nanochip and subsequently sequenced together. Prior to library preparation, all nanochips underwent fluorescent microscopy imaging to verify single-cell occupancy. Specifically, this imaging was performed using a Zeiss Axio Observer Z1 widefield microscope, equipped with a SpectraX lightsource using a Zeiss Plan Neofluar 10X/0.3NA lens. Image acquisition was done using Micro-Manager and using the Micro-Manager High Content Screening (HCS) Site Generator plugin.

DLP+ library preparation and sequencing

Libraries were prepared using the DLP+ protocol as previously described[1,3]. In brief, nuclei were enzymatically and heat lysed (Viagen DirectPCR Lysis Reagent, 301-C), followed by the addition of a tagmentation mix comprising 10nL TDE1 buffer, 10nL BLT enzyme, 20nL PCR water (Illumina DNA Prep, 20060060),) and then neutralisation (Qiagen Protease, 19157). Libraries were PCR amplified for a total of 11 cycles using Enhanced PCR Mix (Illumina DNA Prep), pooled by centrifugation, and size selected using SPRIselect purification beads (Beckman Coulter, B23319). Library quantification was performed using the Qubit Fluorometer (ThermoFisher, Q33231) and library size measured using the Tapestation 4200 (Agilent, 5067-5583) before sequencing with 150bp paired-end reads on the Illumina NovaSeq 6000 at the Genomics Science Technology Platform at the Francis Crick Institute.

22 Generation of a ground truth dataset of cell cycle-sorted cells

We generated ground truth sequencing datasets with diploid and tetraploid cells in known cell cycle phases sequenced using the DLP+ protocol. To avoid cross-contamination between cell cycle phases, a common occurrence when using standard FACS techniques used in previously described[2], we used an improved flow cell sorting approach based on previous studies[26] which uses two independent signals to sort the cells of interest. The first is EdU, which is incorporated into actively replicating DNA and has been shown to accurately and comprehensively capture S phase cells[26], and the second is DNA Hoechst 33342 dye to measure DNA content (Supplementary Fig. 9). To apply this approach, we chose the colorectal cell line HCT116 as it provided an isogenic system which had already been analysed in previous longitudinal studies[7], and enabled the creation of both diploid and tetraploid ground truth datasets[7]. In particular, the availability of both diploid and tetraploid ground truth datasets allows the validation of SPRINTER at different ploidies, which is important since whole genome duplications (WGDs) are frequent in cancer and may affect single-cell analyses due to increased CNA rates[25,27]. The diploid and tetraploid HCT116 cells were provided by the authors of the related longitudinal study[7] from the Francis Crick Institute (London, UK).

We first labelled the HCT116 cells with Click-iT EdU and fixed and stained using the Click-iT Plus EdU Flow Cytometry Assay Kit (C10634 Invitrogen), halting further progression through the cell cycle. Cells were stained with 2μg/ml Hoechst 33342 then flow sorted on a BD Influx cell sorter

(BD, San Jose, CA, USA) using a 140 micron nozzle, with pressure maintained at 14 psi. Data was analysed using BD FACS Software v1.2.0.142 (BD, San Jose, CA, USA). Cells were simultaneously and electrostatically sorted into 5 uniform fractions of different cell cycle phases (G1, early S, mid S, late S, and G2) based on both EdU (Alexa Fluor 647, excited with a 642nm laser and emission collected in a 670/30BP filter) and DNA Hoechst 33342 dye (excited using a 405nm laser and emission collected in a 460/50BP filter), with both parameters displayed on a linear scale (Supplementary Fig. 9). Compared to previous studies[1,2], our approach is based on the use of discrete gates rather than continuous sorting. While this discrete approach does not sample cells as comprehensively as previous approaches, it provides improved precision in the classification of the different phases. Finally, the sorted cells were single-cell whole-genome DNA sequenced using the DLP+ protocol as described above (Supplementary Note 21), thus generating ground truth datasets with diploid and tetraploid cells.

23 Processing single-cell whole-genome DNA sequencing data

The generated scDNA-seq data was processed and aligned to the human reference genome after standard quality control, obtaining a single-cell pseudo-bulk BAM file for each sample, for all cells that were sequenced together. Initial quality control of raw paired-end reads was performed using FastQC (v0.11.8) and FastQ Screen (v0.13.0, flags: --subset 100000; --aligner bowtie2) along with MultiQC (v1.10.1) to generate related reports. fastp (v0.20.0) was used to remove adapter sequences before aligning the trimmed reads to the hg19 genome assembly (including unknown contigs) using BWA-MEM (v0.7.17). Alignment was performed individually for each single cell and during this process we generated a unique barcode for each cell which was incorporated into the read group information in the resulting BAM file. The single-cell BAM files were then deduplicated using sambamba markdup (v0.7.0, flags: --remove-duplicates). Following alignment and deduplication, all the single-cell BAM files were merged together into a single pseudo-bulk BAM file for each sample using sambamba merge (v0.7.0). During this merge process, read group information was retained allowing the reassignment of all sequencing reads back to the initial cell. Further quality control following alignment was performed using a combination of Samtools (v1.9) and Picard (v2.25.4) with MultiQC (v1.10.1), generating related reports.

24 Bioinformatics analysis of scDNA-seq data

SPRINTER was applied independently to each generated pseudo-bulk BAM file for the ground truth datasets and the NSCLC samples using default parameters. Specifically, read counts were calculated and control sequencing reads were simulated using the related command available as part of the CHISEL algorithm (v1.1.4)[5]. Moreover, SPRINTER was applied to the previous TNBC and HGSC datasets using the available read counts provided in previous studies[3]. In particular, in these datasets SPRINTER was applied independently to each collection of single-cell sequencing reads generated together from the same DNA sequencing library. Since different collections might have been generated for cells belonging to the same clone, clones identified in each library have been clustered

into the same clone based on the similarity of inferred CNAs (using hierarchical clustering with maximum difference 5%) when comparing S fractions. In downstream analyses involving multiple clones in the same tumour, only tumours with more than one tumour clone identified have been considered. In all cases, the default set of input replication scores have been used as input to SPRINTER (Supplementary Note 10) and only cells with >100k sequencing reads have been selected for SPRINTER's analysis based on recommendations from previous scDNA-seq studies[5]. For each clone identified by SPRINTER, the distributions of the fractions of S and G2 phase cells were computed by bootstrapping all the cells in the same sequenced sample.

On the ground truth datasets, the previous methods for inferring S phase cells, CCC and MAPD, were applied using the available implementations: for CCC the available single-cell pipeline distributed through Bioconda[28] was used (v0.6.46), which also included the related HMMcopy algorithm[1] for CNA analysis, and for MAPD the implementation reported in the previous related study[2] was applied. Moreover, a new version of MAPD, called replication-timing MAPD (rtMAPD), has been executed, extending the implementation of MAPD to calculate the median absolute deviation of pairwise differences (i.e., the summary statistic used by MAPD) only between neighbouring genomic windows with different replicating timing (i.e., early vs late). Both previous methods were executed with default parameters using the same input read counts used for SPRINTER and using the median bin size calculated by SPRINTER across all cells (~250kb). For each previous method, all the possible thresholds for the classification scores calculated by each method have been tested as part of the ROC analysis to assess the performance of separating G1 from S and G2 phase cells. As done in previous studies[3,6], the clones used for comparing previous approaches were identified by clustering the G0/1/2 phase cells identified by either CCC or MAPD using hierarchical clustering with the standard Ward method applied on related RDRs and using the same number of clones inferred by SPRINTER on the same cells. Following the procedure proposed in previous studies[6], each S phase cell was thus assigned to the clone with maximum Pearson correlation considering the clones inferred based on the results of either CCC or MAPD.

25 Identification of SNVs and driver mutations in single-cell data

Somatic single-nucleotide variants (SNVs) were identified from the NSCLC dataset using the variant calling workflow of Mutect2 (GATK, v4.2.0), applied to each generated single-cell pseudo-bulk BAM file independently. Specifically, the pseudo-bulk BAM files were used as input for Mutect2 in tumour-only mode. During the calling process, f1r2 statistics were extracted and passed into 'LearnReadOrientationModel' and 'GetPileupSummaries' was used to generate input for 'CalculateContamination'. Finally, 'FilterMutectCalls', including the orientation bias priors from 'LearnReadOrientationModel', the contamination table from 'CalculateContamination', and the call statistics from Mutect2 were used to filter the set of called variants with high confidence.

Germline variants that were missed by Mutect2 were further identified and removed using all the normal diploid clones identified by SPRINTER, as done in previous studies[5]. In particular, normal diploid clones were identified using the CNAs inferred by SPRINTER as those with >98% of the

genome with inferred baseline copy numbers equal to 2. As such, any variant with at least one supporting read found in the normal diploid clones was discarded from the called somatic mutations.

Among the SNVs called in the NSCLC dataset, putative driver mutations were identified using a collection of existing tools. Mutations were mapped to canonical transcripts and annotated using the Ensembl Variant Effect Predictor (VEP, v109)[29] combined with the plugins CADD[30] (v16), LOFTEE[31], and SpliceAI[32]. Additionally, the CHASMplus, CHASMplus LUAD, and CHASMplus LUSC modules[33] from openCRAVAT[34] (v2.3.0) were used to retrieve CHASMplus scores and the Cancer Genome Interpreter pipeline[35] was used to retrieve boostDM scores[36]. Driver classifications based on oncoKB[37] were also inferred. The resulting outputs were matched by genomic position, reference bases, and mutated bases. Tier 1 or tier 2 genes from The COSMIC Cancer Gene Census[38] (v98) were used in the classification of driver mutations, such that an SNV was classified as a driver mutation if the SNV passed at least one of the following criteria:

- any SNV with a CADD PHRED score above 30 in a COSMIC Cancer Gene;
- any high-confidence LOFTEE call or any SNV with a SpliceAI score > 0.8 in a gene classified as a tumour-suppressor gene in the COSMIC Cancer Gene Census;
- any SNV classified as driver by boostDM;
- any driver SNV with a p-value <0.001 either in CHASMplus, CHASMplus LUAD or CHASMplus LUSC.

In the analysis of the TNBC and HGSC datasets, likely driver mutations or mutations with functional impact that are associated with high clone proliferation were chosen with the following criteria:

- any SNV classified as a driver by boostDM or oncodriveMUT in the Cancer Genome Interpreter pipeline;
- any SNV classified as a driver by oncoKB;
- any LOFTEE call (for loss of function);
- any SNV with a SpliceAI score > 0.2.

26 Phylogenetic analysis of SNVs and CNAs

We reconstructed the tumour phylogeny for the clones inferred by SPRINTER in the NSCLC dataset using both SNVs and CNAs. In particular, SNVs and driver mutations were identified using a pseudo-bulk approach[1,3,5] and standard existing methods (Supplementary Note 25). While existing methods can reconstruct tumour phylogenies from single-cell SNVs[39], these methods cannot be directly applied to SPRINTER's clones due to the presence of subclonal SNVs, i.e., SNVs that are only present in a subset of the cells within the same clone. Moreover, while methods to reconstruct tumour phylogenies from clone-specific CNAs[40] also exist, these methods do not integrate both SNVs and

CNAs in the reconstruction of tumour phylogenies. Therefore, we devised a three-step approach to overcome these challenges by integrating and extending existing methods.

First, we identified clonal SNVs in each clone, i.e., SNVs that are present across all cells in the clone and thus have a cellular frequency of 1 in the clone. As shown in previous studies[41], inferring the cellular frequency of SNVs from DNA sequencing reads is challenging, for instance due to the impact of CNAs that vary SNV multiplicities (i.e., the number of copies of the locus harbouring the SNV). Furthermore, other factors, such as sequencing errors, SNVs present at low frequencies, and low sequencing coverage within a clone also complicate this inference. Therefore, we developed a statistical approach based on previous Binomial models[41] to classify SNVs into clonal, absent, or unknown categories by testing two hypotheses for each SNV in each clone. First, we tested the hypothesis that the SNV is present in all cells within the clone. Second, we tested the hypothesis that the SNV is absent from the clone and any observed variant reads are due to errors. We genotyped an SNV as clonal if we could not reject the first hypothesis and could reject the second hypothesis, absent if we could reject the first hypothesis and could not reject the second hypothesis, and unknown otherwise. Further details are in Supplementary Note 27.

Second, we reconstructed the topology of the tumour phylogeny by applying the HUNTRESS algorithm[39] (v0.1.2) to the clonal SNVs identified in the first step. Specifically, HUNTRESS was chosen due to its scalability to hundreds of SNVs and its ability to deal with missing data (i.e., the mutations classified as unknown in certain clones). However, HUNTRESS cannot account for mutation losses, i.e., SNVs that are lost from the cells of a clone due to CNAs[41]. To overcome this limitation, we reconstructed the topology by only selecting SNVs that are present in chromosomes not affected by copy-number deletions, according to previous classifications of copy-number events[5]. Specifically, we selected SNVs from chromosomes 1, 3, 4, and 17. Moreover, since HUNTRESS has only been tested in cases with hundreds of SNVs in previous studies[39], we applied it on 1000 unique subsampled SNVs.

Lastly, we inferred the SNVs and CNAs of the ancestral clones present in the reconstructed topology. For each ancestral seeding clone, we inferred the state of the SNVs in chromosomes 1, 3, 4, and 17 that were not used by HUNTRESS in the previous step. To do this, in every ancestral seeding clone we inferred the state of each SNV independently by comparing the classification of the SNV between the descendant and non-descendant clones. Specifically, the SNV was defined as clonal in an ancestral seeding clone if it was clonal in all descendant SPRINTER clones, or if it was clonal in at least one descendant and one non-descendant SPRINTER clone (details in Supplementary Note 28). Note that SNVs might not be clonal in some descendant clones due to their unknown status (e.g., due to low sequencing coverage) or due to the occurrence of mutation losses resulting from CNAs. Otherwise, the SNV is classified as absent in the ancestral clone. Finally, the CNAs of all ancestral clones were reconstructed using the MEDICC2 algorithm[40] (v1.0.2) by fixing the reconstructed topology obtained with SNVs and using the inferred CNAs for SPRINTER clones, which correspond to the leaves of the phylogeny.

27 Genotyping SNVs in single-cell clones

We genotyped the state of each SNV as either clonal, absent, or unknown in every clone inferred by SPRINTER using a pseudo-bulk approach as in previous studies[1,3,5]. For each SNV, we pooled the sequencing reads from all cells in each clone inferred by SPRINTER and, for each clone, observed the total number of reads $\tau \in \mathbb{N}$ and the number of variant reads $v \in \{0, \dots, \tau\}$. As such, we introduced a statistical hypothesis-testing approach to genotype each SNV in each clone. To do this, we devised a generative model for v that depends on whether the SNV is present at the locus of a clone, or not. When the SNV is present, we model v as a draw from a Binomial distribution that depends on the observed total number of reads τ and the clone-specific true variant allele frequency (VAF) $\varphi \in [0,1]$, i.e., $v \sim \text{Binomial}(\tau, \varphi)$. The true VAF φ is not observed but, based on previous studies[41], it can be modelled as a function of the copy number c at that locus in the clone, the mutation multiplicity ω (i.e., the number of copies harbouring the mutation), and the cellular frequency $\gamma \in [0, 1]$ (also called cancer cell fraction), which represents the fraction of cancer cells in the clone that harbour the SNV. As such, the VAF φ is modelled as follows

$$\varphi = \frac{\omega \cdot \gamma}{c}$$

While c is calculated by SPRINTER and γ is only evaluated when equal to 1 in this method, ω is unknown. However, ω can be marginalised out during the probability calculation since ω can only have values between 1 and c, i.e., $P(v|c,\gamma) = \sum_{\omega=1}^{c} P(v|c,\gamma,\omega) \cdot {}^{1}/_{c}$. Lastly, when the SNV is absent, we model v as a draw from a Binomial distribution that depends on τ and a fixed estimate of the error rate $\lambda \in [0,1]$, i.e., $v \sim \text{Binomial}(\tau, \lambda)$. In this study, we set λ at 5% to account for both sequencing and clone assignment errors.

Using this model, we applied a two-step statistical approach based on hypothesis testing to infer the genotype of each SNV in each clone. First, we tested the hypothesis that the SNV is clonal in the clone, i.e., present in all cells within the clone with cellular frequency $\gamma = 1$. Second, we tested the hypothesis that the SNV is absent from the clone and any observed variant reads v are due to errors. Note that in all cases the p-values could be computed exhaustively and exactly using the binomial distributions and summing their probability values when considering varying values of v. We genotyped an SNV as clonal in a clone if we could not reject the first hypothesis (i.e., the SNV is present in all cells within the clone) and could reject the second hypothesis (i.e., the SNV is absent from the clone and observed reads are due to errors) using a conservative significance level of 10%. We genotyped an SNV as absent from a clone if we could reject the first hypothesis and could not reject the second hypothesis. Finally, we genotyped an SNV as unknown if we could neither reject the first nor second hypotheses, or if we could reject both the first and second hypotheses.

28 Reconstruction of the ancestral state of SNVs in single-cell clones

Given the topology of the tumour phylogeny reconstructed using a subset of SNVs not affected by copy-number deletions (Supplementary Note 26), we inferred the state of each remaining SNV in chromosomes 1, 3, 4, and 17 in every ancestral seeding clone. To do this, we developed an

approach to infer the state of each SNV by comparing the state of the SNV between the descendant and non-descendant extant clones inferred by SPRINTER, i.e., the extant, observed clones corresponding to the leaves of the reconstructed tumour phylogeny. In particular, we classified an SNV as clonal in an ancestral clone if the SNV was clonal in all descendant leaves, or if the SNV was clonal in at least one descendant leaf and at least one non-descendant leaf. We assigned an SNV as unknown if the genotype was unknown in all descendant leaves. Otherwise, we assigned an SNV as absent. For the special case of the most-recent common ancestor tumour clone, we compared the descending left and right branches. We assigned an SNV as clonal if the SNV was clonal in the descendant extant clones within both branches, unknown if the SNV was unknown in the descendant leaves within at least one branch, and absent otherwise.

29 Reconstruction of metastatic migrations and seeding genetic distance

Metastatic migrations were reconstructed by applying the MACHINA algorithm[42] (v1.2) to the reconstructed tumour phylogeny. Specifically, MACHINA was executed in the polytomy-resolution mode and was run considering three possible seeding patterns of increasing complexity: primary seeding only, single-source metastasis-to-metastasis seeding, and multi-source metastasis-to-metastasis seeding. As such, the most parsimonious and simplest pattern was chosen, corresponding to single-source metastasis-to-metastasis seeding. Note that re-seeding patterns have been excluded since the primary tumour was resected prior to metastatic progression in the TRACERx/PEACE case analysed in this study. As such, seeding clones have been identified as the ancestral clones that underwent metastatic migrations.

A seeding genetic distance was calculated between each SPRINTER clone and the closest seeding clone using both SNVs and CNAs. For SNVs, the seeding distance was calculated as the fraction of non-truncal SNVs (i.e., SNVs not acquired in the trunk of the reconstructed phylogeny) in chromosomes 1, 3, 4, and 17 present in only one of the clones over the total number of non-truncal SNVs present in both clones. For CNAs, the seeding distance was calculated using the Hamming distance to compare the presence of copy-number breakpoints (i.e., different copy numbers between each pair of neighbouring segments) between the two clones.

30 Identification of clone-specific altered replication timing

SPRINTER's results were leveraged to identify clone-specific altered replication timing (ART) for the tumour clones inferred in the NSCLC dataset with respect to the reference replication profiles obtained from normal cells. The identification of ART is based on previous replication timing approaches[2,18] and is included as an additional feature in the SPRINTER algorithm, composed of three steps for each SPRINTER-identified tumour clone. First, the most likely mid S phase cells are identified per clone as the top quantile of assigned cells (by default 0.3) with the highest difference in RTP values (details in Supplementary Note 14) between early and late genomic regions on average across chromosomes. Second, similar to the approach used in previous replication timing studies[2,18], the RTP values for the identified mid S phase cells are aggregated per clone, since their averages can

be used to identify early and late replicating regions. In fact, higher and lower values of RTPs indicate genomic regions that are replicating early and late, respectively (Supplementary Note 14). Moreover, RTPs are recalculated using the final and corrected CNAs inferred by SPRINTER. Lastly, the thresholds to classify early and late genomic regions are inferred by fitting two Normal distributions similar to the first step of SPRINTER (Supplementary Note 10). Since these classifications can be affected by false positives and sequencing artefacts, in this study we only considered ART to be present in genomic regions that had the same classification in most clones of more than two samples from the same tumour. As such, ART was identified in genomic regions inferred with early or late replication timing, but that were classified as late or early, respectively, from the reference replication profiles obtained from normal cells only.

31 Expression analysis to support inferred ART

To support the inferred ART classifications, two analyses were performed integrating previous matched bulk RNA-sequencing data generated for regions of the same primary tumour[43]. This is because late-to-early and early-to-late ART is known to generally be associated with increased and decreased gene expression compared to normal tissue without ART, respectively[18,44]. In the first analysis, we performed a gene set variation analysis[45] using GSEApy[46] (v.1.1.2) and showed that gene sets with late-to-early and early-to-late ART identified per sample have high and low enrichment scores of gene expression, respectively, in every available sample from patient CRUKP9145 (equivalently named CRUK0516, Supplementary Fig. 43a). To show that these results are specific to the ART inferred for this patient, we also showed that arbitrary scores are obtained when using gene expression data obtained from 915 tumour samples from 347 other TRACERx patients (Supplementary Fig. 43b).

In the second analysis, for a subset of ART specifically affecting genes known to be involved in cancer proliferation or metastatic potential, we performed a differential gene expression analysis using the same method as in previous TRACERx studies[43] based on DESeq2[47] (implemented via PyDESeq2 v.0.4.7). Specifically, we compared the gene expression measured in the samples with a related ART event to the expression measured in different sets of other samples not expected to have the same ART event. For genes with ART shared by most clones in all primary tumour and metastatic samples, we compared gene expression in all CRUKP9145 samples with all normal and all tumour samples from the other 347 TRACERx patients with RNA sequencing data available (130 normal and 915 tumour samples)[43]. For ART only present in clones from one or two metastatic clades in distinct branches of the phylogenetic tree, we additionally compared the related gene expressions in the tumour samples mostly related to the second and third clades (i.e., primary regions 3 and 5, and the left adrenal metastasis pre-mortem relapse sample) with the gene expressions measured from the other samples (i.e., primary regions 1, 2, and 7) that have been shown to be mostly related to the first metastatic clade (Fig. 3d). Finally, for ART only present in the third metastatic clade and especially in clones found in the left adrenal metastasis (i.e., ART affecting *KRAS*), we additionally compared the related gene expression in all primary tumour samples of CRUKP9145 with the expression measured in a pre-mortem relapse sample of the left adrenal metastasis. In all these

comparisons, early-to-late and late-to-early ART was found to be associated with a corresponding decrease or increase in gene expression (Fig. 4d).

32 Analysis of ctDNA

The analysis of circulating-tumour DNA (ctDNA) has been performed for the NSCLC case. Blood samples were collected and processed to plasma and ctDNA isolated in previous studies[10,48], with four longitudinal blood samples available for patient CRUKP9145 (equivalently named CRUK0516). Specifically, a personalised deep sequencing panel tracking 196 mutations was designed for CRUKP9145 using multi-region exome sequencing of surgically excised tumour tissue as previously described[48]. Across the four samples, each mutation was sequenced to a mean unique depth of 7594X (IQR = 4432-10150), only considering unique reads with at least five supporting duplicates. Tracked SNVs were matched to SPRINTER's identified single-cell clones using the phylogenetic analysis described above (Supplementary Note 26). The ECLIPSE method[48] (v1.0.0) was applied to infer ctDNA clonal composition over time using copy-number states from matched tissue exome sequencing as previously described[48], but using clone assignments from SNVs unique to SPRINTER clones and their phylogenetic ancestors. For each clone with tracked SNVs, a ctDNA shedding index at the primary tumour time point was calculated by either (i) subtracting the frequency of the SNVs (i.e., cancer cell fractions) as measured by bulk or single-cell sequencing in the primary tumour from the frequency of the same SNVs measured in ctDNA samples by ECLIPSE, or (ii) subtracting the clone proportion (i.e., proportion of cells uniquely assigned to the clone) as measured in either bulk or single-cell sequencing in the primary tumour from the measured ctDNA clone proportion (measured by subtracting the SNV frequencies of different clones according to the ancestral relationships defined by the reconstructed phylogeny, as described in previous studies[9,41,42]). To confirm consistency across all alternative ways of calculating the ctDNA shedding index, all calculated versions of the ctDNA shedding index were correlated to clone S phase fractions, measured in ancestral clones as either the maximum or median S phase fraction of all descendant clones inferred by SPRINTER.

33 Rates of clone-specific genomic variants in individual cells

In the TNBC and HGSC datasets, the single-cell rates of clone-specific SNVs, SVs, and CNAs in individual cells were calculated using the variants inferred per cell in previous studies[3]. For SNVs, the rate per cell was calculated by normalising the number of subclonal SNVs observed in the cell (i.e., observed variants for which the hypothesis of being clonal with a cellular frequency of 1 in the corresponding clone could be rejected, Supplementary Note 27) by the total number of SNVs in the cell, including all clonal SNVs found in the corresponding clone and all subclonal SNVs observed in the cell. Since the clonal status of SVs and CNAs cannot be similarly and easily assessed, the median per-clone number of variants in the tumour was considered for SVs and CNAs instead. For SVs, the rate was calculated by normalising the number of SVs observed in a cell by the median number of SVs found across the clones from the same patient. For CNAs, the rate was calculated by

normalising the number of copy-number breakpoints (i.e., pairs of neighbouring bins with different CNAs inferred by SPRINTER) per non-S phase cell (in which inferred CNAs are more reliable) by the median number of copy-number breakpoints found across the clones from the same patient. Moreover, the rates have been normalised by the average values computed in either the TNBC or HGSC datasets to make them comparable. Lastly, all clones in either the TNBC or HGSC datasets have been partitioned into two groups of high or low proliferation based on the median of the S fractions measured by SPRINTER in each dataset independently. Accordingly, every cell has been defined as belonging to a clone with either high or low proliferation.

34 Identifying genomic alterations enriched in high proliferation clones

In the TNBC and HGSC datasets, a hypothesis testing approach has been applied to identify amplifications of known oncogenes, deletions of known tumour suppressor genes (TSGs), and driver mutations enriched in high proliferation clones. Specifically, amplifications have been identified as genomic regions with an inferred copy number higher than 1.5 times the median copy number per cell, deletions as those with an inferred copy number lower than 0.5 times the median copy number per cell, and driver mutations have been identified using a similar approach to that applied to the NSCLC dataset (Supplementary Note 25). Additionally, known oncogenes and TSGs have been obtained from the COSMIC Cancer Gene Census[38] (v99). As such, for each of these identified events, a one-sided Mann–Whitney U test has been performed comparing SPRINTER's inferred S fractions for clones without the event to the S fraction for clones harbouring the event. Moreover, we have only considered events present in clones from at least two patients and the analysis has been performed for the TNBC and HGSC datasets separately. After applying a multiple-hypothesis correction using the Benjamini-Hochberg method, a *p*-value for each test is obtained and each event passing the test is classified as significant and selected as enriched in high proliferation clones. Lastly, a gene set enrichment analysis[49] has been performed for the selected amplifications that are enriched in high proliferation clones with GSEApy[46] (v.1.1.2) using the Molecular Signatures Database (MSigDB) Hallmark 2020 pathway list.

References

1. Laks, E. *et al.* Clonal Decomposition and DNA Replication States Defined by Scaled Single-Cell Genome Sequencing. *Cell* **179**, 1207-1221 e22 (2019).
2. Massey, D.J. & Koren, A. High-throughput analysis of single human cells reveals the complex nature of DNA replication timing control. *Nat Commun* **13**, 2402 (2022).
3. Funnell, T. *et al.* Single-cell genomic variation induced by mutational processes in cancer. *Nature* **612**, 106-115 (2022).
4. Minussi, D.C. *et al.* Breast tumours maintain a reservoir of subclonal diversity during expansion. *Nature* **592**, 302-308 (2021).
5. Zaccaria, S. & Raphael, B.J. Characterizing allele- and haplotype-specific copy numbers in single cells with CHISEL. *Nat Biotechnol* **39**, 207-214 (2021).
6. Andor, N. *et al.* Joint single cell DNA-seq and RNA-seq of gastric cancer cell lines reveals rules of in vitro evolution. *NAR Genom Bioinform* **2**, lqaa016 (2020).
7. Dewhurst, S.M. *et al.* Tolerance of whole-genome doubling propagates chromosomal instability and accelerates cancer genome evolution. *Cancer Discov* **4**, 175-185 (2014).
8. Al Bakir, M. *et al.* The evolution of non-small cell lung cancer metastases in TRACERx. *Nature* **616**, 534-542 (2023).
9. Frankell, A.M. *et al.* The evolution of lung cancer and impact of subclonal selection in TRACERx. *Nature* **616**, 525-533 (2023).
10. Abbosh, C. *et al.* Phylogenetic ctDNA analysis depicts early-stage lung cancer evolution. *Nature* **545**, 446-451 (2017).
11. Yushkevich, P.A. *et al.* User-guided 3D active contour segmentation of anatomical structures: significantly improved efficiency and reliability. *Neuroimage* **31**, 1116-28 (2006).
12. Ryba, T. *et al.* Evolutionarily conserved replication timing profiles predict long-range chromatin interactions and distinguish closely related cell types. *Genome Res* **20**, 761-70 (2010).
13. Yaffe, E. *et al.* Comparative analysis of DNA replication timing reveals conserved large-scale chromosomal architecture. *PLoS Genet* **6**, e1001011 (2010).
14. Consortium, E.P. An integrated encyclopedia of DNA elements in the human genome. *Nature* **489**, 57-74 (2012).
15. Du, Q. *et al.* Replication timing and epigenome remodelling are associated with the nature of chromosomal rearrangements in cancer. *Nat Commun* **10**, 416 (2019).
16. Ryba, T. *et al.* Abnormal developmental control of replication-timing domains in pediatric acute lymphoblastic leukemia. *Genome Res* **22**, 1833-44 (2012).
17. Marchal, C. *et al.* Genome-wide analysis of replication timing by next-generation sequencing with E/L Repli-seq. *Nat Protoc* **13**, 819-839 (2018).
18. Dietzen, M. *et al.* Replication timing alterations are associated with mutation acquisition during breast and lung cancer evolution. *Nature Communications* **15**, 6039 (2024).
19. Luo, Y. *et al.* New developments on the Encyclopedia of DNA Elements (ENCODE) data portal. *Nucleic Acids Res* **48**, D882-D889 (2020).
20. Huang, W., Li, L., Myers, J.R. & Marth, G.T. ART: a next-generation sequencing read simulator. *Bioinformatics* **28**, 593-4 (2012).
21. Garvin, T. *et al.* Interactive analysis and assessment of single-cell copy-number variations. *Nat Methods* **12**, 1058-60 (2015).

22. Mallory, X.F., Edrisi, M., Navin, N. & Nakhleh, L. Methods for copy number aberration detection from single-cell DNA-sequencing data. *Genome Biol* **21**, 208 (2020).
23. Koenker, R. & Hallock, K.F. Quantile Regression. *Journal of Economic Perspectives* **15**, 143–156 (2001).
24. Zack, T.I. *et al.* Pan-cancer patterns of somatic copy number alteration. *Nat Genet* **45**, 1134-40 (2013).
25. Watkins, T.B.K. *et al.* Pervasive chromosomal instability and karyotype order in tumour evolution. *Nature* **587**, 126-132 (2020).
26. Miura, H. *et al.* Mapping replication timing domains genome wide in single mammalian cells with single-cell DNA replication sequencing. *Nat Protoc* **15**, 4058-4100 (2020).
27. Lopez, S. *et al.* Interplay between whole-genome doubling and the accumulation of deleterious alterations in cancer evolution. *Nat Genet* **52**, 283-293 (2020).
28. Gruning, B. *et al.* Bioconda: sustainable and comprehensive software distribution for the life sciences. *Nat Methods* **15**, 475-476 (2018).
29. McLaren, W. *et al.* The Ensembl Variant Effect Predictor. *Genome Biol* **17**, 122 (2016).
30. Rentzsch, P., Schubach, M., Shendure, J. & Kircher, M. CADD-Splice-improving genome-wide variant effect prediction using deep learning-derived splice scores. *Genome Med* **13**, 31 (2021).
31. Karczewski, K.J. *et al.* The mutational constraint spectrum quantified from variation in 141,456 humans. *Nature* **581**, 434-443 (2020).
32. Jaganathan, K. *et al.* Predicting Splicing from Primary Sequence with Deep Learning. *Cell* **176**, 535-548 e24 (2019).
33. Tokheim, C. & Karchin, R. CHASMplus Reveals the Scope of Somatic Missense Mutations Driving Human Cancers. *Cell Syst* **9**, 9-23 e8 (2019).
34. Pagel, K.A. *et al.* Integrated Informatics Analysis of Cancer-Related Variants. *JCO Clin Cancer Inform* **4**, 310-317 (2020).
35. Tamborero, D. *et al.* Cancer Genome Interpreter annotates the biological and clinical relevance of tumor alterations. *Genome Med* **10**, 25 (2018).
36. Muinos, F., Martinez-Jimenez, F., Pich, O., Gonzalez-Perez, A. & Lopez-Bigas, N. In silico saturation mutagenesis of cancer genes. *Nature* **596**, 428-432 (2021).
37. Chakravarty, D. *et al.* OncoKB: A Precision Oncology Knowledge Base. *JCO Precis Oncol* **2017**(2017).
38. Sondka, Z. *et al.* The COSMIC Cancer Gene Census: describing genetic dysfunction across all human cancers. *Nat Rev Cancer* **18**, 696-705 (2018).
39. Kızılkale C. *et al.* Fast intratumor heterogeneity inference from single-cell sequencing data. *Nat Comput Sci* **2**, 577-583 (2022).
40. Kaufmann, T.L. *et al.* MEDICC2: whole-genome doubling aware copy-number phylogenies for cancer evolution. *Genome Biol* **23**, 241 (2022).
41. Satas, G., Zaccaria, S., El-Kebir, M. & Raphael, B.J. DeCiFering the elusive cancer cell fraction in tumor heterogeneity and evolution. *Cell Syst* **12**, 1004-1018 e10 (2021).
42. El-Kebir, M., Satas, G. & Raphael, B.J. Inferring parsimonious migration histories for metastatic cancers. *Nat Genet* **50**, 718-726 (2018).
43. Martinez-Ruiz, C. *et al.* Genomic-transcriptomic evolution in lung cancer and metastasis. *Nature* **616**, 543-552 (2023).
44. Donley, N. & Thayer, M.J. DNA replication timing, genome stability and cancer: late and/or delayed DNA replication timing is associated with increased genomic instability. *Semin Cancer Biol* **23**, 80-9 (2013).

45. Hanzelmann, S., Castelo, R. & Guinney, J. GSVA: gene set variation analysis for microarray and RNA-seq data. *BMC Bioinformatics* **14**, 7 (2013).
46. Fang, Z., Liu, X. & Peltz, G. GSEApy: a comprehensive package for performing gene set enrichment analysis in Python. *Bioinformatics* **39**(2023).
47. Love, M.I., Huber, W. & Anders, S. Moderated estimation of fold change and dispersion for RNA-seq data with DESeq2. *Genome Biol* **15**, 550 (2014).
48. Abbosh, C. *et al*. Tracking early lung cancer metastatic dissemination in TRACERx using ctDNA. *Nature* **616**, 553-562 (2023).
49. Subramanian, A. *et al*. Gene set enrichment analysis: a knowledge-based approach for interpreting genome-wide expression profiles. *Proc Natl Acad Sci U S A* **102**, 15545-50 (2005).

TRACERx Consortium

Charles Swanton[1,2,3], Mariam Jamal-Hanjani[1,3,4], Nicholas McGranahan[1,5], Simone Zaccaria[1,6], Nnennaya Kanu[1], Olivia Lucas[1,2,6,7], Sophia Ward[1,2,8], Rija Zaidi[1,6], Abigail Bunkum[1,4,6], Alexander M. Frankell[1,2], David A. Moore[1,2,9], Wing Kin Liu[1,4], Emilia L. Lim[1,2], Sonya Hessey[1,4,6], Cristina Naceur-Lombardelli[1], Andrew Rowan[2], Gary Royle[10], Antonia Toncheva[1], Ariana Huebner [1,2,5], Bushra Mussa[1], Carlos Martínez-Ruiz[1,5], Clare Puttick[1,2,5], Corentin Richard [1], Crispin T. Hiley[1,2], Despoina Karagianni [1,25], Dhruva Biswas[1,2,26], Elizabeth Keene[1], Francisco Gimeno-Valiente[1], Ieva Usaite[1], James R.M. Black[1,2], Jeanette Kittel[1,4], Kerstin Haase[1,4], Kerstin Thol [1,5], Kexin Koh[1,4], Kristiana Grigoriadis [1,2,5], Krupa Thakkar[1], Lucrezia Patruno[1,6], Maise Al Bakir [1,2], Martin D. Forster[1,3], Michalina Magala[1], Michelle M. Leung[1,2,5], Monica Sivakumar[1], Nicolai J. Birkbak[1,2,39,40,41], Paulina Prymas[1], Rachel Scott[1,4], Robert Bentham[1,5], Roberto Vendramin [1,2,45], Sadegh Saghafinia[1], Selvaraju Veeriah[1], Sergio A. Quezada[1,25], Sharon Vanloo [1], Sian Harries[1,2,8], Siow Ming Lee[1,3], Supreet Kaur Bola[1,25], Takahiro Karasaki[1,2,4,46], Thomas Patrick Jones[1,5], Chris Bailey[2], Cian Murphy[2], Claudia Lee[2], Emma Colliver[2], Gareth A. Wilson[2], Jayant K. Rane[2,17], Katey S.S. Enfield[2], Maria Zagorulya[2], Mihaela Angelova[2], Oriol Pich[2], Rachel Rosenthal[2], Dionysis Papadatos-Pastos[3], James Wilson[3], Sarah Benafif[3,33], Tanya Ahmad[3], Imran Noorani[4,22,32], Emilie Martinoni Hoogenboom [7], Fleur Monk[7], James W. Holding[7], Junaid Choudhary [7], Kunal Bhakhri[7], Maria Chiara Pisciella[7], Pat Gorman[7], Robert C.M. Stephens[7], Steve Bandula [7], Yien Ning Sophia Wong[7,47], Jerome Nicod[8], Elaine Borg[9], Mary Falzon[9], Reena Khiroya[9], Teresa Marafioti[9], Charles-Antoine Collins-Fekete[10], Akshay J. Patel [11], Alexander James Procter[12], Arjun Nair[12,18], Asia Ahmed[12], Magali N. Taylor[12], Alexandra Rice[13], Anand Devaraj[13], Andrew G. Nicholson[13,16], Chiara Proli[13], Daniel Kaniu[13], Eric Lim[13,29], Harshil Bhayani[13], Hemangi Chavan[13], Hilgardt Raubenheimer[13], Lyn Ambrose[13], Mpho Malima[13], Nadia Fernandes[13], Paulo De Sousa[13], Pratibha Shah[13], Sarah Booth[13], Silviu I. Buderi[13], Simon Jordan[13], Sofina Begum[13], Allan Hackshaw[14], Anne-Marie Hacker[14], Aoife Walker[14], Camilla Pilotti[14], Rachel Leslie[14], Sean Smith[14], Anca Grapa[15], Angela Dwornik [17], Angeliki Karamani[17], Benny Chain[17], David R. Pearce[17], Georgia Stavrou[17], Gerasimos-Theodoros Mastrokalos[17], Helen L. Lowe[17], James L. Reading[17], John A. Hartley[17], Kayalvizhi Selvaraju[17], Leah Ensell[17], Mansi Shah [17], Maria Litovchenko [17], Piotr Pawlik [17], Samuel Gamble[17], Seng Kuong Anakin Ung [17], Victoria Spanswick[17], Yin Wu[17], Carla Castignani[19,20], Peter Van Loo[19,43,44], Stephan Beck[20], Catarina Veiga[21], Clare E. Weeden[22], Erik Sahai[22], Eva Grönroos [22], George Kassiotis[22,31], Jacki Goldman [22], Mickael Escudero[22], Philip Hobson[22], Stefan Boeing[22], Tamara Denner[22], Vittorio Barbè [22], Wei-Ting Lu[22], William Hill[22], Yutaka Naito[22], Zoe Ramsden[22], David Chuter[23], Mairead MacKenzie[23], David Lawrence[24], Davide Patrini[24], Ekaterini Boleti [27], Emma Nye[28], Richard Kevin Stone[28], Francesco Fraioli[30], Zoltan Kaplar[30,48], Jack French[33], Kayleigh Gilbert[33], Karl S. Peggs[34,35], Khalid AbdulJabbar[36], Neal Navani [37,38], Ricky M. Thakrar[37,38], Sam M. Janes[37], Paul Ashford[42], Adam Atkin[49], Eustace Fontaine [49], Felice Granato[49], Juliette Novasio [49], Kendadai Rammohan [49], Leena Joseph [49], Paul Bishop [49], Philip Crosbie[49,56,95], Sara Waplington[49], Vijay Joshi[49], Aiman Alzetani[50], Alan Kirk[51], Jennifer Whiteley[51], Mathew Thomas[51], Mo Asif[51], Nikos Kostoulas[51], Rocco Bilancia[51], Amrita Bajaj[52], Apostolos Nakas[52], Azmina Sodha-Ramdeen [52], Dean A. Fennell[52,64], Mohamad Tufail[52], Molly Scotland[52], Rebecca Boyles[52], Sean Dulloo[52,64], Sridhar Rathinam[52], Andrew Kidd[53], Angela Leek [54], Jack Davies Hodgkinson[54], Nicola Totton[54], Anshuman Chaturvedi[55,56], Katherine D. Brown[55,56], Mathew Carter[55,56], Pedro Oliveira [55,56], Caroline Dive[56,60], Colin R. Lindsay[56,62], Fiona H. Blackhall[56,62], Jonathan Tugwood[56,60], Yvonne Summers [56,62], Antonio Paiva-Correia [57], Aya Osman[58], Gary Middleton[58,66], Gerald Langman[58], Helen Shackleford[58], Madava Djearaman[58], Mandeesh Sangha[58], Babu Naidu[59], Claire Wilson[61], Matthew G. Krebs[62], Craig Dick[63], John Le Quesne [63,76,77], Domenic Marrone[65], Gillian Price[67,68], Keith M. Kerr[68,82], Gurdeep Matharu[69], Jacqui A. Shaw[69], Hanyun Zhang[70], Heather Cheyne[71], Mohammed Khalil[71], Shirley Richardson [71], Tracey Cruickshank [71], Hugo J.W.L. Aerts [72,73,74], Jason F. Lester[75], Jonas Demeulemeester[78,79,80], Judith Cave[81], Kevin G. Blyth[83,84,85], Lily Robinson[86], Peter Russell[86], Madeleine Hewish[87,88], Matthew R. Huska[89], Michael J. Shackcloth[90], Miklos Diossy[91,92,93], Zoltan Szallasi[91,92,105], Patricia Georg[94], Serena Chee[94], Roberto Salgado[96,97], Roland F. Schwarz [98,99], Tom L. Kaufmann[99,103], Sarah Danson[100,101], Thomas B.K. Watkins[102], Xiaoxi Pan[104], Yinyin Yuan[104].

[1]Cancer Research UK Lung Cancer Centre of Excellence, University College London Cancer Institute, London, UK. [2]Cancer Evolution and Genome Instability Laboratory, The Francis Crick Institute, London, UK. [3]Department of Oncology, University College London Hospitals, London, UK. [4]Cancer Metastasis Laboratory, University College London Cancer Institute, London, UK. [5]Cancer Genome Evolution Research Group, University College London Cancer Institute, London, UK. [6]Computational Cancer Genomics Research Group, University College London Cancer Institute, London, UK. [7]University College London Hospitals, London, UK. [8]Genomics Science Technology Platform, The Francis Crick Institute, London, UK. [9]Department of Cellular Pathology, University College London Hospitals, London,

UK. [10]Department of Medical Physics and Biomedical Engineering, University College London, London, UK. [11]Guy's and St Thomas' NHS Foundation Trust, London, UK. [12]Department of Radiology, University College London Hospitals, London, UK. [13]Royal Brompton and Harefield Hospitals, part of Guy's and St Thomas' NHS Foundation Trust, London, UK. [14]Cancer Research UK & UCL Cancer Trials Centre, London, UK. [15]The Institute of Cancer Research, London, UK. [16]National Heart and Lung Institute, Imperial College, London, UK. [17]University College London Cancer Institute, London, UK. [18]UCL Respiratory, Department of Medicine, University College London, London, UK. [19]Cancer Genomics Laboratory, The Francis Crick Institute, London, UK. [20]Medical Genomics, University College London Cancer Institute, London, UK. [21]Centre for Medical Image Computing, Department of Medical Physics and Biomedical Engineering, London, UK. [22]The Francis Crick Institute, London, UK. [23]Independent Cancer Patient's voice, London, UK. [24]Department of Thoracic Surgery, University College London Hospital NHS Trust, London, UK. [25]Immune Regulation and Tumour Immunotherapy Group, Cancer Immunology Unit, Research Department of Haematology, University College London Cancer Institute, London, UK. [26]Bill Lyons Informatics Centre, University College London Cancer Institute, London, UK. [27]Royal Free London NHS Foundation Trust, London, UK. [28]Experimental Histopathology, The Francis Crick Institute, London, UK. [29]Academic Division of Thoracic Surgery, Imperial College London, London, UK. [30]Institute of Nuclear Medicine, University College London Hospitals, London, UK. [31]Department of Infectious Disease, Faculty of Medicine, Imperial College London, London, UK. [32]Department of Neurosurgery, National Hospital for Neurology and Neurosurgery, UK. [33]The Whittington Hospital NHS Trust, London, UK. [34]Department of Haematology, University College London Hospitals, London, UK. [35]Cancer Immunology Unit, Research Department of Haematology, University College London Cancer Institute, London, UK. [36]Case45, London, UK. [37]Lungs for Living Research Centre, UCL Respiratory, University College London, London, UK. [38]Department of Thoracic Medicine, University College London Hospitals, London, UK. [39]Department of Molecular Medicine, Aarhus University Hospital, Aarhus, Denmark. [40]Department of Clinical Medicine, Aarhus University, Aarhus, Denmark. [41]Bioinformatics Research Centre, Aarhus University, Aarhus, Denmark. [42]Institute of Structural and Molecular Biology, University College London, London, UK. [43]Department of Genetics, The University of Texas MD Anderson Cancer Center, Houston, TX, USA. [44]Department of Genomic Medicine, The University of Texas MD Anderson Cancer Center, Houston, TX, USA. [45]Tumour Immunogenomics and Immunosurveillance Laboratory, University College London Cancer Institute, London, UK. [46]Department of Thoracic Surgery, Respiratory Center, Toranomon Hospital, Tokyo, Japan. [47]National Cancer Centre Singapore, Singapore. [48]Integrated Radiology Department, North-buda St. John's Central Hospital, Budapest, Hungary. [49]Wythenshawe Hospital, Manchester University NHS Foundation Trust, Wythenshawe, UK. [50]The NIHR Southampton Biomedical Research Centre, University Hospital Southampton NHS Foundation Trust, Southampton, UK. [51]Golden Jubilee National Hospital, Clydebank, UK. [52]University Hospitals of Leicester NHS Trust, Leicester, UK. [53]Institute of Infection, Immunity & Inflammation, University of Glasgow, Glasgow, UK. [54]Manchester Cancer Research Centre Biobank, Manchester, UK. [55]The Christie NHS Foundation Trust, Manchester, UK. [56]Cancer Research UK Lung Cancer Centre of Excellence, University of Manchester, Manchester, UK. [57]Manchester University NHS Foundation Trust, Manchester, UK. [58]University Hospital Birmingham NHS Foundation Trust, Birmingham, UK. [59]Birmingham Acute Care Research Group, Institute of Inflammation and Ageing, University of Birmingham, Birmingham, UK. [60]Cancer Research UK Manchester Institute Cancer Biomarker Centre, University of Manchester, Manchester, UK. [61]Leicester Medical School, University of Leicester, Leicester, UK. [62]Division of Cancer Sciences, The University of Manchester and The Christie NHS Foundation Trust, Manchester, UK. [63]NHS Greater Glasgow and Clyde Pathology Department, Queen Elizabeth University Hospital, Glasgow, UK. [64]University of Leicester, Leicester, UK. [65]University of Manchester, Manchester, UK. [66]Institute of Immunology and Immunotherapy, University of Birmingham, UK. [67]Department of Medical Oncology, Aberdeen Royal Infirmary NHS Grampian, Aberdeen, UK. [68]University of Aberdeen, Aberdeen, UK. [69]Cancer Research Centre, University of Leicester, Leicester, UK. [70]Garvan Institute of Medical Research, New South Wales, Australia. [71]Aberdeen Royal Infirmary NHS Grampian, Aberdeen, UK. [72]Artificial Intelligence in Medicine (AIM) Program, Mass General Brigham, Harvard Medical School, Boston, MA, USA. [73]Department of Radiation Oncology, Brigham and Women's Hospital, Dana-Farber Cancer Institute, Harvard Medical School, Boston, MA, USA. [74]Radiology and Nuclear Medicine, CARIM & GROW, Maastricht University, Maastricht, The Netherlands. [75]Singleton Hospital, Swansea Bay University Health Board, Swansea, UK. [76]Cancer Research UK Scotland Institute, Glasgow, UK. [77]Institute of Cancer Sciences, University of Glasgow, Glasgow, UK. [78]Integrative Cancer Genomics Laboratory, VIB Center for Cancer Biology, Leuven, Belgium. [79]VIB Center for AI & Computational Biology, Belgium. [80]Department of Oncology, KU Leuven, Leuven, Belgium. [81]Department of Oncology, University Hospital Southampton NHS Foundation Trust, Southampton,

UK. [82]Department of Pathology, Aberdeen Royal Infirmary NHS Grampian, Aberdeen, UK. [83]School of Cancer Sciences, University of Glasgow, Glasgow, UK. [84]Beatson Institute for Cancer Research, University of Glasgow, Glasgow, UK. [85]Queen Elizabeth University Hospital, Glasgow, UK. [86]Princess Alexandra Hospital, The Princess Alexandra Hospital NHS Trust, Harlow, UK. [87]Royal Surrey Hospital, Royal Surrey Hospitals NHS Foundation Trust, Guildford, UK. [88]University of Surrey, Guildford, UK. [89]Bioinformatics and Systems Biology, Method Development and Research Infrastructure, Robert Koch Institute, Berlin, Germany. [90]Liverpool Heart and Chest Hospital, Liverpool, UK. [91]Danish Cancer Institute, Copenhagen, Denmark. [92]Computational Health Informatics Program, Boston Children's Hospital, Boston, MA, USA. [93]Department of Physics of Complex Systems, ELTE Eötvös Loránd University, Budapest, Hungary. [94]University Hospital Southampton NHS Foundation Trust, Southampton, UK. [95]Division of Infection, Immunity and Respiratory Medicine, University of Manchester, Manchester, UK. [96]Department of Pathology, ZAS Hospitals, Antwerp, Belgium. [97]Division of Research, Peter MacCallum Cancer Centre, Melbourne, Australia. [98]Institute for Computational Cancer Biology, Center for Integrated Oncology (CIO), Cancer Research Center Cologne Essen (CCCE), Faculty of Medicine and University Hospital Cologne, University of Cologne, Germany. [99]Berlin Institute for the Foundations of Learning and Data (BIFOLD), Berlin, Germany. [100]University of Sheffield, Sheffield, UK. [101]Sheffield Teaching Hospitals NHS Foundation Trust, Sheffield, UK. [102]Department of Pathology, Stanford University School of Medicine, Stanford, CA, USA. [103]Berlin Institute for Medical Systems Biology, Max Delbrück Center for Molecular Medicine in the Helmholtz Association (MDC), Berlin, Germany. [104]The University of Texas MD Anderson Cancer Center, Houston, TX, USA. [105]Department of Bioinformatics, Semmelweis University, Budapest, Hungary.

PEACE Consortium

Charles Swanton[1,2,3], Mariam Jamal-Hanjani[1,3,4], Nicholas McGranahan[1,5], Simone Zaccaria[1,6], Nnenna Kanu[7], Olivia Lucas[1,2,3,6], Sophia Ward[1,2,8], Rija Zaidi[1,6], Abigail Bunkum[1,4,6], Alexander Frankell[2], David Moore[3,7], Wing Kin Liu[1,4], Emilia L. Lim[1,2], Sonya Hessey[1,4,6], Cristina Naceur-Lombardelli[1], Andrew Rowan[2], Ariana Huebner[1,2,5], Carlos Martinez Ruiz[1,5], Clare Puttick[1,2,5], Corentin Richard[1], Dhruva Biswas[1,2,18], James Black[1,2,5], Jeanette Kittel[1,4], Katey Enfield[1,2], Kerstin Haase[1,4], Kerstin Thol[1,5], Kristiana Grigoriadis [1,2,5], Lucrezia Patruno[1,6], Maise Al-Bakir[1,2], Michelle Dietzen[1,5], Monica Sivakumar[1], Selvaraju Veeriah[1], Sergio Quezada[1,30], Supreet Kaur Bola[1,30], Takahiro Karasaki[1,2,4,32], William Hill[1,2], Alistair Magness[2], Chris Bailey[2], Claudia Lee[2], Emma Colliver[2], Foteini Athanasopoulou[2], Krijn Dijkstra[2], Mihaela Angelova[2], Oriol Pich[2], Roberto Vendramin[2], Vittorio Barbe[2], Anuradha Jayaram[3,7], Caroline Stirling[3], Chi-wah Lok[3], Constantine Alifrangis[3], Daniel Hochhauser[3,7], Dionysis Papadatos-Pastos[3], Gerhardt Attard[3,7], Heather Shaw[3,20], Ian Proctor[3], Jayant Rane[3], John Bridgewater[3], Kai-Keen Shiu[3], Kerry Bowles[3], Martin Forster[3,7], Melek Akay[3], Miriam Mitchison[3], Paddy Stone[3], Peter Ellery[3], Ron Sinclair[3], Sam Janes[3], Sarah Benafif[3,29], Sebastian Brandner[3], Siow-Ming Lee[3,7], Tanya Ahmad[3], Ursula McGovern[3], Zoe Rhodes[3], Imran Noorani[4,12,22], Othman Al-Sawaf[4], A.M. Mahedi Hasan[7], Adrienne Flanagan[7], Angela Dwornik[7], Anna Wingate[7], Antonia Toncheva[7], Benny Chain[7], Blanca Trujillo Alba[7], Claudia Peinador Marin[7], Crispin Hiley[7], Daniel Wetterskog[7], David R. Pearce[7], Gianmarco Leone[7], James Reading[7], Jie Min Lam[7], Kevin Litchfield[7], Mark Linch[7], Neil Magno[7], Osvaldas Vainauskas[7], Paulina Prymas[7], Piotr Pawlik[7], Robert E. Hynds[7], Samuel Gamble[7], Seng Kuong Ung[7], Sophia Wong[7], Stefano Lise[7], Tariq Enver[7], Teerapon Sahwangarrom[7], Jerome Nicod[8], Adrian Tookman[9,10], Faye Gishen[10], Aida Murra[11], Analyn Lucanas[11], Andreas Michael Schmitt[11], Andrew Furness[11], Arash Latifoltojar[11,15], Benjamin Shum[11,16], Brian Hanley[11,12,15], Camille Gerard[11,12], Charlotte Grieco[11], Charlotte Lewis[11], Charlotte Milner-Watts[11], Charlotte Spencer[11,12], Christina Messiou[11,15], Denise Kelly[11], Eleanor Carlyle[11], Emma Turay[11], Haixi Yan[11,12], James Larkin[11,15], Jennifer Biano[11], Justine Korteweg[11], Karla Pearce[11], Kate Young[11], Kayleigh Kelly[11], Kema Peat[11], Kim Edmonds[11], Lauren Grostate[11], Lauren Terry[11], Lewis Au[11,12], Lisa Pickering[11], Lucy Holt[11], Lyra Del Rosario[11], Mary Mangwende[11], Max Emmerich[11,12], Mo Linh Le[11], Molly O'Flaherty[11], Nadia Yousaf[11], Nikki Hunter[11], Olivia Curtis[11], Paolo Davide D'Arienzo[11,12], Peter Hill[11,25], Samra Turajlic[11,15,16], Sanjay Popat[11], Scott Shepherd[11,12], Steve Hazell[11], Zayd Tippu[11,12], Alexander Coulton[12], Anne-Laure Cattin[12], Annika Fendler[12], Daqi Deng[12], Fiona Byrne[12], Gordon Stamp[12], Hugang Feng[12], Husayn Pallikonda[12], Irene Lobon[12], Peter Parker[12], Allan Hackshaw[13], Anne-Marie Hacker[13], Aoife Walker[13], Hayley Bridger[13], Rachel Leslie[13], Andrew Tutt[14], Anna Green[14], Deborah Enting[14], Debra Josephs[14], Eleni (Lena) Karapanagiotou[14], Elias Pintus[14], Georgina Pulman[14], Natasha Wright[14], Ruby Stewart[14], Ruxandra Mitu[14], Sarah Howlett[14], Sarah Rudman[14], Sharmistha Ghosh[14], Sheeba Irshad[14], Sherene Phillips-Boyd[14], Ula Mahadeva[14], Blanche Hampton[17], Mairead McKenzie[17], Emma Nye[19], Iain McNeish[21], James Spicer[23], Mary Falzon[24], Teresa Marafioti[24], Peter Van Loo[26,27,28],

Stephan Beck[31], Will Drake[33], Alison Cluroe[34], Anna Paterson[34], Elena Provenzano[34], Kieren Allinson[34], Merche Jimenez-Linan[34], Sarah Aitken[34,56], Ultan McDermott[34,50], Amy Kerr[35], Andrew Robinson[35], Aya Osman[35], Bernard Olisemeke[35], Bruce Tanchel[35], Charlotte Ferris[35], Dr Peter Colloby[35], Gary Middleton[35,44], Gerald Langman[35], Helen Shackleford[35], Hollie Bancroft[35], Ian Tomlinson[35], Joanne Webb[35], Martin Collard[35], Peter Cockcroft[35], Rodelaine Wilson[35], Salma Kadiri[35], Shobhit Baijal[35], Ana Ortega-Franco[36], Fabio Gomes[36], Fiona Blackhall[36], Jo Dransfield[36], Mat Carter[36], Matthew Krebs[36,49], Pedro Oliveira[36], Yvonne Summers[36], Anne Thomas[37], Cathy Richards[37], Charlotte Poile[37], Dean Fennell[37], Jacqui Shaw[37], Jens Claus Hahne[37], Babu Naidu[38], Carlos Caldas[39], Emma Beddowes[39], James Brenton[39], Caroline Dive[40,41], Claire Wilson[42], Domenic Marrone[43], Grant Stewart[45], Hardeep Mudhar[46], John Le Quesne[47], Kevin Blyth[47], Patricia Roxburgh[47], Sioban Fraser[47], Lavinia Spain[48], Olaf Ansorge[50], Peter Campbell[50], Peter Ellery[51], Rebecca Fitzgerald[52], Roberto Salgado[53,54], Samantha Holden[55], Sanjay Jogai[55], Tania Fernandes[55], Thomas B.K. Watkins[57], Tim Maughan[58].

[1]Cancer Research UK Lung Cancer Centre of Excellence, University College London Cancer Institute, London, UK. [2]Cancer Evolution and Genome Instability Laboratory, The Francis Crick Institute, London, UK. [3]University College London Hospitals, London, UK. [4]Cancer Metastasis Laboratory, University College London Cancer Institute, London, UK. [5]Cancer Genome Evolution Research Group, University College London Cancer Institute, London, UK. [6]Computational Cancer Genomics Research Group, University College London Cancer Institute, London, UK. [7]University College London Cancer Institute, London, UK. [8]Genomics Science Technology Platform, The Francis Crick Institute, London, UK. [9]Marie Curie Hospice, London, UK. [10]Royal Free London NHS Foundation Trust, London, UK. [11]The Royal Marsden Hospital, London, UK. [12]The Francis Crick Institute, London, UK. [13]Cancer Research UK & UCL Cancer Trials Centre, London, UK. [14]Guy's and St Thomas' NHS Foundation Trust, London, UK. [15]The Institute of Cancer Research, London, UK. [16]Cancer Dynamics Laboratory, The Francis Crick Institute, London, UK. [17]Independent Cancer Patients' Voice, London, UK. [18]Bill Lyons Informatics Centre, University College London Cancer Institute, London, UK. [19]Experimental Histopathology, The Francis Crick Institute, London, UK. [20]Mount Vernon Cancer Centre, Northwood, UK. [21]Imperial College London, London, UK. [22]Department of Neurosurgery, National Hospital for Neurology and Neurosurgery, UK. [23]King's College London, London, UK. [24]Department of Cellular Pathology, University College London Hospitals, London, UK. [25]Imperial College London NHS Foundation Trust, London, UK. [26]Department of Genetics, The University of Texas MD Anderson Cancer Center, Houston, TX, USA. [27]Department of Genomic Medicine, The University of Texas MD Anderson Cancer Center, Houston, TX, USA. [28]Cancer Genomics Laboratory, The Francis Crick Institute, London, UK. [29]The Whittington Hospital NHS Trust, London, UK. [30]Immune Regulation and Tumour Immunotherapy Group, Cancer Immunology Unit, Research Department of Haematology, University College London Cancer Institute, London, UK. [31]Medical Genomics, University College London Cancer Institute, London, UK. [32]Department of Thoracic Surgery, Respiratory Center, Toranomon Hospital, Tokyo, Japan. [33]Barts Cancer Institute, Queen Mary University of London, London, UK. [34]Addenbrooke's Hospital, Cambridge University Hospitals, Cambridge, UK. [35]University Hospital Birmingham NHS Foundation Trust, Birmingham, UK. [36]The Christie NHS Foundation Trust, Manchester, UK. [37]University Hospitals of Leicester NHS Trust, Leicester, UK. [38]Birmingham Acute Care Research Group, Institute of Inflammation and Ageing, University of Birmingham, Birmingham, UK. [39]Cancer Research UK Cambridge Institute, University of Cambridge, Cambridge, UK. [40]Cancer Research UK Manchester Institute Cancer Biomarker Centre, University of Manchester, Manchester, UK. [41]Cancer Research UK Lung Cancer Centre of Excellence, University of Manchester, Manchester, UK. [42]Leicester Medical School, University of Leicester, Leicester, UK. [43]University of Manchester, Manchester, UK. [44]Institute of Immunology and Immunotherapy, University of Birmingham, UK. [45]Cambridge University Hospitals, Cambridge, UK. [46]Sheffield Teaching Hospitals NHS Foundation Trust, Sheffield, UK. [47]Beatson West of Scotland Cancer Centre, Glasgow, UK. [48]Peter MacCallum Cancer Institute, Melbourne, Australia. [49]Division of Cancer Sciences, The University of Manchester, Manchester, UK. [50]Wellcome Sanger Institute, Hinxton, UK. [51]Leeds Teaching Hospitals NHS Trust, Leeds, UK. [52]Early Cancer Institute, Department of Oncology, University of Cambridge, Cambridge, UK. [53]Department of Pathology, ZAS Hospitals, Antwerp, Belgium. [54]Division of Research, Peter MacCallum Cancer Centre, Melbourne, Australia. [55]University Hospital Southampton NHS Trust, Southampton, UK. [56]MRC Toxicology Unit, University of Cambridge, Cambridge, UK. [57]Department of Pathology, Stanford University School of Medicine, Stanford, CA, USA. [58]MRC Oxford Institute for Radiation Oncology, University of Oxford, Oxford, UK.
