# Supplementary material for: Characterizing the evolutionary dynamics of cancer proliferation in single-cell clones with SPRINTER
Source: Nat Genet. 2024 Nov 29;57(1):103–14. doi: 10.1038/s41588-024-01989-z (PMC11735394; doi:10.1038/s41588-024-01989-z)
Supplement: Supplementary file 2 — Reporting Summary [file 41588_2024_1989_MOESM2_ESM.pdf]

nature portfolio

Corresponding author(s): Nnennaya Kanu
Charles Swanton
Simone Zaccaria

Last updated by author(s): Oct 3, 2024

Reporting Summary

Nature Portfolio wishes to improve the reproducibility of the work that we publish. This form provides structure for consistency and transparency in reporting. For further information on Nature Portfolio policies, see our Editorial Policies and the Editorial Policy Checklist.

Statistics

For all statistical analyses, confirm that the following items are present in the figure legend, table legend, main text, or Methods section.

n/a	Confirmed	
☐	☒	The exact sample size (*n*) for each experimental group/condition, given as a discrete number and unit of measurement
☐	☒	A statement on whether measurements were taken from distinct samples or whether the same sample was measured repeatedly
☐	☒	The statistical test(s) used AND whether they are one- or two-sided<br>*Only common tests should be described solely by name; describe more complex techniques in the Methods section.*
☐	☒	A description of all covariates tested
☐	☒	A description of any assumptions or corrections, such as tests of normality and adjustment for multiple comparisons
☐	☒	A full description of the statistical parameters including central tendency (e.g. means) or other basic estimates (e.g. regression coefficient) AND variation (e.g. standard deviation) or associated estimates of uncertainty (e.g. confidence intervals)
☐	☒	For null hypothesis testing, the test statistic (e.g. *F*, *t*, *r*) with confidence intervals, effect sizes, degrees of freedom and *P* value noted<br>*Give P values as exact values whenever suitable.*
☐	☒	For Bayesian analysis, information on the choice of priors and Markov chain Monte Carlo settings
☐	☒	For hierarchical and complex designs, identification of the appropriate level for tests and full reporting of outcomes
☐	☒	Estimates of effect sizes (e.g. Cohen's *d*, Pearson's *r*), indicating how they were calculated

Our web collection on statistics for biologists contains articles on many of the points above.

Software and code

Policy information about availability of computer code

Data collection

Quality control of raw sequencing data (FASTQ):
FastQC (v0.11.8)
FastQ Screen (v0.13.0, flags: --subset 100000; --aligner bowtie2)
MultiQC (v1.10.1)

Removing adapters:
fastp (v0.20.0)

Alignment to hg19 genome assembly:
BWA-MEM (v0.7.17)

Deduplication of sequencing reads:
sambamba markdup (v0.7.0, flags: --remove-duplicates)

Merging aligned sequencing reads:
sambamba merge (v0.7.0)

Quality control of aligned sequencing reads (BAM):
Samtools (v1.9)
Picard (v2.25.4) with MultiQC (v1.10.1)

Counting single-cell sequencing reads:
CHISEL (v1.1.4)

Calling genomic variants:
Mutect2 (GATK, v4.2.0)

Variant annotation:
Ensembl Variant Effect Predictor (VEP, v109) with the plugins CADD (v16), LOFTEE, and SpliceAI
openCRAVAT34 (v2.3.0) with CHASMplus, CHASMplus LUAD, and CHASMplus LUSC modules

Data analysis

New method for identification and clone assignment of S and G2 phase cells:
SPRINTER (v1.0) available on GitHub at https://github.com/zaccaria-lab/sprinter with reproducible capsule linked to this manuscript available on CodeOcean

Existing methods for S phase identification:
cell cycle classifier (CCC) with HMMcopy (v0.6.46)
MAPD, available on GitHub at https://github.com/TheKorenLab/Single-cell-replication-timing (commit 4773a8f)

Phylogenetic analysis:
HUNTRESS (v0.1.2)
MEDICC2 (v1.0.2)

Metastatic dissemination analysis:
MACHINA (v1.2)

For manuscripts utilizing custom algorithms or software that are central to the research but not yet described in published literature, software must be made available to editors and reviewers. We strongly encourage code deposition in a community repository (e.g. GitHub). See the Nature Portfolio guidelines for submitting code & software for further information.

Data

Policy information about availability of data

All manuscripts must include a data availability statement. This statement should provide the following information, where applicable:

- Accession codes, unique identifiers, or web links for publicly available datasets
- A description of any restrictions on data availability
- For clinical datasets or third party data, please ensure that the statement adheres to our policy

Raw scDNA-seq data generated in this study from the ground truth datasets have been deposited at the NCBI Sequence Read Archive (SRA) under accession code PRJNA1158752. Raw scDNA-seq data generated in this study from the patient enrolled in the TRACERx and PEACE studies have been deposited at the European Genome–phenome Archive (EGA) under accession code EGAD00001015411. Access is controlled by the TRACERx and PEACE data access committees, who assess whether the proposed research is allowed given patient consent and ethical approvals, as well as the scientific purpose. Details on how to apply for access are available on EGA. The processed data for the figures and analyses performed in this study are available in Zenodo at https://doi.org/10.5281/zenodo.13754278. The processed data and related genomic variants from the previous TNBC and HGSC datasets are available in Zenodo at https://doi.org/10.5281/zenodo.6998936 and https://doi.org/10.5281/zenodo.7718917 as part of previous studies. Raw scDNA-seq data generated in a previous study from phase-sorted lymphoblastoid cells are available in SRA under accession code PRJNA770772.

Research involving human participants, their data, or biological material

Policy information about studies with human participants or human data. See also policy information about sex, gender (identity/presentation), and sexual orientation and race, ethnicity and racism.

Reporting on sex and gender	Done as part of the previous TRACERx study (https://doi.org/10.1038/s41586-023-05783-5).
Reporting on race, ethnicity, or other socially relevant groupings	Done as part of the previous TRACERx study (https://doi.org/10.1038/s41586-023-05783-5).
Population characteristics	The patient was a 60-year-old male with stage IIIA squamous cell carcinoma, who was part of the TRACERx study and underwent surgical removal of the primary tumour and who subsequently relapsed and died 251 days later after receiving multiple lines of chemotherapy and radiotherapy. The patient died with metastases in multiple anatomical sites and was enrolled in the PEACE autopsy programme, through which a post-mortem examination was performed.
Recruitment	Done as part of the previous TRACERx study (https://doi.org/10.1038/s41586-023-05783-5).
Ethics oversight	Done as part of the previous TRACERx study (https://doi.org/10.1038/s41586-023-05783-5) and PEACE autopsy programme (detailed information are reported in Methods).

Note that full information on the approval of the study protocol must also be provided in the manuscript.

Field-specific reporting

Please select the one below that is the best fit for your research. If you are not sure, read the appropriate sections before making your selection.

- [x] Life sciences
- [ ] Behavioural & social sciences
- [ ] Ecological, evolutionary & environmental sciences

For a reference copy of the document with all sections, see nature.com/documents/nr-reporting-summary-flat.pdf

Life sciences study design

All studies must disclose on these points even when the disclosure is negative.

Sample size	The number of primary tumour and metastatic samples was chosen based on tissue availability from the related TRACERx study and PEACE autopsy programme. The number of cells sequenced per sample (2000-2500) was chosen based on previous power studies for scDNA-seq data (https://doi.org/10.1038/s41587-020-0661-6).
Data exclusions	Cells with less than 100,000 sequencing reads have been excluded from downstream analysis in this study because this low number of sequencing reads was insufficient for copy-number analysis and may indicate failures in the process of DNA library preparation as previously reported.
Replication	For reproducibility, the DNA sequencing reads of every cell, as well as the SPRINTER code and related guided demos to reproduce the results, will be made publicly available after review. Currently, an automatic reproducible capsule for SPRINTER's results is available in CodeOcean at: https://codeocean.com/capsule/9392115. The capsule can be accessed to review and verify previous automatically and independently tested executions of SPRINTER and re-execute it. The processed results to reproduce every figure and downstream analysis in the manuscript, including related demos, will be made publicly available in Zenodo.
Randomization	Randomization is not relevant as this is an observational study.
Blinding	Blinding is not relevant as this is an observational study.

Reporting for specific materials, systems and methods

We require information from authors about some types of materials, experimental systems and methods used in many studies. Here, indicate whether each material, system or method listed is relevant to your study. If you are not sure if a list item applies to your research, read the appropriate section before selecting a response.

Materials & experimental systems

n/a	Involved in the study	
☒	☐	Antibodies
☒	☐	Eukaryotic cell lines
☒	☐	Palaeontology and archaeology
☒	☐	Animals and other organisms
☒	☐	Clinical data
☒	☐	Dual use research of concern
☒	☐	Plants

Methods

n/a	Involved in the study	
☒	☐	ChIP-seq
☐	☒	Flow cytometry
☒	☐	MRI-based neuroimaging

Flow Cytometry

Plots

Confirm that:

- [x] The axis labels state the marker and fluorochrome used (e.g. CD4-FITC).
- [x] The axis scales are clearly visible. Include numbers along axes only for bottom left plot of group (a 'group' is an analysis of identical markers).
- [x] All plots are contour plots with outliers or pseudocolor plots.
- [x] A numerical value for number of cells or percentage (with statistics) is provided.

Methodology

Sample preparation	Flow Cytometry was used for generation of the ground truth datasets using the colorectal cell line HCT116, using one diploid and one tetraploid lineage. In detail, we first labelled cells with Click-iT EdU and fixed and stained using the Click-iT Plus EdU Flow Cytometry Assay Kit (C10634 Invitrogen), halting further progression through the cell cycle. Cells were stained with 2ug/ml Hoechst 33342 before flow sorting.

Instrument	Flow sorting was performed on a BD Influx cell sorter (BD, San Jose, CA, USA) using a 140 micron nozzle, with pressure maintained at 14 psi.
Software	Data was analysed using BD FACS Software v1.2.0.142 (BD, San Jose, CA, USA).
Cell population abundance	Cells were simultaneously and electrostatically sorted into 5 uniform fractions of different cell cycle phases (G1, early S, mid S, late S, and G2).
Gating strategy	Cells were simultaneously and electrostatically sorted based on both EdU (Alexa Fluor 647, excited with a 642nm laser and emission collected in a 670/30BP filter) and DNA Hoechst 33342 dye (excited using a 405nm laser and emission collected in a 460/50BP filter), with both parameters displayed on a linear scale.

☒ Tick this box to confirm that a figure exemplifying the gating strategy is provided in the Supplementary Information.
